# Design, synthesis, and biological evaluation of novel carbazole derivatives as potent DNMT1 inhibitors with reasonable PK properties

**DOI:** 10.1080/14756366.2022.2079640

**Published:** 2022-06-07

**Authors:** Ennian Li, Kai Wang, Bei Zhang, Siqi Guo, Senhao Xiao, Qi Pan, Xiaowan Wang, Weiying Chen, Yunshan Wu, Hesong Xu, Xiangqian Kong, Cheng Luo, Shijie Chen, Bo Liu

**Affiliations:** a Guangdong Provincial Key Laboratory of Clinical Research on Traditional Chinese Medicine Syndrome, The Second Clinical Medical College, Guangzhou University of Chinese Medicine, Guangzhou, China; b State Key Laboratory of Drug Research, The Center for Chemical Biology, Drug Discovery and Design Center, Shanghai Institute of Materia Medica, Chinese Academy of Sciences, Shanghai, China; c School of Pharmacy, Nanchang University, Nanchang, China; d Guangzhou Key Laboratory of Chirality Research on Active Components of Traditional Chinese Medicine, Guangzhou, China; e School of Pharmaceutical Science and Technology, Hangzhou Institute for Advanced Study, University of Chinese Academy of Sciences, Hangzhou, China; f Guangzhou Institute of Biomedicine and Health, Chinese Academy of Sciences, Guangzhou, China; g State Key Laboratory of Dampness Syndrome of Chinese Medicine, Guangzhou, China; h Guangdong-Hong Kong-Macau Joint Lab on Chinese Medicine and Immune Disease Research, Guangzhou University of Chinese Medicine, Guangzhou, China

**Keywords:** DNMT1 inhibitor, A549 cell lines, HCT116 cell lines, antitumor activity, pharmacokinetic

## Abstract

The DNA methyltransferases (DNMTs) were found in mammals to maintain DNA methylation. Among them, DNMT1 was the first identified, and it is an attractive target for tumour chemotherapy. DC_05 and DC_517 have been reported in our previous work, which is non-nucleoside DNMT1 inhibitor with low micromolar IC_50_ values and significant selectivity towards other S-adenosyl-_L_-methionine (SAM)-dependent protein methyltransferases. In this study, through a process of similarity-based analog searching, a series of DNMT1 inhibitors were designed, synthesized, and evaluated as anticancer agents. SAR studies were conducted based on enzymatic assays. And most of the compounds showed strong inhibitory activity on human DNMT1, especially WK-23 displayed a good inhibitory effect on human DNMT1 with an IC_50_ value of 5.0 µM. Importantly, the pharmacokinetic (PK) profile of WK-23 was obtained with quite satisfying oral bioavailability and elimination half-life. Taken together, WK-23 is worth developing as DNMT1-selective therapy for the treatment of malignant tumour.

## Graphical Abstract

**Figure:**
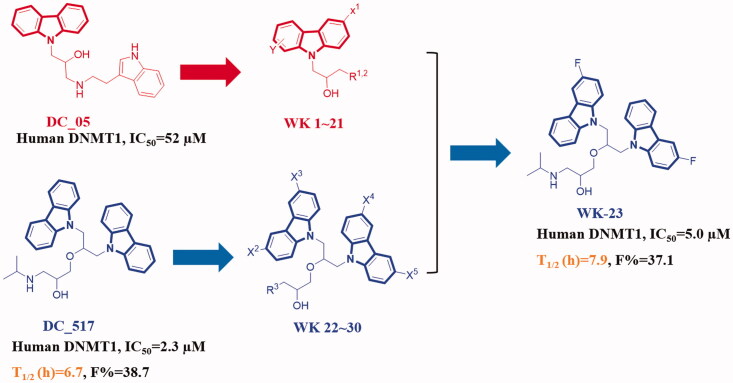

## 1. Introduction

Epigenetic modification, like DNA methylation, plays a major role in the expression of genetic information. Methylation of DNA at C-5 of cytosine is one of the most studied modifications of the mammalian genome^1^. DNMTs (DNA methyltransferases), which include DNMT1, DNMT3A, and DNMT3B, have been identified in humans^2^. DNMT1 is the most abundant among the three and is responsible for the maintenance of CpG methylation patterns in mammals with hemimethylated CpG dinucleotides serving as preferred substrates^3^. DNMT1 plays a significant role in the structural modification of chromosomes and the regulation of gene expression^1^. The methylation of the 5-carbon on cytosine residues (5mC) in CpG dinucleotides was the first described covalent modification of DNA and is one of the most extensively characterised modifications of chromatin, and thus, DNMT inhibitors have become useful tools for treating cancers^4^.

**Figure 1.**
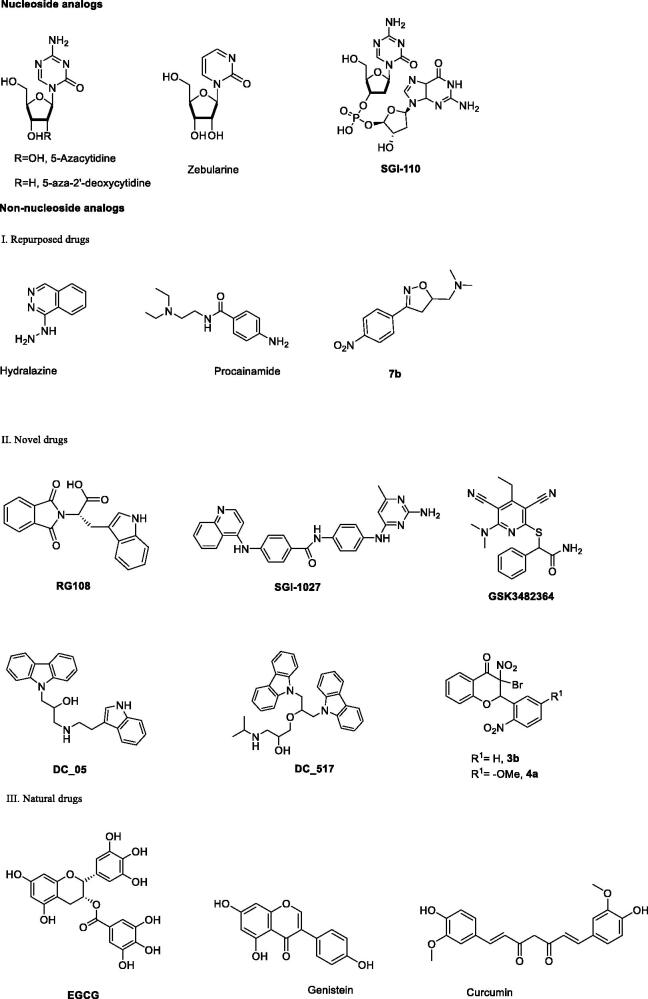
The approved or clinically investigated DNMT inhibitors.

So far, two types of DNMT1 inhibitors have been thus found, namely nucleoside analogs and non-nucleoside analogs. Nucleoside analogs, such as the U.S. Food and Drug Administration (FDA) approved 5-azacytidine and 5-aza-2′-deoxycytidine^5^, or 2-pyrimidone-1-*β*-_D_–riboside (zebularine)^6^, or the dinucleotide derivative **SGI-110**^7^, exert their effects by incorporation into DNA inducing substantial DNA methylation inhibition and reactivation of hypermethylated genes. However, these drugs are unstable, show low specificity, and have obvious toxic side effects^8^. Therefore, specific concern has been given about non-nucleosides. As shown in Figure 1, various non-nucleoside analogs have been reported, including the followed natural compounds, such as genistein^9^, (-)-epigallocatechin 3-*O*-gallate (**EGCG**)^10^, and curcumin^11^; repurposed drugs, such as the antihypertensive drug hydralazine^12^, procainamide^13^, and **7b**^14^; novel inhibitors, such as phthalimido-_L_-tryptophan (**RG108**)^15^, the quinolone derivative **SGI-1027**^16^, and the recently reported selective DNMT1 inhibitor **GSK3482364**^17^, and three small molecules identify from a DNMT focussed library including **CSC027480404**, **CSC026286840**, and **CSC027694519**^18^. Another recent research identified two 3-bromo-3-nitroflavanones **3b** and **4a** as potent non-nucleoside DNMT inhibitors with good activity and stability targeting DNA methylation^19^. Compared with nucleoside analogs, non-nucleoside analogs are less likely to be incorporated into DNA and hence provide a relatively safe method to target DNA methylation.

There are two non-nucleoside analogs **DC_05** and **DC_517** have been reported as specific and highly potent inhibitors of DNMT1 *via* biochemical and cellular assays^20^. With an aim to improve PK properties, and decreased the side effect and toxicity, we designed and synthesised a series of derivatives based on the structures of **DC_05** and **DC_517**. According to the DNMT1 enzyme inhibition assays, we obtained **WK-23** as a specific DNMT1 inhibitor with excellent inhibitory activity (IC_50_ = 5.0 µM). In the further *in vivo* pharmacokinetic (PK) study, **WK-23** displayed a good plasma exposure and an acceptable oral bioavailability of *F*% = 37.1.

## 2. Materials and methods

### 2.1. Chemistry

All anhydrous reactions were performed under a nitrogen atmosphere. The reaction progress was monitored by thin layer-chromatography (TLC) using silica gel F254 plates. Melting points (uncorrected) were determined on an XRC-1 micro melting point apparatus. Infra-red spectra (IR) were recorded on a PerkinElmer Spectrum Two FT-IR instrument. High-resolution mass spectra (HRMS) were taken on a Thermo-Fisher LTQ Orbitrap XL instrument. The ^1^H and ^13 ^C NMR (nuclear magnetic resonance spectra) experiments were performed by Bruker AM-600 spectrometer using TMS (tetramethylsilane) as the internal standard. Column chromatography was run on 200–300 mesh silica gel from Qingdao Ocean Chemicals (Qingdao, Shandong, China). Unless otherwise indicated, all materials were obtained from commercially available sources and used without further purification.

### 2.2. General procedures

#### 2.2.1. 2-Chloro-N-(4-fluorophenyl) aniline (a2)

To a mixture of NaO*^t^*Bu (24.00 g, 250.0 mmol), [HP*^t^*Bu_3_][BF_4_] (1.02 g, 3.5 mmol) and Pd(OAc)_2_ (560.0 mg, 2.5 mmol) in toluene (200 ml) and stirred to disperse it well. Then added 2-chloraniline (6.40 g, 50.0 mmol) (**a1**) and 4-bromofluorobenzene (8.75 g, 50.0 mmol). After the addition was completed, the temperature was raised to 110 °C and refluxed for 4 h under a nitrogen atmosphere. After completion of the reaction as indicated by TLC (PE/EA = 20:1) (petroleum ether/ethyl acetate), cool to room temperature, quenched by water (100 ml), extracted with EtOAc (ethyl acetate) (200 ml × 2). The organic layers were combined and washed with brine, dried with anhydrous Na_2_SO_4_, and evaporated *in vacuo* to obtain the crude products of 12.60 g. The crude products were further purified by column chromatography with an eluting system of petroleum ether (100%) to give **a2** as a light-yellow liquid (10.30 g) in a 93% yield.

Liquid. HRMS-ESI calcd. for C_12_H_10_FNCl [M + H]^+^ 222.0486, found 222.0533.

^1^H NMR (600 MHz, DMSO-d_6_) *δ* 7.64 (s, 1H), 7.40 (dd, *J* = 8.0, 1.4 Hz, 1H), 7.17 (td, *J* = 7.7, 7.2, 1.5 Hz, 1H), 7.15–7.08 (m, 5H), 6.86 (ddd, *J* = 8.8, 6.3, 1.6 Hz, 1H).

^13^C NMR (151 MHz, DMSO-d_6_) *δ* 157.62 (d, *J* = 237.3 Hz), 141.33, 139.45 (d, *J* = 2.2 Hz), 130.43, 128.27, 122.63, 121.54, 121.37 (d, *J* = 7.8 Hz), 117.85, 116.12 (d, *J* = 22.3 Hz).

#### 2.2.2. 2-Chloro-4-fluoro-N-(4-fluorophenyl)aniline (b2)

Liquid. HRMS-ESI calcd. for C_12_H_9_F_2_NCl [M + H]^+^ 240.0392, found 240.0440.

^1^H NMR (600 MHz, DMSO-d_6_) *δ* 7.63 (s, 1H), 7.42 (dd, *J* = 8.6, 3.0 Hz, 1H), 7.18 (dd, *J* = 9.0, 5.5 Hz, 1H), 7.13–7.03 (m, 3H), 7.01–6.94 (m, 2H).

^13^C NMR (151 MHz, DMSO-d_6_) *δ* 157.21 (d, *J* = 236.3 Hz), 156.71 (d, *J* = 240.6 Hz), 140.35 (d, *J* = 2.1 Hz), 137.91 (d, *J* = 2.7 Hz), 124.61 (d, *J* = 10.6 Hz), 120.79 (d, *J* = 8.4 Hz), 119.86 (d, *J* = 7.8 Hz), 117.45 (d, *J* = 25.6 Hz), 116.11 (d, *J* = 22.3 Hz), 115.29 (d, *J* = 21.8 Hz).

#### 2.2.3. General procedure for intermediates a3 and b3

##### 2.2.3.1. 3-Fluoro-9H-carbazole (a3)

To a mixture of NaO*^t^*Bu (21.87 g, 227.8 mmol), [HP*^t^*Bu_3_][BF_4_] (925.0 mg, 3.2 mmol) and Pd(OAc)_2_ (510.0 mg, 2.3 mmol) in 1,4-dioxane (250 ml) and stirred to disperse it well. Then added **a2** (10.07 g, 45.5 mmol). After the addition was completed, the temperature was raised to 110 °C and refluxed for 4 h under a nitrogen atmosphere. After completion of the reaction as indicated by TLC (PE/EA = 20:1), cool to room temperature and quenched by water (100 ml), extracted with EtOAc (200 ml × 2). The organic layers were combined and washed with brine, dried with anhydrous Na_2_SO_4_, and evaporated *in vacuo* to give the grey black crude products 10.20 g. The crude products were further purified by column chromatography with an eluting system of PE/EA (100:1–20:1). The residue after concentration *in vacuo* was triturated with petroleum ether, the mixture was filtered to obtain a white solid **a3** 3.80 g in 45% yield.

m.p. 134–136 °C. HRMS-ESI calcd. for C_12_H_9_FN [M + H]^+^ 186.0719, found 186.0767.

^1^H NMR (600 MHz, DMSO-d_6_) *δ* 11.30 (s, 1H), 8.12 (d, *J* = 7.8 Hz, 1H), 7.95 (dd, *J* = 9.4, 2.6 Hz, 1H), 7.53–7.45 (m, 2H), 7.40 (ddd, *J* = 8.2, 7.0, 1.2 Hz, 1H), 7.23 (td, *J* = 9.1, 2.6 Hz, 1H), 7.19–7.09 (m, 1H).

^13^C NMR (151 MHz, DMSO-d_6_) *δ* 156.83 (d, *J* = 232.2 Hz), 141.2, 136.63, 126.57, 123.29 (d, *J* = 9.8 Hz), 122.62 (d, *J* = 4.2 Hz), 121.10, 118.89, 113.60 (d, *J* = 25.3 Hz), 112.22 (d, *J* = 9.3 Hz), 111.66, 106.17 (d, *J* = 23.6 Hz).

##### 2.2.3.2. 3,6-Difluoro-9H-carbazole (b3)

m.p. 149–151 °C. HRMS-ESI calcd. for C_12_H_8_F_2_N [M + H]^+^ 204.0625, found 204.0672.

^1^H NMR (600 MHz, DMSO-d_6_) *δ* 11.34 (s, 1H), 7.97 (dd, *J* = 9.4, 2.6 Hz, 2H), 7.49 (dd, *J* = 8.8, 4.4 Hz, 2H), 7.26 (td, *J* = 9.1, 2.6 Hz, 2H).

^13^C NMR (151 MHz, DMSO-d_6_) *δ* 156.63 (d, *J* = 232.4 Hz), 137.69, 122.96 (dd, *J* = 10.0, 4.3 Hz), 114.39 (d, *J* = 25.5 Hz), 112.62 (d, *J* = 9.2 Hz), 106.53 (d, *J* = 23.8 Hz).

#### 2.2.4. General procedure for intermediates a4 and b4

##### 2.2.4.1. 3-Fluoro-9-(oxiran-2-ylmethyl)-9H-carbazole (a4)

At ambient temperature, **a3** (2.40 g, 13.0 mmol) was dissolved in DMF (50 ml), followed by the addition of KOH (0.80 g, 14.3 mmol). After 5 min stirring under 0 °C, epichlorohydrin (2.40 g, 26.0 mmol) was added dropwise. Then the mixture was warmed to room temperature and stirred for another 3 h. After **a3** was completely consumed, the solution was poured into 20 ml water, and extracted with EtOAc (50 ml × 2). The organic layers were combined and washed with brine, dried with anhydrous Na_2_SO_4_, and evaporated *in vacuo* to give 3.80 g of black liquid crude product. The crude products were further purified by column chromatography with an eluting system of PE/EA (80:1–20:1) to give off brown solid **a4** 1.90 g in 60% yield.

m.p. 39–41 °C. HRMS-ESI calcd. for C_15_H_13_FNO [M + H]^+^ 242.0981, found 242.1030.

^1^H NMR (600 MHz, DMSO-d_6_) *δ* 8.16 (d, *J* = 7.8 Hz, 1H), 8.01 (dd, *J* = 9.2, 2.6 Hz, 1H), 7.70–7.63 (m, 2H), 7.51–7.44 (m, 1H), 7.31 (td, *J* = 9.2, 2.6 Hz, 1H), 7.24–7.16 (m, 1H), 4.80 (dd, *J* = 15.8, 3.1 Hz, 1H), 4.42 (dd, *J* = 15.9, 5.8 Hz, 1H), 3.31 (dq, *J* = 5.8, 3.1 Hz, 1H), 2.79–2.73 (m, 1H), 2.57 (dd, *J* = 5.1, 2.6 Hz, 1H).

^13^C NMR (151 MHz, DMSO-d_6_) *δ* 156.71 (d, *J* = 232.9 Hz), 141.22, 136.86, 126.34, 122.60 (d, *J* = 9.9 Hz), 121.74 (d, *J* = 4.1 Hz), 120.69, 118.97, 113.26 (d, *J* = 25.4 Hz), 110.68 (d, *J* = 9.2 Hz), 109.92, 105.86 (d, *J* = 23.8 Hz), 50.31, 44.64, 44.35.

##### 2.2.4.2. 3,6-Difluoro-9-(oxiran-2-ylmethyl)-9H-carbazole (b4)

m.p. 121–123 °C. HRMS-ESI calcd. for C_15_H_12_F_2_NO [M + H]^+^ 260.0887, found 260.0932.

^13^C NMR (151 MHz, DMSO-d_6_) *δ* 156.98 (d, *J* = 233.3 Hz), 138.22, 122.58 (dd, *J* = 10.1, 4.1 Hz), 114.53 (d, *J* = 25.5 Hz), 111.55 (d, *J* = 9.1 Hz), 106.70 (d, *J* = 24.0 Hz), 50.79, 45.08, 45.01.

#### 2.2.5. General procedure for intermediates d1, d2, d3

##### 2.2.5.1. 1,3-Bis(3-fluoro-9H-carbazol-9-yl)propan-2-ol (d1)

To solution of **a4** (0.90 g, 3.7 mmol) and **a3** (0.69 g, 3.7 mmol) in acetone 8 ml was added KOH (0.42 g, 7.5 mmol) and anhydrous Na_2_SO_4_ (0.53 g, 3.7 mmol). The resultant mixture was stirred at room temperature overnight. After TLC indicated the total conversion, water was added to quench the reaction and the mixture was washed with EtOAc (20 ml × 2). The organic layer was dried over anhydrous Na_2_SO_4_ and concentrated *in vacuo*. Flash column chromatography utilising PE/EA (10:1) as the eluting afforded **d1**.

Intermediates **d2** and **d3** were prepared like that described for **d1**.

m.p. 153–155 °C. HRMS-ESI calcd. for C_27_H_21_F_2_N_2_O [M + H]^+^ 427.1622, found 427.1670.

^1^H NMR (600 MHz, DMSO-d_6_) *δ* 8.15 (d, *J* = 7.7 Hz, 2H), 7.99 (dd, *J* = 9.2, 2.5 Hz, 2H), 7.63 (dd, *J* = 8.9, 4.3 Hz, 2H), 7.58 (d, *J* = 8.3 Hz, 2H), 7.43 (t, *J* = 7.6 Hz, 2H), 7.28 (td, *J* = 9.2, 2.5 Hz, 2H), 7.18 (t, *J* = 7.4 Hz, 2H), 5.27 (s, 1H), 4.60–4.46 (m, 4H), 4.40 (s, 1H).

^13^C NMR (151 MHz, DMSO-d_6_) *δ* 157.04 (d, *J* = 232.9 Hz), 141.86, 137.60, 126.62, 123.01 (d, *J* = 9.6 Hz), 122.21 (d, *J* = 4.0 Hz), 121.13, 119.16, 113.56 (d, *J* = 25.2 Hz), 111.21 (d, *J* = 9.1 Hz), 110.43, 106.25 (d, *J* = 23.7 Hz), 69.23, 47.62.

##### 2.2.5.2. 1,3-Bis(3,6-difluoro-9H-carbazol-9-yl) propan-2-ol (d2)

m.p. 207–209 °C. HRMS-ESI calcd. for C_27_H_19_F_4_N_2_O [M + H]^+^ 463.1434, found 463.1480.

^1^H NMR (600 MHz, DMSO-d_6_) *δ* 8.01 (dd, *J* = 9.2, 2.5 Hz, 4H), 7.67 (dd, *J* = 9.0, 4.3 Hz, 4H), 7.32 (td, *J* = 9.2, 2.6 Hz, 4H), 5.22 (s, 1H), 4.59 (dd, *J* = 14.9, 3.3 Hz, 2H), 4.50 (dd, *J* = 14.9, 8.7 Hz, 2H), 4.35 (s, 1H).

^13^C NMR (151 MHz, DMSO-d_6_) *δ* 156.84 (d, *J* = 232.9 Hz), 138.51, 122.52 (dd, *J* = 10.0, 4.3 Hz), 114.34 (d, *J* = 25.4 Hz), 111.65 (d, *J* = 9.0 Hz), 106.60 (d, *J* = 23.9 Hz), 69.26, 47.74.

##### 2.2.5.3. 1-(3,6-Difluoro-9H-carbazol-9-yl)-3-(3-fluoro-9H-carbazol-9-yl) propan-2-ol (d3)

m.p. 207–209 °C. HRMS-ESI calcd. for C_27_H_19_F_4_N_2_O [M + H]^+^ 463.1434, found 463.1480.

^1^H NMR (600 MHz, DMSO-d_6_) *δ* 8.01 (dd, *J* = 9.2, 2.5 Hz, 4H), 7.67 (dd, *J* = 9.0, 4.3 Hz, 4H), 7.32 (td, *J* = 9.2, 2.6 Hz, 4H), 5.22 (s, 1H), 4.59 (dd, *J* = 14.9, 3.3 Hz, 2H), 4.50 (dd, *J* = 14.9, 8.7 Hz, 2H), 4.35 (s, 1H).

^13^C NMR (151 MHz, DMSO-d_6_) *δ* 156.84 (d, *J* = 232.9 Hz), 138.51, 122.52 (dd, *J* = 10.0, 4.3 Hz), 114.34 (d, *J* = 25.4 Hz), 111.65 (d, *J* = 9.0 Hz), 106.60 (d, *J* = 23.9 Hz), 69.26, 47.74.

#### 2.2.6. 9,9′-(2-(Oxiran-2-ylmethoxy) propane-1,3-diyl) bis (3-fluoro-9H-carbazole) (d4)

Powder KOH (208.0 mg, 3.7 mmol) and anhydrous Na_2_SO_4_ (484.0 mg, 3.4 mmol) were added to a **d1** solution (1.45 g, 3.4 mmol) in acetone (20 ml) and stirred for 5 min at 0 °C. Epichlorohydrin (3.15 g, 34 mmol) was added dropwise and the resultant mixture was stirred at room temperature for 6 h. Upon completion, water (20 ml) was added to quench the reaction, and the mixture was partitioned between EtOAc and H_2_O. The aqueous layer was extracted by EtOAc, and the combined organics were washed with saturated aqueous NaCl, dried over Na_2_SO_4_, filtered, and concentrated *in vacuo*. The crude product was purified by flash column chromatography using PE/EA (50:1–10:1) as the eluent. White solid, yield 73%.

Intermediates **d5** and **d6** were prepared like that described for **d4**.

#### 2.2.7. General procedure for preparation of the target compounds (WK-1–WK-11)

##### 2.2.7.1. 1-((2-(1H-indol-3-yl)ethyl)amino)-3-(3-fluoro-9H-carbazol-9-yl)propan-2-ol (WK-1)

A mixture of intermediate compound **a4** (120.0 mg, 0.5 mmol, 1 equiv.) and aliphatic amines (318.7 mg, 2.0 mmol, 4 equiv.) dissolved in 5 ml EtOH, was introduced into a 10 ml sealed tube. The mixture was stirred at 60 °C and monitored by TLC until compound **a4** was completely consumed. The mixture was extracted with EtOAc and water. The organic layer was combined, washed with saturated NaCl, dried over anhydrous Na_2_SO_4_, and concentrated *in vacuo*. Final flash column chromatography utilising PE/EA (1:1) as the eluent afforded *1-((2-(1H-indol-3-yl)ethyl)amino)-3-(3-fluoro-9H-carbazol-9-yl)propan-2-ol* (**WK-1**) as a white solid, 64.0 mg (yield 32.10%). m.p. 137–139 °C. HRMS-ESI calcd. for C_25_H_25_FN_3_O [M + H]^+^ 402.1982, found 402.1962. Intermediates **WK-2**–**WK-9** were prepared in a procedure similar to that described for **WK-1**.

IR (KBr), ν, cm^−1^: 3273, 2930, 1487, 1459, 1439, 1351, 1281, 1170, 889, 795, 727.

m.p. 137–139 °C. HRMS-ESI calcd. for C_25_H_25_FN_3_O [M + H]^+^ 402.1982, found 402.1962.

^1^H NMR (600 MHz, DMSO-d_6_) *δ* 10.79 (s, 1H), 8.14 (d, *J* = 7.6 Hz, 1H), 7.97 (d, *J* = 8.9 Hz, 1H), 7.67–7.55 (m, 2H), 7.52 (d, *J* = 7.9 Hz, 1H), 7.42 (t, *J* = 7.6 Hz, 1H), 7.33 (d, *J* = 8.0 Hz, 1H), 7.23 (t, *J* = 8.1 Hz, 1H), 7.20–7.11 (m, 2H), 7.06 (t, *J* = 7.5 Hz, 1H), 6.97 (t, *J* = 7.4 Hz, 1H), 5.02 (s, 1H), 4.46 (dd, *J* = 14.9, 5.0 Hz, 1H), 4.27 (dd, *J* = 14.8, 6.8 Hz, 1H), 3.98 (s, 1H), 2.83 (dd, *J* = 22.5, 6.5 Hz, 4H), 2.61 (ddd, *J* = 44.5, 11.8, 5.5 Hz, 2H), 1.99 (brs, 1H).

^13^C NMR (151 MHz, DMSO-d_6_) *δ* 156.48 (d, *J* = 232.7 Hz), 141.45, 137.15, 136.24, 127.29, 126.08, 122.58, 122.38 (d, *J* = 9.9 Hz), 121.61 (d, *J* = 4.4 Hz), 120.79, 120.53, 118.52, 118.30, 118.10, 112.99 (d, *J* = 25.4 Hz), 112.59, 111.31, 110.72 (d, *J* = 8.9 Hz), 110.01, 105.62 (d, *J* = 23.8 Hz), 68.87, 52.98, 50.35, 47.19, 25.57.

##### 2.2.7.2. 1-(3-Fluoro-9H-carbazol-9-yl)-3-((4-fluorophenethyl)amino)propan-2-ol (WK-2)

White solid, 94.0 mg (yield 49%). IR (KBr), ν, cm^−1^: 3059, 2822, 1603, 1511, 1486, 1465, 1279, 1224, 1168, 890, 743.

m.p. 109–111 °C. HRMS-ESI calcd. for C_23_H_23_F_2_N_2_O [M + H]^+^ 381.1778, found 381.1762.

^1^H NMR (600 MHz, DMSO-d_6_) *δ* 8.14 (d, *J* = 7.7 Hz, 1H), 7.98 (dd, *J* = 9.3, 2.6 Hz, 1H), 7.59–7.56 (m, 2H), 7.43 (t, *J* = 8.2 Hz, 1H), 7.29–7.23 (m, 3H), 7.17 (t, *J* = 7.4 Hz, 1H), 7.09 (t, *J* = 8.9 Hz, 2H), 5.04 (s, 1H), 4.43 (dd, *J* = 14.8, 5.0 Hz, 1H), 4.25 (dd, *J* = 14.8, 6.8 Hz, 1H), 3.95 (t, *J* = 5.9 Hz, 1H), 2.77–2.67 (m, 4H), 2.57 (ddd, *J* = 38.6, 5.6 Hz, 2H), 1.99 (brs, 1H).

^13^C NMR (151 MHz, DMSO-d_6_) *δ* 160.67 (d, *J* = 240.8 Hz), 156.50 (d, *J* = 232.6 Hz), 141.45, 137.15, 136.64 (d, *J* = 3.2 Hz), 130.37 (d, *J* = 8.0 Hz), 126.10, 122.40 (d, *J* = 9.9 Hz), 121.62 (d, *J* = 4.1 Hz), 120.57, 118.56, 114.84 (d, *J* = 20.8 Hz), 113.02 (d, *J* = 25.4 Hz), 110.72 (d, *J* = 9.2 Hz), 110.01, 105.67 (d, *J* = 23.7 Hz), 68.84, 52.93, 51.19, 47.17, 35.08.

##### 2.2.7.3. 4-(2-((3-(3-Fluoro-9H-carbazol-9-yl)-2-hydroxypropyl)amino)ethyl)phenol (WK-3)

White solid, 119.0 mg (yield 48%). IR (KBr), ν, cm^−1^: 2932, 1597, 1518, 1488, 1465, 1465, 1271, 1245, 1169, 888, 739.

m.p. 140–142 °C. HRMS-ESI calcd. for C_23_H_24_FN_2_O_2_ [M + H]^+^ 379.1822, found 379.1802.

^1^H NMR (600 MHz, DMSO-d_6_) *δ* 9.14 (brs, 1H), 8.14 (d, *J* = 7.7 Hz, 1H), 7.97 (dd, *J* = 9.2, 2.6 Hz, 1H), 7.60–7.58 (m, 2H), 7.44 (t, *J* = 7.7 Hz, 1H), 7.26 (td, *J* = 9.1, 2.7 Hz, 1H), 7.17 (t, *J* = 7.4 Hz, 1H), 7.00 (d, *J* = 8.3 Hz, 2H), 6.67 (d, *J* = 8.4 Hz, 2H), 4.98 (brs, 1H), 4.44 (dd, *J* = 14.8, 5.0 Hz, 1H), 4.25 (dd, *J* = 14.8, 6.8 Hz, 1H), 3.96 (p, *J* = 5.6 Hz, 1H), 2.72–2.63 (m, 2H), 2.63–2.57 (m, 2H), 2.53 (dd, *J* = 11.9, 6.1 Hz, 1H), 1.88 (s, 1H).

^13^C NMR (151 MHz, DMSO-d_6_) *δ* 156.96 (d, *J* = 232.7 Hz), 155.90, 141.91, 137.61, 130.83, 129.91, 126.57, 122.86 (d, *J* = 9.9 Hz), 122.08 (d, *J* = 4.0 Hz), 121.02, 119.01, 115.47, 113.49 (d, *J* = 25.1 Hz), 111.20 (d, *J* = 8.9 Hz), 110.49, 106.12 (d, *J* = 23.7 Hz), 69.25, 53.38, 51.97, 47.64, 35.54.

##### 2.2.7.4. 1-(3-fluoro-9H-carbazol-9-yl)-3-((2-(2-methoxyphenoxy) ethyl)amino) propan-2-ol (WK-4)

White solid, 69.0 mg (yield 41%). IR (KBr), ν, cm^−1^: 3352, 1591, 1507, 1490, 1467, 1255, 1221, 1124, 1017, 745.

m.p. 55–57 °C. HRMS-ESI calcd. for C_24_H_26_FN_2_O_3_ [M + H]^+^ 409.1927, found 409.1901.

^1^H NMR (600 MHz, DMSO-d_6_) *δ* 8.15 (d, *J* = 7.7 Hz, 1H), 7.98 (dd, *J* = 9.2, 2.7 Hz, 1H), 7.67–7.61 (m, 2H), 7.44 (t, *J* = 8.1 Hz, 1H), 7.27 (td, *J* = 9.1, 2.7 Hz, 1H), 7.18 (t, *J* = 7.4 Hz, 1H), 6.99–6.93 (m, 2H), 6.92–6.84 (m, 2H), 5.13 (s, 1H), 4.47 (dd, *J* = 14.8, 5.1 Hz, 1H), 4.29 (dd, *J* = 14.8, 6.9 Hz, 1H), 4.02 (hept, *J* = 5.5, 5.0 Hz, 3H), 3.74 (s, 3H), 3.43–3.34 (m, 1H), 2.88 (t, *J* = 5.6 Hz, 2H), 2.68 (dd, *J* = 11.8, 4.7 Hz, 1H), 2.60 (dd, *J* = 11.8, 6.3 Hz, 1H).

^13^C NMR (151 MHz, DMSO-d_6_) *δ* 156.52 (d, *J* = 232.4 Hz), 149.17, 148.06, 141.46, 137.16, 126.14, 122.42 (d, *J* = 9.9 Hz), 121.65 (d, *J* = 4.1 Hz), 121.05, 120.72, 120.58, 118.57, 113.61, 113.06 (d, *J* = 25.4 Hz), 112.20, 110.76 (d, *J* = 9.2 Hz), 110.02, 105.67 (d, *J* = 23.7 Hz), 68.82, 68.22, 55.45, 52.90, 48.45, 47.14.

##### 2.2.7.5. 1-((3-Butoxypropyl)amino)-3-(3-fluoro-9H-carbazol-9-yl)propan-2-ol (WK-5)

White solid, 91.0 mg (yield 59%). IR (KBr), ν, cm^−1^: 3257, 2931, 2862, 1575, 1487, 1466, 1281, 1167, 1111, 887, 738.

m.p. 71–73 °C. HRMS-ESI calcd. for C_22_H_30_FN_2_O_2_ [M + H]^+^ 373.2291, found 373.2270.

^1^H NMR (600 MHz, DMSO-d_6_) *δ* 8.15 (d, *J* = 7.7 Hz, 1H), 7.98 (dd, *J* = 9.2, 2.6 Hz, 1H), 7.66–7.60 (m, 2H), 7.45 (t, *J* = 8.3 Hz, 1H), 7.28 (td, *J* = 9.2, 2.6 Hz, 1H), 7.18 (t, *J* = 7.4 Hz, 1H), 5.04 (s, 1H), 4.45 (dd, *J* = 14.8, 4.9 Hz, 1H), 4.27 (dd, *J* = 14.9, 6.9 Hz, 1H), 3.96 (p, *J* = 5.6 Hz, 1H), 3.40 (t, *J* = 6.4 Hz, 2H), 3.33 (t, *J* = 6.5 Hz, 2H), 2.59–2.51 (m, 4H), 1.86 (s, 1H), 1.64 (p, *J* = 6.6 Hz, 2H), 1.50–1.41 (m, 2H), 1.30 (h, *J* = 7.4 Hz, 2H), 0.86 (s, 3H).

^13^C NMR (151 MHz, DMSO-d_6_) *δ* 156.51 (d, *J* = 232.7 Hz), 141.48, 137.18, 126.09, 122.40 (d, *J* = 9.9 Hz), 121.64 (d, *J* = 3.9 Hz), 120.57, 118.55, 113.01 (d, *J* = 25.3 Hz), 110.75 (d, *J* = 9.1 Hz), 110.02, 105.67 (d, *J* = 23.7 Hz), 69.67, 68.83, 68.41, 53.25, 47.26, 46.72, 31.37, 29.79, 18.91, 13.79.

##### 2.2.7.6. 1-(3-Fluoro-9H-carbazol-9-yl)-3-((2-hydroxypropyl)amino)propan-2-ol (WK-6)

White solid, 74.0 mg (yield 56%). IR (KBr), ν, cm^−1^: 3305, 1586, 1488, 1463, 1170, 950, 791, 743.

m.p. 118–120 °C. HRMS-ESI calcd. for C_18_H_22_FN_2_O_2_ [M + H]^+^ 317.1665, found 317.1649.

^1^H NMR (600 MHz, DMSO-d_6_) *δ* 8.16 (d, *J* = 7.8 Hz, 1H), 7.99 (dd, *J* = 9.2, 2.7 Hz, 1H), 7.70–7.63 (m, 2H), 7.47 (t, *J* = 7.5 Hz, 1H), 7.30 (td, *J* = 9.2, 2.7 Hz, 1H), 7.19 (t, *J* = 7.4 Hz, 1H), 5.39 (s, 1H), 4.82 (s, 1H), 4.46 (dd, *J* = 14.9, 5.1 Hz, 1H), 4.34 (dd, *J* = 14.8, 6.9 Hz, 1H), 4.13 (q, *J* = 4.6 Hz, 1H), 3.85–3.77 (m, 1H), 2.79 (dd, *J* = 12.1, 4.0 Hz, 1H), 2.71 (dd, *J* = 12.1, 7.3 Hz, 1H), 2.62 (dd, *J* = 12.0, 3.9 Hz, 1H), 2.57–2.51 (m, 1H), 1.06 (d, *J* = 6.2 Hz, 3H).

^13^C NMR (151 MHz, DMSO-d_6_) *δ* 157.02 (d, *J* = 232.7 Hz), 141.89, 137.58, 126.62, 122.94 (d, *J* = 9.8 Hz), 122.15 (d, *J* = 4.1 Hz), 121.07, 119.12, 113.54 (d, *J* = 25.4 Hz), 111.25 (d, *J* = 9.1 Hz), 110.51, 106.18 (d, *J* = 23.6 Hz), 68.08, 64.54, 56.59, 52.61, 47.50, 21.86.

##### 2.2.7.7. 2-((3-(3-Fluoro-9H-carbazol-9-yl)-2-hydroxypropyl)amino)propan-1-ol (WK-7)

White solid, 100.0 mg (yield 76%). IR (KBr), ν, cm^−1^: 3415, 2976, 1487, 1468, 1283, 1168, 1052, 885, 856, 742.

m.p. 119–121 °C. HRMS-ESI calcd. for C_18_H_22_FN_2_O_2_ [M + H]^+^ 317.1665, found 317.1650.

^1^H NMR (600 MHz, DMSO-d_6_) *δ* 8.15 (d, *J* = 7.5 Hz, 1H), 7.98 (dd, *J* = 9.3, 2.6 Hz, 1H), 7.67–7.61 (m, 2H), 7.45 (ddd, *J* = 8.3, 7.0, 1.2 Hz, 1H), 7.29 (td, *J* = 9.1, 2.6 Hz, 1H), 7.18 (t, *J* = 7.4 Hz, 1H), 5.07 (d, *J* = 5.1 Hz, 1H), 4.57 (t, *J* = 5.4 Hz, 1H), 4.44 (dd, *J* = 14.9, 4.8 Hz, 1H), 4.28 (dd, *J* = 14.9, 7.0 Hz, 1H), 3.99–3.89 (m, 1H), 3.30 (dt, *J* = 9.6, 4.5 Hz, 1H), 3.25–3.18 (m, 1H), 2.67 (dd, *J* = 11.4, 4.7 Hz, 1H), 2.56 (dt, *J* = 11.5, 6.3 Hz, 1H), 2.48 (d, *J* = 7.1 Hz, 1H), 1.91 (s, 1H), 0.87 (d, *J* = 6.3 Hz, 3H).

^13^C NMR (151 MHz, DMSO-d_6_) *δ* 156.96 (d, *J* = 232.3 Hz), 141.94, 137.64, 126.55, 122.86 (d, *J* = 9.6 Hz), 122.09 (d, *J* = 4.0 Hz), 121.02, 119.01, 113.47 (d, *J* = 25.1 Hz), 111.29 (d, *J* = 9.2 Hz), 110.56, 106.12 (d, *J* = 23.8 Hz), 69.89, 65.95, 55.37, 51.39, 47.95, 17.74.

##### 2.2.7.8. 1-(3-Fluoro-9H-carbazol-9-yl)-3-(((R)-2-phenylpropyl)amino)propan-2-ol (WK-8)

White solid, 83.0 mg (yield 53%). IR (KBr), ν, cm^−1^: 2960, 1589, 1486, 1462, 1279, 1162, 1099, 884, 793, 700.

m.p. 100–102 °C. HRMS-ESI calcd. for C_24_H_26_FN_2_O [M + H]^+^ 377.2029, found 377.2006.

^1^H NMR (600 MHz, DMSO-d_6_) *δ* 8.14 (d, *J* = 7.6 Hz, 1H), 7.97 (dd, *J* = 9.3, 2.6 Hz, 1H), 7.61–7.53 (m, 2H), 7.47–7.41 (m, 1H), 7.31–7.21 (m, 5H), 7.20–7.15 (m, 2H), 5.01 (s, 1H), 4.42 (dd, *J* = 14.8, 5.1 Hz, 1H), 4.23 (dd, *J* = 14.8, 6.7 Hz, 1H), 3.93 (p, *J* = 5.7 Hz, 1H), 2.87 (h, *J* = 7.0 Hz, 1H), 2.68 (dd, *J* = 11.5, 7.1 Hz, 1H), 2.62 (dd, *J* = 11.5, 7.1 Hz, 1H), 2.56 (dd, *J* = 11.9, 5.1 Hz, 1H), 2.51 (d, *J* = 6.7 Hz, 1H), 1.82 (s, 1H), 1.22 (d, *J* = 7.0 Hz, 3H).

^13^C NMR (151 MHz, DMSO-d_6_) *δ* 156.96 (d, *J* = 232.7 Hz), 146.26, 141.90, 137.59, 128.73, 127.55, 126.57, 126.45, 122.85 (d, *J* = 9.5 Hz), 122.07 (d, *J* = 4.0 Hz), 121.02, 119.01, 113.48 (d, *J* = 25.4 Hz), 111.17 (d, *J* = 9.1 Hz), 110.46, 106.12 (d, *J* = 23.7 Hz), 69.18, 57.33, 53.43, 47.55, 39.73, 20.25.

##### 2.2.7.9. 1-(3-Fluoro-9H-carbazol-9-yl)-3-((4-fluorobenzyl)amino)propan-2-ol (WK-9)

White solid, 84.0 mg (yield 55%). IR (KBr), ν, cm^−1^: 2924, 1606, 1587, 1516, 1487, 1223, 1171, 889, 790, 740.

m.p. 130–132 °C. HRMS-ESI calcd. for C_22_H_21_F_2_N_2_O [M + H]^+^ 367.1622, found 367.1608.

^1^H NMR (600 MHz, DMSO-d_6_) *δ* 8.14 (d, *J* = 7.8 Hz, 1H), 7.98 (dd, *J* = 9.3, 2.6 Hz, 1H), 7.61 (dd, *J* = 8.7, 4.3 Hz, 2H), 7.48–7.41 (m, 1H), 7.36 (dd, *J* = 8.5, 5.8 Hz, 2H), 7.28 (td, *J* = 9.1, 2.7 Hz, 1H), 7.18 (t, *J* = 7.4 Hz, 1H), 7.12 (t, *J* = 8.9 Hz, 2H), 5.10–4.96 (m, 1H), 4.47 (dd, *J* = 14.9, 4.6 Hz, 1H), 4.28 (dd, *J* = 14.9, 7.1 Hz, 1H), 3.99 (s, 1H), 3.74–3.61 (m, 2H), 2.60–2.52 (m, 2H), 2.38 (brs, 1H).

^13^C NMR (151 MHz, DMSO-d_6_) *δ* 161.53 (d, *J* = 241.6 Hz), 156.96 (d, *J* = 232.7 Hz), 141.93, 137.63, 137.41 (d, *J* = 2.8 Hz), 130.29 (d, *J* = 7.9 Hz), 126.55, 122.86 (d, *J* = 9.9 Hz), 122.10 (d, *J* = 4.1 Hz), 121.02, 119.01, 115.26 (d, *J* = 21.0 Hz), 113.47 (d, *J* = 25.2 Hz), 111.23 (d, *J* = 9.1 Hz), 110.51, 106.12 (d, *J* = 23.7 Hz), 69.37, 53.08, 52.82, 47.81.

##### 2.2.7.10. (2S)-tert-butyl 4-(3-(3-fluoro-9H-carbazol-9-yl)-2-hydroxypropyl)-2-(hydroxymethyl)piperazine-1-carboxylate (WK-10)

A mixture of intermediate compound **a4** (200.0 mg, 0.8 mmol, 1 equiv.) and **e1** (367 mg, 1.7 mmol, 2.1 equiv.) dissolved in 5 ml ethanol (EtOH), was introduced into a 10 ml sealed tube. The mixture was stirred at 60 °C and monitored by TLC until compound **a4** was completely consumed. The mixture was treated with EtOAc and water. The organic layer was washed with saturated NaCl and it was dried over anhydrous Na_2_SO_4_ and concentrated *in vacuo*. Final flash column chromatography utilising dichloromethane/methanol (DCM/MeOH) (20:1) as the eluent afforded *(2S)-tert-butyl 4-(3-(3-fluoro-9H-carbazol-9-yl)-2-hydroxypropyl)-2-(hydroxymethyl)piperazine-1-carboxylate* (**WK-10**). White solid, 111.0 mg (yield 29%).

IR (KBr), ν, cm^−1^: 3387, 1667, 1487, 1465, 1416, 1281, 1166, 1129, 859, 799, 721.

m.p. 71–73 °C. HRMS-ESI calcd. for C_25_H_33_FN_3_O_4_ [M + H]^+^ 458.2455, found 458.2461.

^1^H NMR (600 MHz, DMSO-d_6_) *δ* 8.14 (t, *J* = 6.8 Hz, 1H), 7.98 (ddd, *J* = 9.2, 6.6, 2.6 Hz, 1H), 7.70–7.58 (m, 2H), 7.45 (dt, *J* = 16.1, 7.7 Hz, 1H), 7.28 (dtd, *J* = 20.9, 9.2, 2.6 Hz, 1H), 7.18 (td, *J* = 7.4, 4.8 Hz, 1H), 5.13–4.60 (m, 2H), 4.49 (dd, *J* = 14.9, 3.0 Hz, 1H), 4.34–4.23 (m, 1H), 3.99 (dd, *J* = 77.4, 27.9 Hz, 2H), 3.84–3.67 (m, 2H), 3.47 (s, 1H), 3.23–2.93 (m, 2H), 2.78 (dd, *J* = 72.6, 7.9 Hz, 1H), 2.47–2.29 (m, 2H), 2.09–1.90 (m, 2H), 1.40 (s, 9H).

^13^C NMR (151 MHz, DMSO-d_6_) *δ* 156.52 (d, *J* = 232.3 Hz), 141.71, 137.40, 126.09, 122.45 (d, *J* = 9.7 Hz), 121.68 (d, *J* = 4.2 Hz), 120.51, 118.53, 112.99 (d, *J* = 24.7 Hz), 111.06 (d, *J* = 9.1 Hz), 110.34, 105.60 (d, *J* = 23.7 Hz), 78.75, 67.63, 66.69, 62.10, 53.88, 53.25, 53.04, 52.18, 47.69, 28.10.

##### 2.2.7.11. 1-(3-Fluoro-9H-carbazol-9-yl)-3-((S)-3-(hydroxymethyl)piperazin-1-yl)propan-2-ol (WK-11)

A mixture of **WK-10** (201.0 mg, 0.4 mmol, 1 equiv.) and 2 N hydrogen chloride-1, 4-Dioxane solution 5 ml was introduced into a 10 ml sealed tube. The mixture was stirred at room temperature for 2 h. After **WK-10** was completely consumed, the mixture was treated with EtOAc and water. The organic layer was combined, washed with saturated NaCl, dried over anhydrous Na_2_SO_4_, and concentrated *in vacuo*. Final flash column chromatography utilising DCM/MeOH (20:1) as the eluent afforded *1-(3-fluoro-9H-carbazol-9-yl)-3-((S)-3-(hydroxymethyl)piperazin-1-yl)propan-2-ol* (**WK-11**). White solid, 120.0 mg (yield 77%).

IR (KBr), ν, cm^−1^: 3311, 2925, 1629, 1585, 1486, 1463, 1322, 1282, 886, 799, 721.

m.p. 35–37 °C. HRMS-ESI calcd. For C_20_H_25_FN_3_O_2_ [M + H]^+^ 358.1931, found 358.1950.

^1^H NMR (600 MHz, DMSO-d_6_) *δ* 8.14 (d, *J* = 7.7 Hz, 1H), 7.98 (dd, *J* = 9.2, 2.5 Hz, 1H), 7.69–7.57 (m, 2H), 7.45 (t, *J* = 7.7 Hz, 1H), 7.29 (td, *J* = 9.2, 2.6 Hz, 1H), 7.18 (t, *J* = 7.4 Hz, 1H), 5.31 (s, 1H), 5.12 (s, 1H), 4.45 (dt, *J* = 14.8, 4.5 Hz, 1H), 4.31 (dd, *J* = 14.9, 6.7 Hz, 1H), 4.07 (s, 1H), 3.53 (s, 2H), 3.47 (dt, *J* = 18.3, 5.6 Hz, 1H), 3.13–3.02 (m, 2H), 2.95–2.77 (m, 3H), 2.47–2.34 (m, 2H), 2.30 (q, *J* = 11.0, 10.0 Hz, 1H), 2.16 (q, *J* = 12.5, 12.1 Hz, 1H).

^13^C NMR (151 MHz, DMSO-d_6_) *δ* 156.54 (d, *J* = 232.6 Hz), 141.53, 137.23, 126.13, 122.46 (d, *J* = 9.9 Hz), 121.69 (d, *J* = 4.1 Hz), 120.60, 118.62, 113.06 (d, *J* = 25.3 Hz), 110.98 (d, *J* = 9.0 Hz), 110.22, 105.70 (d, *J* = 23.7 Hz), 67.16, 61.36, 59.81, 56.09, 54.99, 50.29, 47.53, 42.99.

##### 2.2.7.12. 1-((2-(1H-indol-3-yl)ethyl)amino)-3-(3,6-difluoro-9H-carbazol-9-yl)propan-2-ol (WK-12)

A mixture of intermediate compound **b4** (100.0 mg, 0.4 mmol, 1 equiv.) and tryptamine (247.0 mg, 1.5 mmol, 3.75 equiv.) dissolved in 5 ml EtOH, was introduced into a 10 ml sealed tube. The mixture was stirred at 60 °C and monitored by TLC until compound **b4** was completely consumed. The mixture was treated with EtOAc and water. The organic layer was washed with saturated aqueous NaCl, dried over anhydrous Na_2_SO_4_, and concentrated *in vacuo*. Final flash column chromatography utilising PE/EA (1:2) as the eluent afforded *1-((2-(1H-indol-3-yl)ethyl)amino)-3-(3,6-difluoro-9H-carbazol-9-yl)propan-2-ol* (**WK-12**). Off-white solid, 63.0 mg (yield 39%). **WK-13**–**WK-19** were prepared in a procedure similar to that described for **WK-12**.

IR (KBr), ν, cm^−1^: 3285, 1735, 1492, 1470, 1146, 1124, 854, 797, 787, 732.

m.p. 130–132 °C. HRMS-ESI calcd. for C_25_H_24_F_2_N_3_O [M + H]^+^ 420.1887, found 420.1867.

^1^H NMR (600 MHz, DMSO-d_6_) *δ* 10.78 (s, 1H), 7.99 (dd, *J* = 9.2, 2.7 Hz, 2H), 7.59 (dd, *J* = 9.0, 4.3 Hz, 2H), 7.52 (d, *J* = 7.9 Hz, 1H), 7.33 (d, *J* = 8.1 Hz, 1H), 7.29–7.22 (m, 2H), 7.15 (s, 1H), 7.06 (t, *J* = 7.5 Hz, 1H), 6.97 (t, *J* = 7.4 Hz, 1H), 5.01 (s, 1H), 4.45 (dd, *J* = 14.9, 4.8 Hz, 1H), 4.27 (dd, *J* = 14.9, 6.9 Hz, 1H), 3.95 (s, 1H), 2.90–2.75 (m, 4H), 2.60 (ddd, *J* = 46.7, 11.8, 5.6 Hz, 2H), 1.88 (brs, 1H).

^13^C NMR (151 MHz, DMSO-d_6_) *δ* 156.27 (d, *J* = 232.7 Hz), 138.04, 136.24, 127.29, 122.60, 121.89 (dd, *J* = 10.0, 4.2 Hz), 120.79, 118.31, 118.10, 113.78 (d, *J* = 25.4 Hz), 112.62, 111.32, 111.13 (d, *J* = 9.0 Hz), 105.99 (d, *J* = 23.8 Hz), 68.94, 52.92, 50.36, 47.35, 25.58.

##### 2.2.7.13. 4-(2-((3-(3,6-Difluoro-9H-carbazol-9-yl)-2-hydroxypropyl) amino) ethyl) phenol (WK-13)

White solid, 51.0 mg (yield 33%). IR (KBr), ν, cm^−1^: 2934, 1586, 1494, 1471, 1269, 1247, 1189, 1148, 849, 788.

m.p. 143–145 °C. HRMS-ESI calcd. for C_23_H_23_F_2_N_2_O_2_ [M + H]^+^ 397.1728, found 397.1709.

^1^H NMR (600 MHz, DMSO-d_6_) *δ* 9.14 (s, 1H), 7.99 (dd, *J* = 9.2, 2.7 Hz, 2H), 7.59 (dd, *J* = 9.0, 4.3 Hz, 2H), 7.29 (td, *J* = 9.1, 2.7 Hz, 2H), 7.00 (d, *J* = 7.9 Hz, 2H), 6.67 (d, *J* = 7.9 Hz, 2H), 5.01 (s, 1H), 4.43 (dd, *J* = 14.9, 4.8 Hz, 1H), 4.25 (dd, *J* = 14.8, 6.9 Hz, 1H), 3.93 (t, *J* = 5.8 Hz, 1H), 2.72–2.62 (m, 2H), 2.63–2.56 (m, 3H), 2.53 (d, *J* = 6.2 Hz, 1H), 1.96 (s, 1H).

^13^C NMR (151 MHz, DMSO-d_6_) *δ* 156.28 (d, *J* = 232.9 Hz), 155.41, 138.03, 130.44, 129.44, 121.90 (dd, *J* = 9.8, 4.2 Hz), 114.99, 113.80 (d, *J* = 25.5 Hz), 111.14 (d, *J* = 9.0 Hz), 106.00 (d, *J* = 23.8 Hz), 68.89, 52.89, 51.54, 47.35, 35.14.

##### 2.2.7.14. 1-(3,6-Difluoro-9H-carbazol-9-yl)-3-((4-fluorophenethyl)amino)propan-2-ol (WK-14)

White solid, 36.0 mg (yield 23%). IR (KBr), ν, cm^−1^: 2819, 1511, 1491, 1470, 1145, 938, 825, 788.

m.p. 114–116 °C. HRMS-ESI calcd. for C_23_H_22_F_3_N_2_O [M + H]^+^ 399.1684, found 399.1658.

^1^H NMR (600 MHz, DMSO-d_6_) *δ* 8.00 (dd, *J* = 9.2, 2.6 Hz, 2H), 7.58 (dd, *J* = 9.0, 4.3 Hz, 2H), 7.32–7.24 (m, 4H), 7.10 (t, *J* = 8.9 Hz, 2H), 5.03 (s, 1H), 4.43 (dd, *J* = 14.9, 4.8 Hz, 1H), 4.25 (dd, *J* = 14.9, 6.9 Hz, 1H), 3.93 (t, *J* = 5.9 Hz, 1H), 2.76–2.68 (m, 4H), 2.60 (dd, *J* = 11.8, 5.2 Hz, 1H), 2.55–2.52 (m, 1H), 1.92 (s, 1H).

^13^C NMR (151 MHz, DMSO-d_6_) *δ* 161.10 (d, *J* = 241.1 Hz), 156.72 (d, *J* = 232.8 Hz), 138.46, 137.08 (d, *J* = 2.9 Hz), 130.80 (d, *J* = 7.8 Hz), 122.34 (dd, *J* = 10.0, 4.3 Hz), 115.26 (d, *J* = 21.0 Hz), 114.23 (d, *J* = 25.4 Hz), 111.55 (d, *J* = 9.0 Hz), 106.46 (d, *J* = 23.8 Hz), 69.33, 53.30, 51.62, 47.78, 35.52.

##### 2.2.7.15. 1-(3,6-Difluoro-9H-carbazol-9-yl)-3-(phenethylamino)propan-2-ol (WK-15)

White solid, 75.0 mg (yield 51%). IR (KBr), ν, cm^−1^: 2846, 1585, 1491, 1478, 1298, 1181, 1142, 936, 855, 784.

m.p. 91–93 °C. HRMS-ESI calcd. for C_23_H_23_F_2_N_2_O [M + H]^+^ 381.1778, found 381.1757.

^1^H NMR (600 MHz, DMSO-d_6_) *δ* 8.00 (dd, *J* = 9.2, 2.6 Hz, 2H), 7.59 (dd, *J* = 9.0, 4.3 Hz, 2H), 7.33–7.26 (m, 4H), 7.25–7.20 (m, 2H), 7.18 (t, *J* = 7.3 Hz, 1H), 5.03 (s, 1H), 4.43 (dd, *J* = 14.9, 4.8 Hz, 1H), 4.26 (dd, *J* = 14.9, 6.9 Hz, 1H), 3.94 (s, 1H), 2.78–2.67 (m, 4H), 2.60 (dd, *J* = 11.8, 5.2 Hz, 1H), 2.53 (dd, *J* = 11.8, 6.0 Hz, 1H), 1.87 (s, 1H).

^13^C NMR (151 MHz, DMSO-d_6_) *δ* 156.27 (d, *J* = 232.8 Hz), 140.48, 138.02, 128.41 (d, *J* = 65.3 Hz), 125.80, 121.89 (dd, *J* = 10.0, 4.3 Hz), 113.79 (d, *J* = 25.4 Hz), 111.12 (d, *J* = 9.1 Hz), 106.01 (d, *J* = 23.9 Hz), 68.89, 52.87, 51.22, 47.33, 36.02.

##### 2.2.7.16. 1-(3,6-Difluoro-9H-carbazol-9-yl)-3-((2-(2-methoxyphenoxy) ethyl) amino) propan-2-ol (WK-16)

White solid, 77.0 mg (yield 47%). IR (KBr), ν, cm^−1^: 3375, 1586, 1508, 1493, 1471, 1255, 1221, 1126, 865, 747.

m.p. 93–95 °C. HRMS-ESI calcd. for C_24_H_25_F_2_N_2_O_3_ [M + H]^+^ 427.1833, found 427.1815.

^1^H NMR (600 MHz, DMSO-d_6_) *δ* 8.00 (dd, *J* = 9.2, 2.6 Hz, 2H), 7.65 (dd, *J* = 9.0, 4.3 Hz, 2H), 7.30 (td, *J* = 9.2, 2.6 Hz, 2H), 6.96 (td, *J* = 7.4, 1.9 Hz, 2H), 6.88 (dtd, *J* = 17.0, 7.4, 1.8 Hz, 2H), 5.10 (s, 1H), 4.46 (dd, *J* = 14.9, 4.9 Hz, 1H), 4.29 (dd, *J* = 14.9, 6.9 Hz, 1H), 4.06–3.96 (m, 3H), 3.73 (s, 3H), 2.87 (t, *J* = 5.4 Hz, 2H), 2.66 (dd, *J* = 11.8, 4.9 Hz, 1H), 2.58 (dd, *J* = 11.8, 6.2 Hz, 1H), 2.23 (s, 1H).

^13^C NMR (151 MHz, DMSO-d_6_) *δ* 156.77 (d, *J* = 232.7 Hz), 149.61, 148.54, 138.51, 122.39 (dd, *J* = 10.0, 4.3 Hz), 121.48, 121.17, 114.30 (d, *J* = 25.4 Hz), 114.02, 112.65, 111.63 (d, *J* = 9.0 Hz), 106.50 (d, *J* = 23.8 Hz), 69.38, 68.73, 55.90, 53.33, 48.95, 47.76.

##### 2.2.7.17. 1-((3-butoxypropyl)amino)-3-(3,6-difluoro-9H-carbazol-9-yl)propan-2-ol (WK-17)

White solid, 52.0 mg (yield 34%). IR (KBr), ν, cm^−1^: 3261, 2931, 2863, 2820, 1586, 1492, 1475, 1146, 939, 844, 789.

m.p. 94–96 °C. HRMS-ESI calcd. for C_22_H_29_F_2_N_2_O_2_ [M + H]^+^ 391.2197, found 391.2179.

^1^H NMR (600 MHz, DMSO-d_6_) *δ* 8.00 (dd, *J* = 9.2, 2.6 Hz, 2H), 7.64 (dd, *J* = 9.0, 4.3 Hz, 2H), 7.31 (td, *J* = 9.1, 2.6 Hz, 2H), 5.02 (s, 1H), 4.45 (dd, *J* = 14.9, 4.7 Hz, 1H), 4.27 (dd, *J* = 14.9, 6.9 Hz, 1H), 3.94 (p, *J* = 5.7 Hz, 1H), 3.40 (t, *J* = 6.4 Hz, 2H), 3.33 (t, *J* = 6.5 Hz, 3H), 2.59–2.51 (m, 3H), 1.93 (s, 1H), 1.64 (p, *J* = 6.6 Hz, 2H), 1.51–1.41 (m, 2H), 1.30 (h, *J* = 7.4 Hz, 2H), 0.86 (t, *J* = 7.4 Hz, 3H).

^13^C NMR (151 MHz, DMSO-d_6_) *δ* 156.76 (d, *J* = 232.8 Hz), 138.53, 122.38 (dd, *J* = 10.0, 4.3 Hz), 114.26 (d, *J* = 25.4 Hz), 111.61 (d, *J* = 9.0 Hz), 106.50 (d, *J* = 23.9 Hz), 70.13, 69.39, 68.87, 53.68, 47.89, 47.19, 31.83, 30.29, 19.37, 14.25.

##### 2.2.7.18. 1-(3,6-Difluoro-9H-carbazol-9-yl)-3-((2-hydroxypropyl)amino)propan-2-ol (WK-18)

White solid, 80.0 mg (yield 62%). IR (KBr), ν, cm^−1^: 3300, 2968, 1585, 1494, 1478, 1147, 1099, 938, 865, 788.

m.p. 130–132 °C. HRMS-ESI calcd. for C_18_H_21_F_2_N_2_O_2_ [M + H]^+^ 335.1571, found 335.1554.

^1^H NMR (600 MHz, DMSO-d_6_) *δ* 8.00 (dd, *J* = 9.2, 2.7 Hz, 2H), 7.64 (dd, *J* = 9.0, 4.3 Hz, 2H), 7.32 (td, *J* = 9.1, 2.7 Hz, 2H), 5.05 (s, 1H), 4.52 (s, 1H), 4.45 (dd, *J* = 14.9, 4.9 Hz, 1H), 4.28 (dd, *J* = 14.8, 6.9 Hz, 1H), 3.95 (p, *J* = 5.5 Hz, 1H), 3.71 (h, *J* = 6.1 Hz, 1H), 2.57 (dd, *J* = 11.8, 4.9 Hz, 1H), 2.54–2.51 (m, 1H), 2.41 (d, *J* = 5.8 Hz, 2H), 2.05 (s, 1H), 1.05 (d, *J* = 6.2 Hz, 3H).

^13^C NMR (151 MHz, DMSO-d_6_) *δ* 156.76 (d, *J* = 232.8 Hz), 138.50, 122.38 (dd, *J* = 10.0, 4.3 Hz), 114.29 (d, *J* = 25.5 Hz), 111.61 (d, *J* = 8.9 Hz), 106.51 (d, *J* = 23.9 Hz), 69.29, 65.70, 57.89, 53.50, 47.76, 22.04.

##### 2.2.7.19. 2-((3-(3,6-Difluoro-9H-carbazol-9-yl)-2-hydroxypropyl)amino)propan-1-ol (WK-19)

White solid, 97.0 mg (yield 75%). IR (KBr), ν, cm^−1^: 2977, 1490, 1473, 1300, 1177, 1149, 1049, 846, 793, 786.

m.p. 136–138 °C. HRMS-ESI calcd. for C_18_H_21_F_2_N_2_O_2_ [M + H]^+^ 335.1571, found 335.1558.

^1^H NMR (600 MHz, DMSO-d_6_) *δ* 8.00 (dd, *J* = 9.2, 2.6 Hz, 2H), 7.65 (dd, *J* = 9.0, 4.3 Hz, 2H), 7.32 (td, *J* = 9.1, 2.7 Hz, 2H), 5.07 (s, 1H), 4.58 (s, 1H), 4.44 (dd, *J* = 14.9, 4.5 Hz, 1H), 4.28 (dd, *J* = 14.9, 7.1 Hz, 1H), 3.92 (s, 1H), 3.30 (dd, *J* = 10.7, 4.8 Hz, 1H), 3.22 (d, *J* = 7.4 Hz, 1H), 2.67 (dd, *J* = 11.4, 4.8 Hz, 1H), 2.55 (h, *J* = 6.3 Hz, 1H), 2.49–2.43 (m, 1H), 1.97 (s, 1H), 0.88 (d, *J* = 6.3 Hz, 3H).

^13^C NMR (151 MHz, DMSO-d_6_) *δ* 156.76 (d, *J* = 232.8 Hz), 138.52, 122.38 (dd, *J* = 9.9, 4.1 Hz), 114.26 (d, *J* = 25.4 Hz), 111.70 (d, *J* = 9.0 Hz), 106.49 (d, *J* = 23.8 Hz), 69.92, 65.91, 55.37, 51.29, 48.10, 17.71.

##### 2.2.7.20. General procedure for compound WK-20 and WK-21. 1-((2-(1H-indol-3-yl)ethyl)amino)-3-(9H-pyrido[3,4-b]indol-9-yl)propan-2-ol (WK-20)

Powder KOH was added to a 9*H*-*β*-carboline solution in *N*, *N*-dimethylformamide (DMF) at ambient temperature and stirred for 30 min until dissolved. Epichlorohydrin was added via syringe, and the mixture was stirred at room temperature. Upon completion, the solution was partitioned between EtOAc and H_2_O. The crude products were used for the next step without further purification. A mixture of crude products (200.0 mg) and tryptamine (aromatic amines) (284.0 mg, 1.8 mmol) dissolved in 5 ml EtOH, was introduced into a 10 ml sealed tube. The mixture was stirred at 60 °C. Upon completion, the mixture was treated with EtOAc and water. The organic layer was washed with saturated aqueous NaCl, dried over anhydrous Na_2_SO_4_, and concentrated *in vacuo*. Finally purified by preparative thin layer chromatography to afford *1-((2-(1H-indol-3-yl)ethyl)amino)-3-(9H-pyrido[3,4-b]indol-9-yl)propan-2-ol* (**WK-20**). Off-white solid, 44.0 mg (yield 27%).

IR (KBr), ν, cm^−1^: 3051, 2921, 1625, 1454, 1329, 1217, 1033, 742, 730.

m.p. 85–87 °C. HRMS-ESI calcd. for C_24_H_25_N_4_O [M + H]^+^ 385.2028, found 385.2025.

^1^H NMR (400 MHz, DMSO-d_6_) *δ* 10.80 (s, 1H), 9.07 (s, 1H), 8.36 (d, *J* = 5.2 Hz, 1H), 8.26 (d, *J* = 7.8 Hz, 1H), 8.12 (d, *J* = 5.2 Hz, 1H), 7.71 (d, *J* = 8.3 Hz, 1H), 7.62–7.47 (m, 2H), 7.33 (d, *J* = 8.1 Hz, 1H), 7.27 (t, *J* = 7.4 Hz, 1H), 7.15 (d, *J* = 2.2 Hz, 1H), 7.06 (t, *J* = 7.5 Hz, 1H), 6.97 (t, *J* = 7.4 Hz, 1H), 5.14 (s, 1H), 4.57 (dd, *J* = 14.8, 4.4 Hz, 1H), 4.41 (dd, *J* = 14.8, 7.1 Hz, 1H), 4.05 (t, *J* = 5.9 Hz, 1H), 2.87 (s, 4H), 2.78–2.62 (m, 2H).

^13^C NMR (151 MHz, DMSO) *δ* 138.71, 133.84, 128.52, 123.15, 122.19, 121.35, 119.82, 118.77, 118.66, 114.82, 111.84, 111.10, 68.98, 52.95, 50.38, 47.77, 26.81, 25.42.

##### 2.2.7.21. 1-((2-(1H-indol-3-yl)ethyl)amino)-3-(9H-pyrido[2,3-b]indol-9-yl)propan-2-ol (WK-21)

Off-white solid, 16.0 mg (yield 17%). IR (KBr), ν, cm^−1^: 3359, 3293, 2920, 1592, 1573, 1464, 1415, 1213, 772, 732.

m.p. 107–109 °C. HRMS-ESI calcd. for C_24_H_25_N_4_O [M + H]^+^ 385.2028, found 385.2020.

^1^H NMR (400 MHz, DMSO-d_6_) *δ* 10.88 (s, 1H), 8.56 (d, *J* = 7.6 Hz, 1H), 8.45 (d, *J* = 4.5 Hz, 1H), 8.21 (d, *J* = 7.7 Hz, 1H), 7.75 (d, *J* = 8.3 Hz, 1H), 7.52 (t, *J* = 8.5 Hz, 2H), 7.34 (d, *J* = 8.1 Hz, 1H), 7.31–7.21 (m, 2H), 7.16 (d, *J* = 2.2 Hz, 1H), 7.07 (t, *J* = 7.5 Hz, 1H), 6.97 (t, *J* = 7.4 Hz, 1H), 5.62 (s, 1H), 4.50 (d, *J* = 5.7 Hz, 2H), 4.33 (s, 1H), 2.95 (ddt, *J* = 27.8, 21.8, 9.8 Hz, 6H).

^13^C NMR (151 MHz, DMSO) *δ* 138.71, 133.84, 128.52, 123.15, 122.19, 121.35, 119.82, 118.77, 118.66, 114.82, 111.84, 111.10, 68.98, 52.95, 50.38, 47.77, 26.81, 25.42.

##### 2.2.7.22. 1-((1,3-Bis(3,6-difluoro-9H-carbazol-9-yl)propan-2-yl)oxy)-3-(isopropylamino)propan-2-ol (WK-22)

A mixture of intermediate compound **d5** (330.0 mg, 0.6 mmol, 1 equiv.) and tryptamine (aromatic amines) (375.0 mg, 6.4 mmol, 10.7 equiv.) dissolved in 5 ml isopropyl alcohol, was introduced into a 10 ml sealed tube. The mixture was stirred at 60 °C and monitored by TLC until compound **d5** was completely consumed. The mixture was treated with EtOAc and H_2_O. The organic layer was washed with saturated aqueous NaCl, dried over anhydrous Na_2_SO_4_, and concentrated *in vacuo*. Final flash column chromatography utilising EA/MeOH (20:1) as the eluent afforded *1-((1, 3-bis(3,6-difluoro-9H-carbazol-9-yl)propan-2-yl)oxy)-3-(isopropylamino)propan-2-ol* (**WK-22**). White solid, 78.0 mg (yield 21%).

IR (KBr), ν, cm^−1^: 2982, 1622, 1547, 1489, 1298, 1181, 1169, 1145, 850, 790.

m.p. 89–91 °C. HRMS-ESI calcd. for C_33_H_32_F_4_N_3_O_2_ [M + H]^+^ 578.2431, found 578.2413.

^1^H NMR (600 MHz, DMSO-d_6_) *δ* 8.11–7.96 (m, 4H), 7.71 (ddd, *J* = 19.5, 9.0, 4.2 Hz, 4H), 7.40–7.27 (m, 4H), 4.68–4.58 (m, 4H), 4.20 (td, *J* = 7.6, 4.0 Hz, 1H), 2.71 (dp, *J* = 8.0, 3.8 Hz, 1H), 2.39 (dd, *J* = 9.4, 4.5 Hz, 1H), 2.24 (q, *J* = 7.4, 5.9 Hz, 2H), 1.85 (s, 2H), 1.71 (dd, *J* = 12.0, 3.4 Hz, 1H), 1.35 (dd, *J* = 11.9, 8.2 Hz, 1H), 0.73 (dd, *J* = 27.3, 6.3 Hz, 6H).

^13^C NMR (151 MHz, DMSO-d_6_) *δ* 172.71, 157.22, 155.68, 137.82 (d, *J* = 6.1 Hz), 122.12 (dd, *J* = 6.5, 3.5 Hz), 114.00 (d, *J* = 25.4 Hz), 111.12 (d, *J* = 9.0 Hz), 106.27 (d, *J* = 23.5 Hz), 77.75, 73.83, 67.16, 48.84, 48.08, 45.39, 45.35, 21.83, 21.74, 21.42.

##### 2.2.7.23. General methods for preparation of target compounds (WK-23–WK-25). 1-((1,3-bis(3-fluoro-9H-carbazol-9-yl)propan-2-yl)oxy)-3-(isopropylamino)propan-2-ol (WK-23)

A mixture of intermediate compound **d4** (460.0 mg, 1.0 mmol, 1 equiv.) and tryptamine (590.0 mg, 10.0 mmol, 10 equiv.) dissolved in 5 ml isopropyl alcohol, was introduced into a 10 ml sealed tube. The mixture was stirred at 60 °C and monitored by TLC until compound **d4** was completely consumed. The mixture was treated with EtOAc and H_2_O. The organic layer was washed with saturated aqueous NaCl, dried over anhydrous Na_2_SO_4_, and concentrated *in vacuo*. Final flash column chromatography utilising EA/MeOH (20:1) as the eluent afforded *1-((1,3-bis(3-fluoro-9H-carbazol-9-yl)propan-2-yl)oxy)-3-(isopropylamino)propan-2-ol* (**WK-23**). White solid, 86.0 mg (yield 17%).

White solid, 86.0 mg (yield 17%). IR (KBr), ν, cm^−1^: 3325, 2978, 1588, 1490, 1469, 1286, 1169, 1052, 879, 741.

m.p. 195–196 °C. HRMS-ESI calcd. for C_33_H_34_F_2_N_3_O_2_ [M + H]^+^ 541.2619, found 542.2587.

^1^H NMR (600 MHz, DMSO-d_6_) *δ* 8.18 (d, *J* = 7.7 Hz, 2H), 8.11 (brs, 1H), 8.02 (dt, *J* = 9.2, 2.8 Hz, 2H), 7.74–7.64 (m, 4H), 7.48 (qd, *J* = 7.2, 1.2 Hz, 2H), 7.36–7.29 (m, 2H), 7.21 (td, *J* = 7.4, 4.2 Hz, 2H), 5.00 (brs, 1H), 4.77–4.58 (m, 4H), 4.27 (tt, *J* = 8.2, 4.2 Hz, 1H), 3.18 (tt, *J* = 10.1, 7.4, 3.3 Hz, 1H), 2.68 (p, *J* = 6.5 Hz, 1H), 2.61 (dd, *J* = 9.4, 4.4 Hz, 1H), 2.36 (dd, *J* = 9.4, 7.7 Hz, 1H), 2.03–1.98 (m, 1H), 1.61 (dd, *J* = 12.6, 9.8 Hz, 1H), 1.01 (d, *J* = 6.5 Hz, 3H), 0.92 (d, *J* = 6.5 Hz, 3H).

^13^C NMR (151 MHz, DMSO-d_6_) *δ* 156.67 (d, *J* = 233.3 Hz), 141.20 (d, *J* = 3.9 Hz), 136.90 (d, *J* = 9.9 Hz), 126.39, 122.61 (dd, *J* = 9.7, 4.6 Hz), 121.76 (dd, *J* = 6.1, 3.8 Hz), 120.81 (d, *J* = 3.6 Hz), 118.97, 113.36 (d, *J* = 25.4 Hz), 110.76 (d, *J* = 8.9 Hz), 109.92 (d, *J* = 2.9 Hz), 105.98 (dd, *J* = 24.0, 5.4 Hz), 77.98, 73.17, 64.81, 49.52, 46.79, 45.16, 45.04, 18.67, 17.92.

##### 2.2.7.24. 1-((1,3-Bis(3-fluoro-9H-carbazol-9-yl)propan-2-yl)oxy)-3-(cyclopropylamino)propan-2-ol (WK-24)

White solid, 69.0 mg (yield 51%). IR (KBr), ν, cm^−1^: 2929, 1629, 1585, 1486, 1463, 1323, 1282, 1167, 797, 743, 720.

m.p. 57–59 °C. HRMS-ESI calcd. for C_33_H_32_F_2_N_3_O_2_ [M + H]^+^ 540.2463, found 540.2451.

^1^H NMR (600 MHz, DMSO-d_6_) *δ* 8.16 (dd, *J* = 7.8, 2.9 Hz, 2H), 8.00 (dt, *J* = 9.1, 2.8 Hz, 2H), 7.68 (ddd, *J* = 19.9, 9.9, 6.1 Hz, 3H), 7.62 (d, *J* = 8.3 Hz, 1H), 7.48–7.42 (m, 2H), 7.30 (td, *J* = 9.0, 2.0 Hz, 2H), 7.19 (td, *J* = 7.4, 4.6 Hz, 2H), 4.71–4.58 (m, 4H), 4.20 (tt, *J* = 8.3, 4.1 Hz, 1H), 3.99 (s, 1H), 2.74 (dt, *J* = 7.6, 3.8 Hz, 1H), 2.46 (dd, *J* = 9.5, 4.8 Hz, 1H), 2.32 (dd, *J* = 9.5, 7.3 Hz, 1H), 1.84 (dd, *J* = 12.1, 3.8 Hz, 1H), 1.58 (tt, *J* = 6.8, 3.6 Hz, 1H), 1.52 (dd, *J* = 12.2, 7.8 Hz, 1H), 0.17 (dtt, *J* = 14.1, 10.7, 5.0 Hz, 2H), −0.03–-0.13 (m, 2H).

^13^C NMR (151 MHz, DMSO-d_6_) *δ* 156.68 (d, *J* = 232.9 Hz), 141.20 (d, *J* = 11.5 Hz), 136.98 (d, *J* = 5.6 Hz), 126.31, 122.62 (d, *J* = 9.8 Hz), 121.80 (d, *J* = 4.3 Hz), 120.81, 118.92, 113.26 (d, *J* = 25.2 Hz), 110.78 (dd, *J* = 8.9, 2.7 Hz), 109.94 (d, *J* = 4.8 Hz), 105.95 (d, *J* = 23.7 Hz), 77.98, 73.99, 67.50, 51.66, 45.26, 29.84, 5.92, 5.66.

##### 2.2.7.25. 1-((1,3-Bis(3-fluoro-9H-carbazol-9-yl)propan-2-yl)oxy)-3-(tert-butylamino)propan-2-ol (WK-25)

White solid, 26.0 mg (yield 19%). IR (KBr), ν, cm^−1^: 3353, 2779, 1584, 1488, 1323, 1283, 1167, 857, 797, 743, 721.

m.p. 183–185 °C. HRMS-ESI calcd. for C_34_H_36_F_2_N_3_O_2_ [M + H]^+^ 556.2776, found 556.2768.

^1^H NMR (600 MHz, DMSO-d_6_) *δ* 8.18 (d, *J* = 7.8 Hz, 2H), 8.02 (dt, *J* = 9.2, 2.2 Hz, 2H), 7.74–7.63 (m, 4H), 7.46 (q, *J* = 8.1 Hz, 2H), 7.32 (qd, *J* = 9.0, 2.6 Hz, 2H), 7.20 (q, *J* = 7.4 Hz, 2H), 5.04 (brs, 1H), 4.66 (qd, *J* = 15.3, 6.1 Hz, 4H), 4.29 (tt, *J* = 8.1, 4.1 Hz, 1H), 3.18 (s, 1H), 2.64 (dd, *J* = 9.3, 4.2 Hz, 1H), 2.46–2.41 (m, 1H), 2.15 (d, *J* = 11.4 Hz, 1H), 1.62 (t, *J* = 10.8 Hz, 1H), 1.21 (s, 1H), 0.98 (s, 9H).

^13^C NMR (151 MHz, DMSO-d_6_) *δ* 156.70 (d, *J* = 232.7 Hz), 141.22 (d, *J* = 7.2 Hz), 136.97 (d, *J* = 11.1 Hz), 126.46, 122.65 (d, *J* = 9.9 Hz), 121.82 (d, *J* = 2.2 Hz), 120.90 (d, *J* = 8.9 Hz), 119.02, 113.44 (dd, *J* = 25.2, 3.2 Hz), 110.79 (t, *J* = 9.8 Hz), 109.93 (d, *J* = 5.1 Hz), 106.05 (dd, *J* = 23.7, 10.4 Hz), 78.01, 73.19, 65.50, 45.21, 45.16, 43.89, 25.06, 0.15.

##### 2.2.7.26. General methods for preparation of target compounds (WK-26, WK-2 8–30). (2R)-tert-butyl 4-(3-((1-(3,6-difluoro-9H-carbazol-9-yl)-3-(3-fluoro-9H-carbazol-9-yl)propan-2-yl)oxy)-2-hydroxypropyl)-2-(hydroxymethyl)piperazine-1-carboxylate (WK-26)

A mixture of intermediate compound **d6** (100.0 mg, 0.2 mmol, 1 equiv.) and **e1** (86.0 mg, 0.4 mmol, 2 equiv.) dissolved in 5 ml isopropyl alcohol, was introduced into a 10 ml sealed tube. The mixture was stirred at 60 °C and monitored by TLC until compound **d4** was completely consumed. The mixture was treated with EtOAc and H_2_O. The organic layer was washed with saturated aqueous NaCl, dried over anhydrous Na_2_SO_4_, and concentrated *in vacuo*. Final flash column chromatography utilising PE/EA (1:1) as the eluent afforded *(2 R)-tert-butyl 4-(3-((1-(3,6-difluoro-9H-carbazol-9-yl)-3-(3-fluoro-9H-carbazol-9-yl)propan-2-yl)oxy)-2-hydroxypropyl)-2-(hydroxymethyl)piperazine-1-carboxylate* (**WK-26**). White solid, 42.0 mg (yield 29%).

IR (KBr), ν, cm^−1^: 3403, 2932, 1676, 1488, 1465, 1167, 1147, 1120, 857, 793, 745.

m.p. 74–76 °C. HRMS-ESI calcd. for C_40_H_44_F_3_N_4_O_5_ [M + H]^+^ 717.3264, found 717.3263.

^1^H NMR (600 MHz, DMSO-d_6_) *δ* 8.16 (t, *J* = 7.3 Hz, 1H), 8.06–7.97 (m, 3H), 7.75–7.60 (m, 4H), 7.46 (dt, *J* = 15.9, 7.7 Hz, 1H), 7.32 (dtd, *J* = 26.0, 9.2, 2.6 Hz, 3H), 7.19 (q, *J* = 7.1 Hz, 1H), 4.76–4.39 (m, 5H), 4.23 (dt, *J* = 8.0, 4.1 Hz, 1H), 3.84–3.59 (m, 2H), 3.42 (d, *J* = 8.1 Hz, 1H), 3.34–3.24 (m, 1H), 3.21 (s, 1H), 2.79 (s, 1H), 2.59–2.51 (m, 1H), 2.43–2.31 (m, 2H), 2.00–1.87 (m, 1H), 1.67–1.44 (m, 1H), 1.43–1.31 (m, 11H), 1.29–1.18 (m, 2H).

^13^C NMR (151 MHz, DMSO-d_6_) *δ* 156.69 (d, *J* = 232.8 Hz), 156.52 (d, *J* = 233.2 Hz), 141.27, 137.83, 137.01, 126.35 (d, *J* = 6.0 Hz), 122.64 (d, *J* = 10.0 Hz), 122.19 (dd, *J* = 10.1, 4.2 Hz), 121.82, 120.79, 118.91, 114.12 (d, *J* = 25.5 Hz), 113.27 (d, *J* = 25.0 Hz), 111.23 (d, *J* = 9.4 Hz), 110.75 (d, *J* = 9.2 Hz), 109.97 (d, *J* = 11.3 Hz), 106.35 (d, *J* = 23.8 Hz), 105.93 (d, *J* = 23.8 Hz), 78.54, 77.89, 73.97, 73.65, 66.09, 60.26, 58.68, 54.97, 52.87, 51.95, 45.30, 28.08.

##### 2.2.7.27. 1-((1-(3,6-Difluoro-9H-carbazol-9-yl)-3-(3-fluoro-9H-carbazol-9-yl) propan-2-yl)oxy)-3-((R)-3-(hydroxymethyl)piperazin-1-yl)propan-2-ol (WK-27)

A mixture of **WK-26** (207.0 mg, 0.3 mmol, 1 equiv.) and 2 N hydrogen chloride-1, 4-Dioxane solution 5 ml was introduced into a 10 ml sealed tube. The mixture was stirred at room temperature and monitored by TLC until **WK-26** was completely consumed. The mixture was extracted with EtOAc and H_2_O, the organic layer was washed with saturated aqueous NaCl, dried over anhydrous Na_2_SO_4_, and concentrated *in vacuo*. Final flash column chromatography utilising DCM/MeOH (15:1) as the eluent afforded *1-((1-(3,6-difluoro-9H-carbazol-9-yl)-3-(3-fluoro-9H-carbazol-9-yl)propan-2-yl)oxy)-3-((R)-3-(hydroxymethyl)piperazin-1-yl)propan-2-ol* (**WK-27**). White solid, 123.0 mg (yield 69%).

IR (KBr), ν, cm^−1^: 3343, 2933, 1585, 1488, 1465, 1167, 1146, 1120, 858, 792, 746.

m.p. 150–152 °C. HRMS-ESI calcd. for C_35_H_36_F_3_N_4_O_3_ [M + H]^+^ 617.2740, found 617.2728.

^1^H NMR (600 MHz, DMSO-d_6_) *δ* 9.18 (brs, 1H), 8.71 (brs, 1H), 8.17 (d, *J* = 7.7 Hz, 1H), 8.02 (ddd, *J* = 18.9, 9.1, 2.3 Hz, 3H), 7.78–7.63 (m, 4H), 7.52–7.40 (m, 1H), 7.39–7.27 (m, 3H), 7.19 (t, *J* = 7.5 Hz, 1H), 5.38 (dt, *J* = 8.9, 4.6 Hz, 1H), 4.64 (dt, *J* = 11.1, 5.6 Hz, 4H), 4.22 (h, *J* = 5.8 Hz, 1H), 3.95 (t, *J* = 5.2 Hz, 1H), 3.51–3.45 (m, 1H), 2.91 (d, *J* = 11.7 Hz, 1H), 2.81 (s, 1H), 2.70 (s, 1H), 2.61–2.52 (m, 1H), 2.48–2.37 (m, 1H), 2.34–2.09 (m, 3H), 1.94–1.62 (m, 2H), 1.50–1.36 (m, 1H), 1.22 (d, *J* = 12.6 Hz, 1H).

^13^C NMR (151 MHz, DMSO-d_6_) *δ* 156.67 (d, *J* = 233.0 Hz), 156.48 (d, *J* = 233.5 Hz), 141.33, 137.84, 137.04, 126.37, 122.64 (d, *J* = 9.8 Hz), 122.13 (dd, *J* = 10.0, 4.3 Hz), 121.80 (d, *J* = 4.1 Hz), 120.86, 118.95, 114.12 (d, *J* = 25.6 Hz), 113.30 (d, *J* = 24.9 Hz), 111.28 (d, *J* = 9.0 Hz), 110.86 (d, *J* = 8.4 Hz), 110.05, 106.39 (d, *J* = 23.8 Hz), 106.01 (d, *J* = 23.8 Hz), 77.86, 73.41, 66.57, 59.62, 59.19, 55.84, 51.56, 49.34, 45.44, 45.28, 42.52.

##### 2.2.7.28. 1-((2-(1H-indol-3-yl)ethyl)amino)-3-((1-(3,6-difluoro-9H-carbazol-9-yl)-3-(3-fluoro-9H-carbazol-9-yl)propan-2-yl)oxy)propan-2-ol (WK-28)

White solid, 72.0 mg (yield 55%). IR (KBr), ν, cm^−1^: 2928, 1585, 1488, 1463, 1281, 1168, 1146, 1120, 857, 792, 744.

m.p. 113–115 °C. HRMS-ESI calcd. for C_40_H_36_F_3_N_4_O_2_ [M + H]^+^ 661.2790, found 661.2784.

^1^H NMR (600 MHz, DMSO-d_6_) *δ* 11.03 (s, 1H), 8.34 (s, 1H), 8.11 (d, *J* = 7.8 Hz, 1H), 8.05–7.92 (m, 3H), 7.79–7.62 (m, 4H), 7.53 (d, *J* = 7.9 Hz, 1H), 7.48–7.41 (m, 1H), 7.42–7.29 (m, 4H), 7.18 (d, *J* = 2.0 Hz, 1H), 7.13 (dt, *J* = 14.2, 7.3 Hz, 2H), 7.06–7.00 (m, 1H), 4.99 (s, 1H), 4.77–4.59 (m, 4H), 4.22 (p, *J* = 7.1 Hz, 1H), 3.11 (s, 1H), 2.85 (dd, *J* = 11.6, 6.0 Hz, 2H), 2.61 (ddq, *J* = 18.8, 11.6, 6.8 Hz, 2H), 2.52 (d, *J* = 4.4 Hz, 1H), 2.33–2.27 (m, 1H), 2.05 (d, *J* = 10.8 Hz, 1H), 1.75–1.65 (m, 1H).

^13^C NMR (151 MHz, DMSO-d_6_) *δ* 156.69 (d, *J* = 233.1 Hz), 156.49 (d, *J* = 233.4 Hz), 141.26, 137.80, 136.93, 136.28, 126.79, 126.40, 122.99, 122.62 (d, *J* = 9.7 Hz), 122.15 (dd, *J* = 10.0, 4.0 Hz), 121.76 (d, *J* = 4.0 Hz), 121.22, 120.82, 118.96, 118.46, 118.18, 114.21 (d, *J* = 25.7 Hz), 113.40 (d, *J* = 25.5 Hz), 111.57, 111.26 (d, *J* = 9.0 Hz), 110.83 (d, *J* = 8.8 Hz), 110.00, 106.42 (d, *J* = 23.7 Hz), 106.01 (d, *J* = 23.8 Hz), 78.24, 73.29, 64.63, 54.97, 49.66, 47.51, 45.29, 45.20, 21.76.

##### 2.2.7.29. 1-((3-Butoxypropyl)amino)-3-((1-(3,6-difluoro-9H-carbazol-9-yl)-3-(3-fluoro-9H-carbazol-9-yl)propan-2-yl)oxy)propan-2-ol (WK-29)

White solid, 92.0 mg (yield 73%). IR (KBr), ν, cm^−1^: 3323, 2932, 2867, 1585, 1488, 1467, 1296, 1109, 855, 794, 745.

m.p. 151–153 °C. HRMS-ESI calcd. for C_37_H_41_F_3_N_3_O_3_ [M + H]^+^ 632.3100, found 632.3088.

^1^H NMR (600 MHz, DMSO-d_6_) *δ* 8.24 (brs, 1H), 8.17 (d, *J* = 7.8 Hz, 1H), 8.03 (ddd, *J* = 20.0, 9.1, 2.6 Hz, 3H), 7.80–7.67 (m, 4H), 7.49 (t, *J* = 7.3 Hz, 1H), 7.42–7.29 (m, 3H), 7.20 (t, *J* = 7.5 Hz, 1H), 4.98 (brs, 1H), 4.74–4.60 (m, 4H), 4.22 (p, *J* = 6.2 Hz, 1H), 3.42–3.31 (m, 5H), 3.07 (s, 1H), 2.44–2.31 (m, 2H), 2.29–2.23 (m, 1H), 1.99–1.89 (m, 1H), 1.69–1.53 (m, 3H), 1.48 (p, *J* = 6.6 Hz, 2H), 1.31 (h, *J* = 7.4 Hz, 2H), 0.88 (td, *J* = 7.4, 2.6 Hz, 3H).

^13^C NMR (151 MHz, DMSO-d_6_) *δ* 156.70 (d, *J* = 233.1 Hz), 156.50 (d, *J* = 233.5 Hz), 141.27, 137.82, 136.97, 126.42, 122.63 (d, *J* = 9.8 Hz), 122.14 (dd, *J* = 9.9, 4.0 Hz), 121.79 (d, *J* = 4.4 Hz), 120.85, 118.99, 114.21 (d, *J* = 25.4 Hz), 113.41 (d, *J* = 25.4 Hz), 111.29 (d, *J* = 9.2 Hz), 110.87 (d, *J* = 8.7 Hz), 110.04, 106.43 (d, *J* = 24.0 Hz), 106.02 (d, *J* = 23.8 Hz), 78.18, 73.21, 69.87, 67.15, 64.38, 49.58, 45.26, 45.18, 45.00, 31.27, 25.67, 18.91, 13.86.

##### 2.2.7.30. 1-((1-(3,6-Difluoro-9H-carbazol-9-yl)-3-(3-fluoro-9H-carbazol-9-yl) propan-2-yl)oxy)-3-isopropoxypropan-2-ol (WK-30)

Pale yellow liquid, 43.0 mg (yield 36%). IR (KBr), ν, cm^−1^: 3323, 2932, 2867, 1585, 1488, 1467, 1296, 1109, 855, 794, 745.

Liquid. HRMS-ESI calcd. for C_33_H_32_F_3_N_2_O_3_ [M + H]^+^ 561.2365, found 561.2338.

^1^H NMR (600 MHz, DMSO-d_6_) *δ* 8.16 (d, *J* = 7.7 Hz, 1H), 8.02 (ddd, *J* = 14.3, 9.2, 2.5 Hz, 3H), 7.69 (ddd, *J* = 16.8, 8.8, 4.1 Hz, 4H), 7.46 (t, *J* = 8.1 Hz, 1H), 7.37–7.28 (m, 3H), 7.19 (td, *J* = 7.4, 3.6 Hz, 1H), 4.64 (d, *J* = 5.9 Hz, 4H), 4.20 (p, *J* = 6.9, 6.3 Hz, 1H), 4.01 (d, *J* = 5.2 Hz, 1H), 2.74 (p, *J* = 6.1 Hz, 1H), 2.69–2.61 (m, 1H), 2.43–2.31 (m, 3H), 2.13 (dd, *J* = 9.7, 6.4 Hz, 1H), 0.71 (dd, *J* = 15.1, 6.1 Hz, 6H).

^13^C NMR (151 MHz, DMSO-d_6_) *δ* 156.69 (d, *J* = 233.0 Hz), 156.49 (d, *J* = 233.1 Hz), 141.27, 137.82, 137.01, 126.31, 122.65 (d, *J* = 9.8 Hz), 122.16 (dd, *J* = 9.9, 4.2 Hz), 121.81 (d, *J* = 4.1 Hz), 120.82, 118.92, 114.04 (d, *J* = 25.7 Hz), 113.24 (d, *J* = 25.3 Hz), 111.16 (d, *J* = 8.9 Hz), 110.78 (d, *J* = 9.6 Hz), 109.96, 106.32 (d, *J* = 24.0 Hz), 105.97 (d, *J* = 23.8 Hz), 77.91, 72.30, 70.63, 68.83, 68.19, 45.44, 45.25, 21.87, 21.60.

### 2.3. Biological section

#### 2.3.1. DNMT1 inhibition assays

##### 2.3.1.1. ELISA DNMT1 activity assay

All the compounds were first screened using an ELISA EpiQuik DNA methyltransferase (DNMT) activity/inhibitor assay kit (Epigentek). To measure the effects of the compounds on human DNMT1 activity, 200 nM purified DNMT1 was incubated with 50 µM and 100 µM of the different compounds and S-adenosylmethionine in the DNMT assay buffer in the assay plate at 37 °C for 2 h^36^. Next, every sample was incubated with the capture and detection antibody, followed by incubation with developer solution for 10 min at room temperature. The absorbance was measured at 450 nm using a POLARstar Omega microplate reader (BMG). S-Adenosylhomocysteine (AdoHcy) was used as a positive control. Methylation inhibition assays for EZH2, LSD, and G9a were performed in modified Tris, pH 9.0, buffer using AlphaLisa technology. An amount of 10 µL of the reaction system contained a corresponding concentration of SAM (Sigma) (EZH2 50 µM, LSD 50 µM, and G9a 50 µM), which was the Km value in each enzymatic reaction, plus 100 nM biotinylated peptide H3 (1–21) (synthesis by GLChina) and the relevant enzyme concentration (0.03 nM EZH2, 0.03 nM LSD, and 0.03 nM G9a). The proteins were preincubated with various compound concentrations for 15 min at room temperature before the substrate and SAM were added. After 60 min of incubation at room temperature, acceptor and donor AlphaLisa beads were added according to the manufacturer’s recommendations. The signals were read in Alpha mode with an EnSpire multimode plate reader (PerkinElmer). IC_50_ values were derived by fitting the data for the inhibition percentage to a dose-response curve by non-linear regression in GraphPad Prism 5.0^20^.

##### 2.3.1.2. Radioactive methylation assay

The DNMT1 radioactive methylation inhibition assays were performed in 30 µL reactions containing 0.1 µM adenosyl-L-methionine S-[methyl-^3^H] (^3^H-SAM, ^15 ^Ci/mmol, PerkinElmer), 0.25 µg/mL poly (dI-dC)·poly (dI-dC) (Sigma), 40 nM DNMT1 in 50 mM Tris-HCl, pH 8.0, 1 mM DTT, 5% glycerol, and 100 µg/mL BSA. The proteins were preincubated with a range of compound concentrations for 15 min at room temperature before adding the substrate and [^3^H] SAM. After 60 min of incubation at 37 °C, the reaction systems were transferred to a MultiScreen HTS filter plate (Millipore), and the plate was washed 3 times with doubly distilled H_2_O *via* a vacuum. The radioactivity was determined by liquid scintillation counting (MicroBeta, PerkinElmer). IC_50_ values were derived by fitting the data for the inhibition percentage to a dose-response curve by non-linear regression in GraphPad Prism 5.0.

#### 2.3.2. Cell lines and culture conditions

The human lung cancer cells (A549) and human colon cancer cells (HCT116) were cultured in an RPMI-1640 medium containing 10% FBS, 100 U/mL streptomycin, and 100 U/mL penicillin at 37 °C in a humidified atmosphere with 5% CO_2_. All compounds were dissolved in DMSO and stock solutions were stored at −20 °C. Reagents were freshly diluted to the marked concentrations with a culture medium before use. DMSO concentration in experimental conditions never exceeded 0.1% (v/v). All cell lines were provided by the cell laboratory, School of pharmaceutical sciences, Guangzhou Medical University.

#### 2.3.3. MTT assay

Cell viability was detected using an MTT assay kit. Cells were seeded into 96-well plates at a density of ∼1.0 × 10^4^/well. 24 h later, sextuplicate wells were treated with media and new compounds at a fixed concentration (50 µM). After 24, 48, and 72 h, the drug-containing medium was replaced by a 100 µL fresh medium with 5 mg/mL MTT solution. After 4 h of incubation, the medium with MTT was removed, and 100 µL of DMSO was added to each well. The plates were gently agitated until the purple formazan crystals were dissolved, and the OD490 was determined using a microscope (Olympus BX53, Japan). The data were calculated and plotted as the percent viability compared to the control.

### 2.4. PK study

*In vivo* pharmacokinetic properties of **WK-22**, **WK-23**, **WK-27**, and **DC_517** were performed by Medicilon Company, Shanghai, China. SPF-grade SD male rats (8 groups, *n* = 3 rats per group) with a body weight of 230–260 g were purchased from Shanghai SIPPR-BK LAB Animal Ltd., Shanghai, China, and used for the pharmacokinetic analysis of the tested compounds. All animals were deprived of food overnight after the cannulation surgery. Subsequently, the tested compounds **WK-23** and **DC_517** were dissolved/suspended in 5% DMSO, 10% Solutol, and 85% water, and the tested compounds **WK-22** and **WK-27** were dissolved/suspended in 5% DMSO, 40% PEG400, and 55% saline for intravenous administration (i.v.) and oral administration (p.o.), respectively. A final dosage of 2.0 and 10.0 mg/kg rat of the formulated compounds was administered for i.v. and p.o. purposes, respectively, and the blood samples were taken at various time points during a 24 h period. At different time points, blood samples were collected from the femoral vein. The plasma samples were obtained after centrifugation (6800 g, 6 min, 2–8 °C) and stored at −80 °C until the assay. The AUC (area under concentration-time curve) was calculated through the trapezoidal rule with extrapolation to time infinity. The concentration of the compounds in the blood was analysed by LC-MS/MS (Shimadzu liquid chromatographic system and SCIEX Triple Quad 5500+ mass spectrometer, Applied Biosystems, Ontario, Canada). The *T*_max_, *T*_1/2_, and *C*_max_ value was obtained through visual inspection of the plasma concentration-time curve. The Vss value was generated from DAS 3.2.8 software. The F value of **WK-23** was calculated with the formula: F = (AUC p.o. × Dose i.v.)/(AUC i.v. × Dose p.o.) × 100%.

### 2.5. Molecular docking

Molecular docking was performed by the Glide program packed in Maestro (Maestro, Schrödinger, LLC, New York, NY, 2020.). The crystal structure of human DNMT1 was used as a template (PDB code: 4WXX) and removed the water molecules^20^. The docking procedure was initiated by the optimisation of protein structure using the Protein preparation Wizard module. Then the inhibitor was optimised by the Ligand Preparation module to generate stereoisomers and protonation states. Extra precision (XP) mode was used to perform the molecular docking, and the final result was selected through the Glide score function.

## 3. Results and discussion

### 3.1. Chemistry

**Scheme 1.**
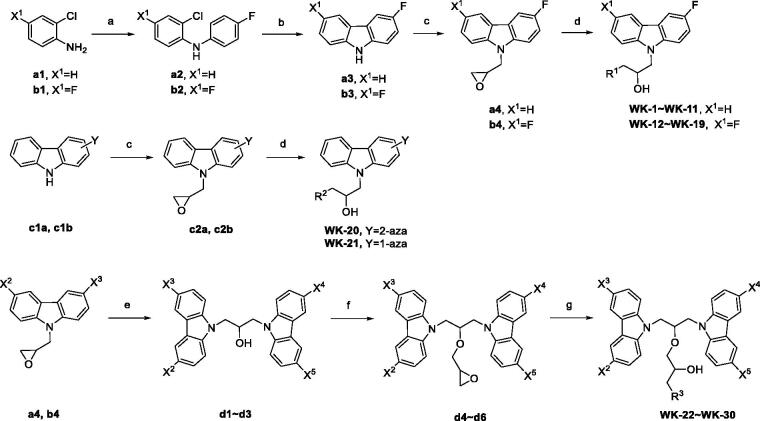
Reagents and conditions: (a) NaO*^t^*Bu, Pd(OAc)_2_, [HP*^t^*Bu_3_][BF_4_], 4-bromofluorobenzene, toluene, reflux, 4 h; (b) Pd(OAc)_2_, [HP*^t^*Bu_3_][BF_4_], NaOtBu, 1,4-dioxane, reflux, 18 h; (c) Epichlorohydrin, KOH, DMF, r.t. (d) Amines, EtOH, 60 °C; (e) *3-fluoro-9H-carbazole* (**a3**) or *3,6-difluoro-9H-carbazole* (**b3**), KOH, Na_2_SO_4_, acetone, r.t.; (f) Epichlorohydrin, KOH, Na_2_SO_4_, acetone, r.t.; (g) Amines, isopropanol, 60 °C.

The preparation routes of all target compounds are outlined in Scheme 1. First, we synthesised the key intermediates, and the general synthetic routes are illustrated in Scheme 1. Briefly, the commercially available 2-chloroaniline or 2-chloro-4-fluoroaniline reacted with bromofluorobenzene by the Buchwald-Hartwig coupling reaction and then the transition metal-catalyzed C-C coupling ring was constructed for 3 or 3, 6 substitution carbazole^21^. Fluorine-substituted carbazoles reacted with epichlorohydrin to get epoxy intermediates^22^. The general synthetic route of piperazine sidechain is illustrated in Scheme S1. Finally, the epoxy intermediates interacted with different amine compounds under basic conditions by nucleophilic substitution reaction to achieve 30 target compounds^23^. The structure of these compounds was confirmed by ^1^H NMR, ^13 ^C NMR, high-resolution mass spectra, and infra-red spectra.

### 3.2. DNMT1 inhibition assays

**Figure 2.**
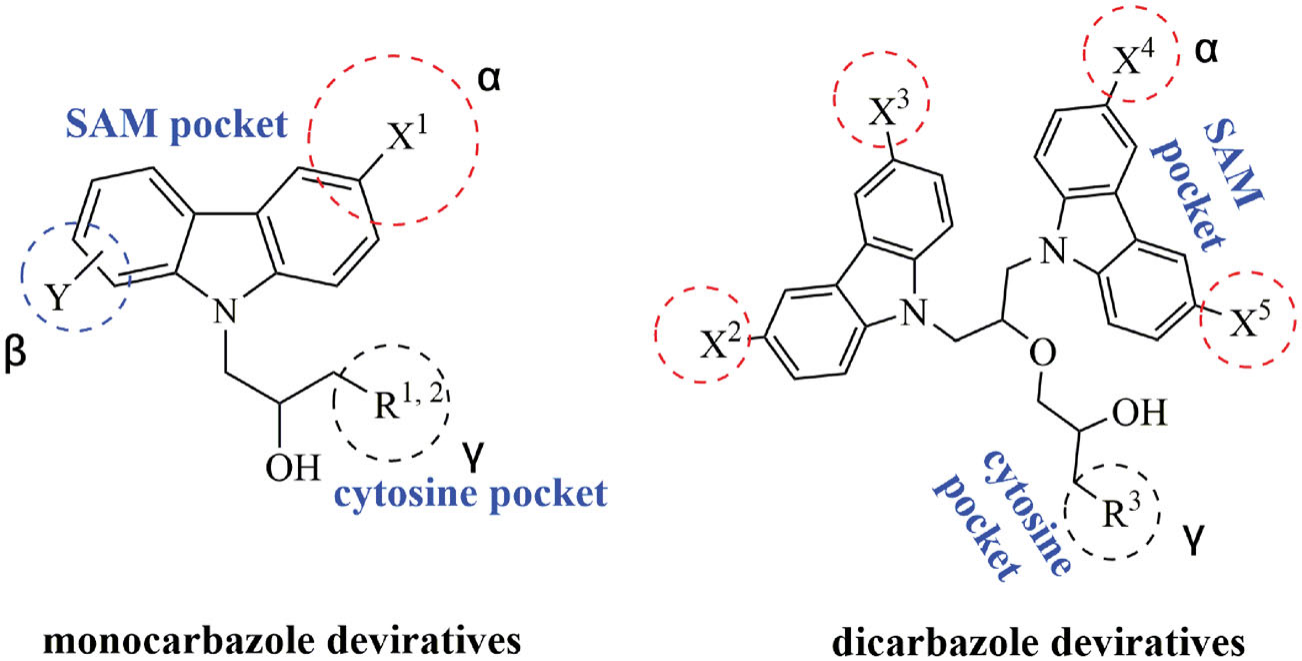
Schematic showing design for binding DNMT1 pharmacophore.

DNMT1 inhibition test was performed on 30 candidates to verify their biochemical activities. The EpiQuik DNA methyl-transferase (DNMT) activity/inhibitor assay kit (Epigentek) was used to identify the activity of synthetic compounds. It was found that 11 compounds could inhibit DNMT1 activity by >80% (Table 1, Figure 2), and these compounds had similar potency to that of **DC_517** against DNMT1 at a concentration of 50 µM and 100 µM.

**Table 1.** Biochemical assay results for **DC_05** and **DC_517** analogues against DNMT1 catalytic activity. 
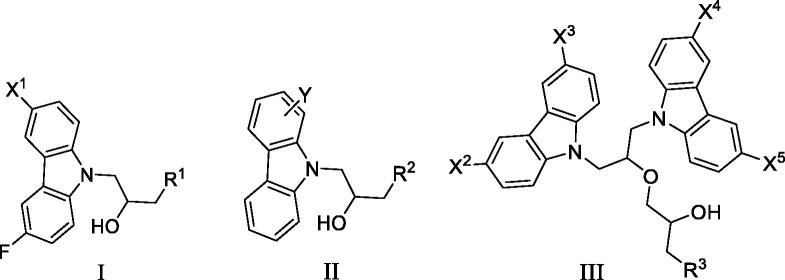

Cpd.	Scaffold	Substituent	^a^Inh% at 50 μM	^a^Inh% at 100 μM	^b^IC50 (μM)
**WK-1**	**I**	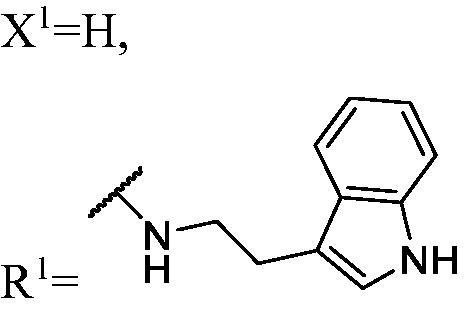	82	100	28
**WK-2**		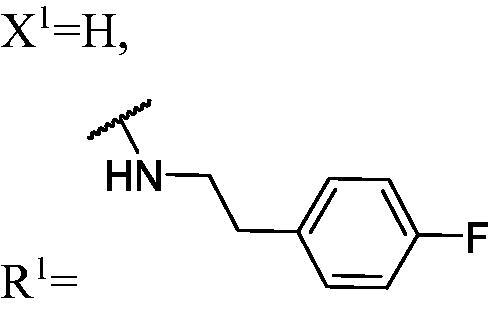	55	66	–
**WK-3**		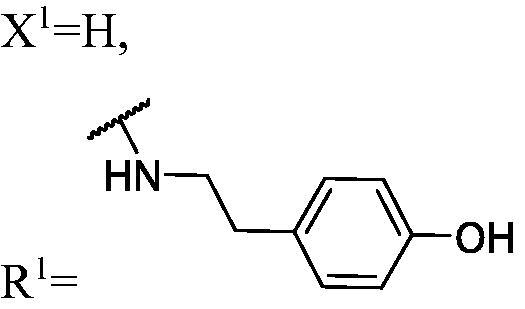	71	79	27
**WK-4**		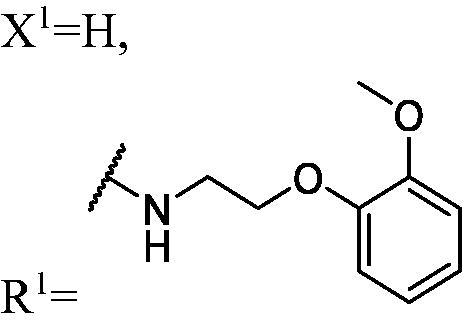	56	60	–
**WK-5**		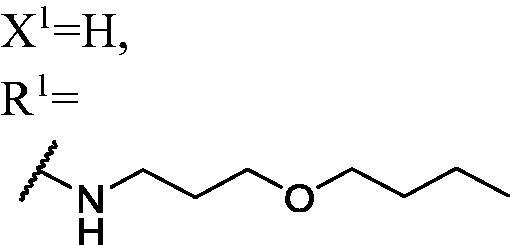	59	65	–
**WK-6**		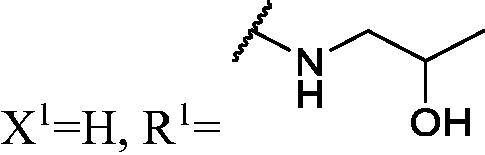	68	79	–
**WK-7**		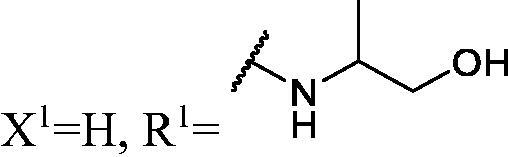	67	83	9.6
**WK-8**		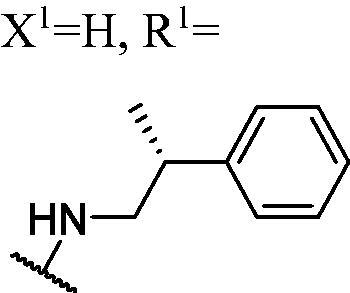	16	58	–
**WK-9**		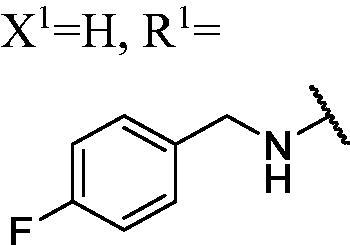	25	53	–
**WK-10**		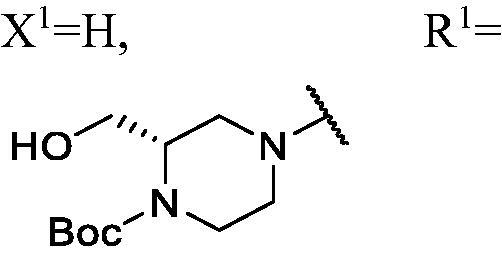	5.1	−9.0	–
**WK-11**		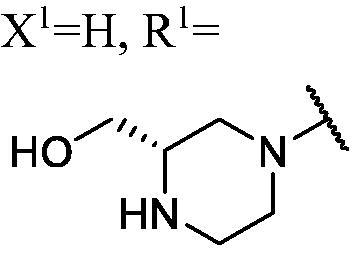	41	50	–
**WK-12**		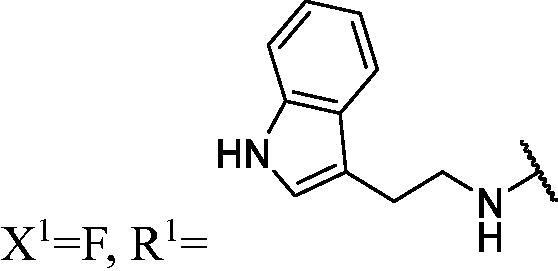	99	94	17
**WK-13**		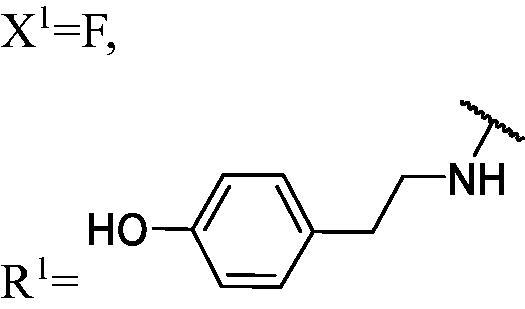	82	90	19
**WK-14**		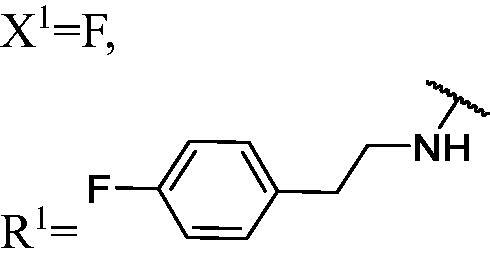	79	84	6.0
**WK-15**		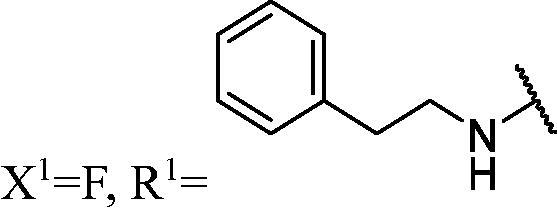	75	82	7.7
**WK-16**		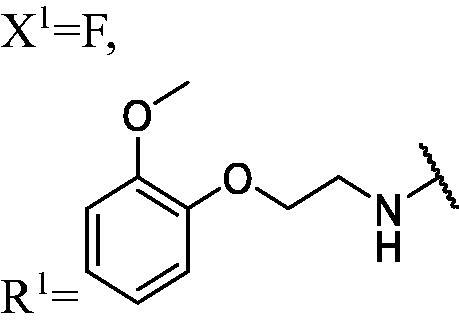	27	46	–
**WK-17**		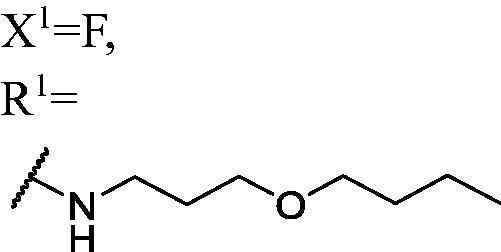	73	79	–
**WK-18**		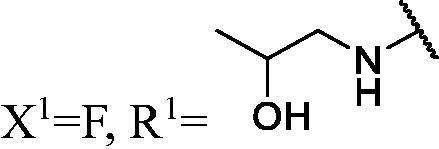	70	87	10
**WK-19**		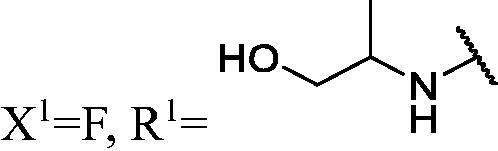	78	93	9.2
**WK-20**	**II**	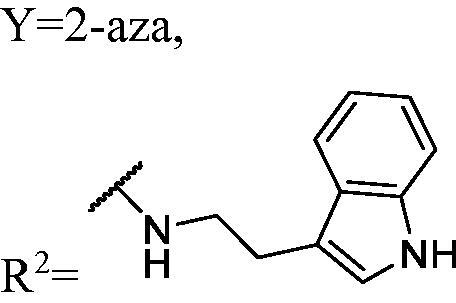	68	77	–
**WK-21**		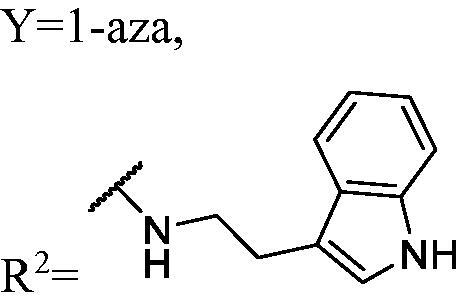	39	34	–
**DC_05**		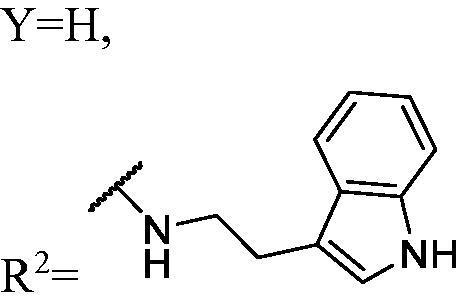	43	88	52
**WK-22**	**III**	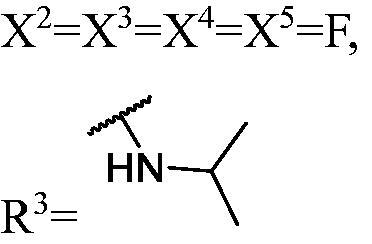	99	100	4.9
**WK-23**		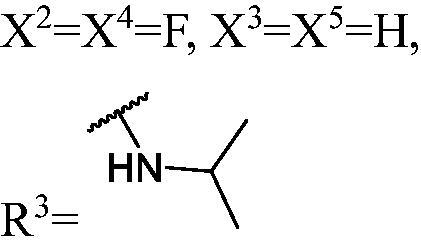	99	100	5.0
**WK-24**		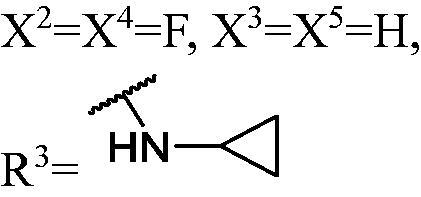	2.3	2.6	–
**WK-25**		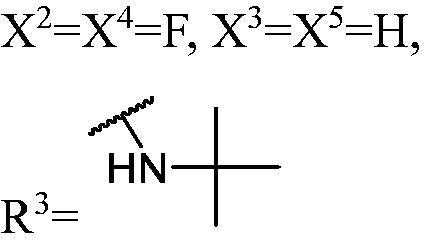	100	100	–
**WK-26**		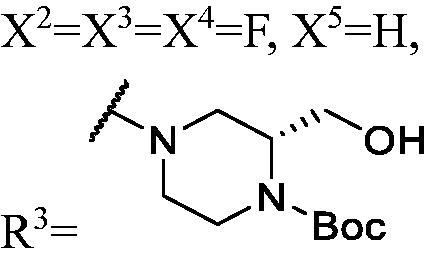	8.7	−16	–
**WK-27**		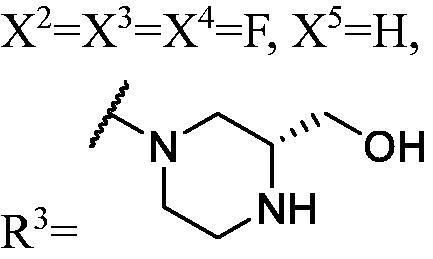	100	100	–
**WK-28**		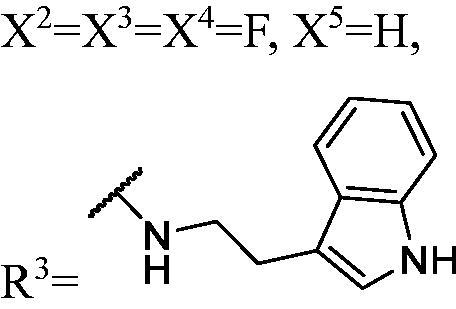	91	91	–
**WK-29**		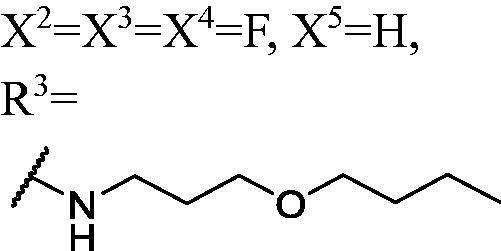	85	80	–
**WK-30**		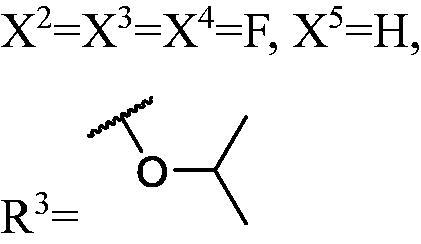	–	–	–
**DC_517**		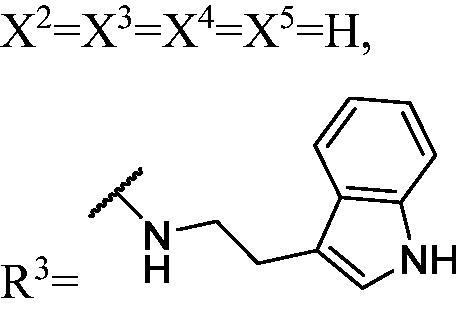	96	98	2.3

^a^Measured by EpiQuik DNA methyltransferase (DNMT) activity/inhibitor assay kit.

^b^Measured by H^3^-labeled radioactive methylation assay.

### 3.3. Radioactive methylation assay against DNMT1

To quantitatively analyse DNMT1 inhibitory effect, the H^3^-labelled radioactive methylation assay was conducted to measure the methyltransferase activity of DNMT1 at a range of concentrations for these compounds. As shown in Table 1, **WK-22** (IC_50_ = 4.9 µM) and **WK-23** (IC_50_ = 5.0 µM) showed similar inhibitory activity as **DC_517** (IC_50_ = 2.3 µM), whereas the other compounds were less potent. This result helped us to uncover sufficient information for the study of hit optimisation and structure-activity relationships (SARs).

### 3.4. Structure–activity relationships investigation

All the carbazole derivatives summarised in Scheme 1, including twenty-one derivatives of **DC_05** and nine derivatives of **DC_517** were prepared and biologically evaluated for the DNMT1 inhibitory activity. The experimental data of compounds bearing monocarbazole or dicarbazole as DNMT1 inhibitors are displayed in Table 1, respectively. The structure-activity relationships of **DC_05**, **DC_517**, and its analogs were determined and investigated by comparing the DNMT1 inhibitory activity.

Accordingly, as shown in Figure 2, the catalytic site of DNMT1 is thereby includes two parts, namely, the cofactor binding site SAM pocket and the substrate binding site cytosine pocket. In our previous study, we conducted the molecular docking study and found that the compounds occupied the SAM pocket and cytosine pocket of DNMT1 (PDB code 4DA4)^20^.

As shown in Table 1, **WK-1** and **WK-12** with fluorine substitute at C-3 or C-3,6 position, showed IC_50_ values of 28 and 17 µM, respectively, stronger than the lead compound **DC_05** (IC_50_ = 52 µM). And when comparing the activity between **WK-13**, **WK-14**, **WK-17**, **WK-18**, and **WK-19** from **WK-3**, **WK-2**, **WK-5**, **WK-6**, and **WK-7**, respectively, we found that difluoro-carbazole derivatives exhibited a better DNMT1 inhibition activity than the derivatives only replaced single fluorine on C-3 position.

**WK-20** and **WK-21** with a nitrogen heterocyclic replaced at region β, which showed lower inhibitory activity than **DC_05** at 100 µM. It could be that the lone pair on the nitrogen is not tolerated in the pocket either because the environment is non-polar or because there is actually already an atom with a lone pair that leads to a repulsive effect.

The result of enzyme inhibitory activities showed that different amines at region γ will lead to a different activity. When it comes to monocarbazole, the decreasing order of influence of amines in inhibition activity was tryptamine, phenethylamines, and aliphatic amines. It could be corresponding to the space structure of the cytosine pocket since the structures linked to the amines are too small to occupy the cytosine pocket completely. But **WK-6**, **WK-7**, **WK-18**, and **WK-19** still showed a relatively potent activity, possibly because the derivatives form extra hydrogen bonds *via* its hydroxyl to bind to the amino acid residue of the cytosine pocket. Similarly, phenethylamines derivative **WK-13** (IC_50_ = 19 µM), which has a hydrogen donor on the aromatic ring exhibited a better activity. The inhibition of **WK-2** and **WK-9** are inferiors to **DC_05**, which suggests a long linking chain is optimal for the distance of binding targets. This derivation is also consistent with the activity data of dicarbazole molecules. In addition, **WK-26** lost its activity probably because the Boc-protecting group resulted in steric hindrance while **WK-24** was also inactive perhaps because the cyclopropyl group reduces hydrophobic and weakens the binding with the cytosine pocket.

### 3.5. Methyltransferase enzymatic selectivity profiling

Based on the DNMT1 Inhibition Assays data, some derivatives showed a considerable DNMT1 inhibition activity. Monocarbazole molecules that exhibited better inhibition on DNMT1 than **DC_05**, or dicarbazole molecules that exhibited better inhibition than **DC_517** at 50 µM and 100 µM, were chosen to promote the next step. In addition to DNMT1, there are many other methyltransferases that can bind with S-adenosyl-_L_-methionine (SAM) to facilitate transmethylation reactions^24^^,^^25^. To investigate the selectivity of these potent compounds for DNMT1, we then evaluated the inhibitory activities against DNMT1 and other important methyltransferases at the concentration of 50 µM, including DNMT3A/3L, DNMT3B/3L, and other SAM-dependent enzymes^1^^,^^2^^,^^26^, such as EZH2^27^, LSD1^28^, G9a (histone H3 lysine 9 methyltransferase)^29^.

**Figure 3.**
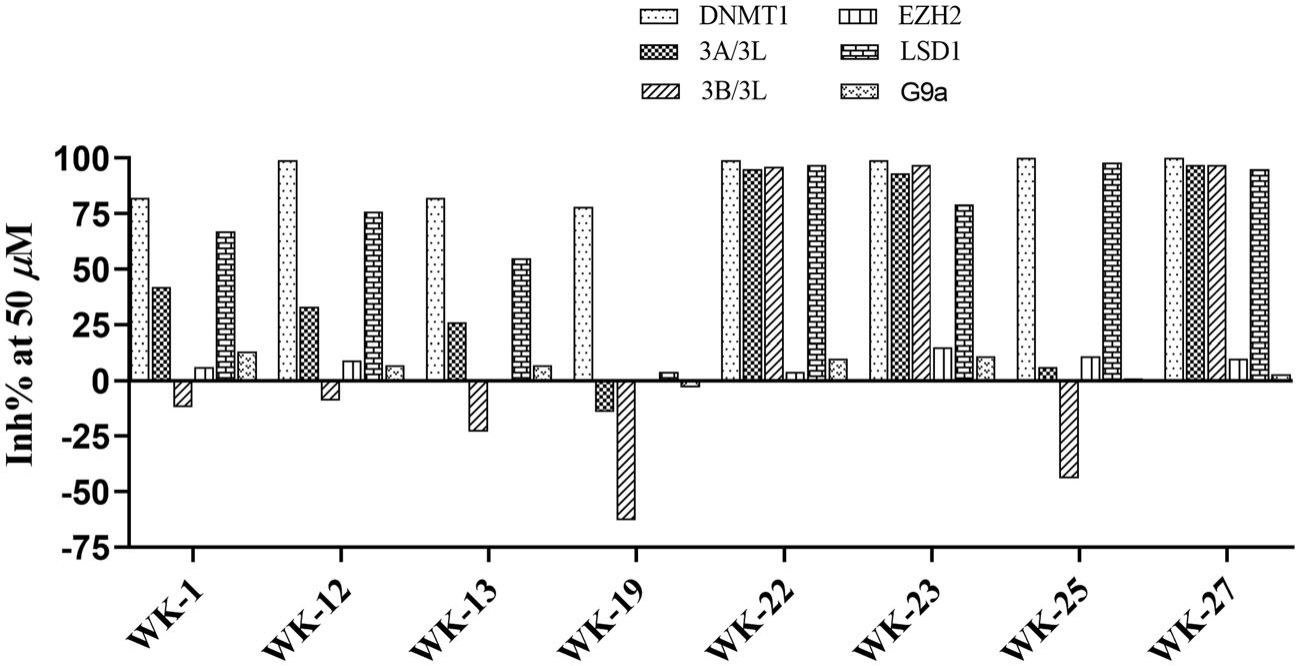
Enzymatic selectivity of preliminarily screened compounds (50 μM).

As shown in Figure 3, the enzymatic selectivity and inhibitory activity were measured at the concentration of 50 µM. Compounds **WK-1**, **WK-12**, and **WK-13** displayed a strong activity on DNMT1 while showing hardly any inhibitory activities against DNMT3B/3L, EZH2, and G9a. Compounds **WK-22**, **WK-23**, and **WK-27** displayed a similar activity targeting DNMT1 to that on DNMT3A/3L, DNMT3B/3L, and LSD1, while showed nearly no inhibitory activities against EZH2 and G9a. As shown in Table S1, WK-23 exhibited good selectivity on EZH2 and G9a (Relative selectivity index = 0.85 and 0.89), while had a similar effect on DNMT 3 A/3L, DNMT 3B/3L, and LSD1 to that on DNMT1 (Relative selectivity index = 0.06, 0.02 and 0.2). Here, the selectivity of non-nucleoside DNMT1 inhibitors is somewhat predictable because other methyltransferases mentioned above catalyse different substrates and share very low homology with DNMT1, even in their catalytic domain^30–32^. Subsequently, the screened compounds were evaluated in a dose-dependent assay against HCT116 (human colon cancer cell line) and A549 (human lung carcinoma cell line).

### 3.6. Pharmacokinetics (PKs)

By virtue of the favourable DNMT1 inhibitory activity and specificity, **WK-22**, **WK-23**, **WK-27** were selected for further PK evaluation in Sprague-Dawley (SD) Rats with **DC_517** used as the reference^33^. As shown in Table 2, **WK-22** gave an AUC of 867.2 ± 15.9 h ng/mL and oral bioavailability of 27.0%, whereas **WK-27** gave an AUC of 427.2 ± 60.0 h ng/mL and oral bioavailability of 1.8%. This is perhaps because the introduction of piperazine leads to the reduction of permeability and absorption into the blood. **WK-23** displayed a favourable plasma exposure (AUC_0-t_ = 1064.9 ± 121.2 h ng/mL) and an acceptable oral bioavailability (*F*% = 37.1 ± 1.7), which is equivalent to **DC_517** (AUC_0-t_ = 1022.7 ± 60.9 h ng/mL, *F*% = 38.7 ± 2.9). Most strikingly, the elimination half-life of **WK-23** (*T*_1/2_ = 7.9 h) has an advantage over **DC_517** (*T*_1/2_ = 6.7 h).

**Table 2.** The PK properties of selected compound **WK-22**, **WK-27**, **WK-23**, and **DC_517**

Cpd.	Administration route and dosage	*T*_1/2_ (h)	*T*_max_ (h)	AUC_(0–_*_t_*_)_ (h*ng/mL)	*F* (%)
**WK-22**	i.v. (2 mg/kg)	12.2 ± 0.8	0.1 ± 0.0	867.2 ± 15.9	27.0 ± 4.6
	p.o. (10 mg/kg)	8.8 ± 1.3	7.3 ± 1.2	1172.5 ± 197.3	
**WK-27**	i.v. (2 mg/kg)	11.5 ± 1.2	0.1 ± 0.0	427.2 ± 60.0	1.8 ± 1.9
	p.o. (10 mg/kg)	3.9 ± 2.5	2.7 ± 1.2	28.5 ± 40.0	
**WK-23**	i.v. (2 mg/kg)	7.9 ± 1.0	0.08 ± 0.0	1064.9 ± 121.2	37.1 ± 1.7
	p.o. (10 mg/kg)	6.5 ± 0.3	6.7 ± 1.2	1973.9 ± 91.1	
**DC_517**	i.v. (2 mg/kg)	6.7 ± 0.2	0.08 ± 0.0	1022.7 ± 60.9	38.7 ± 2.9
	p.o. (10 mg/kg)	6.7 ± 0.9	6.0 ± 0.0	2079.5 ± 112.8	

Data were shown as mean ± *SD* (*n* = 3).

### 3.7. Evaluation of antitumor activity in cells

**Figure 4.**
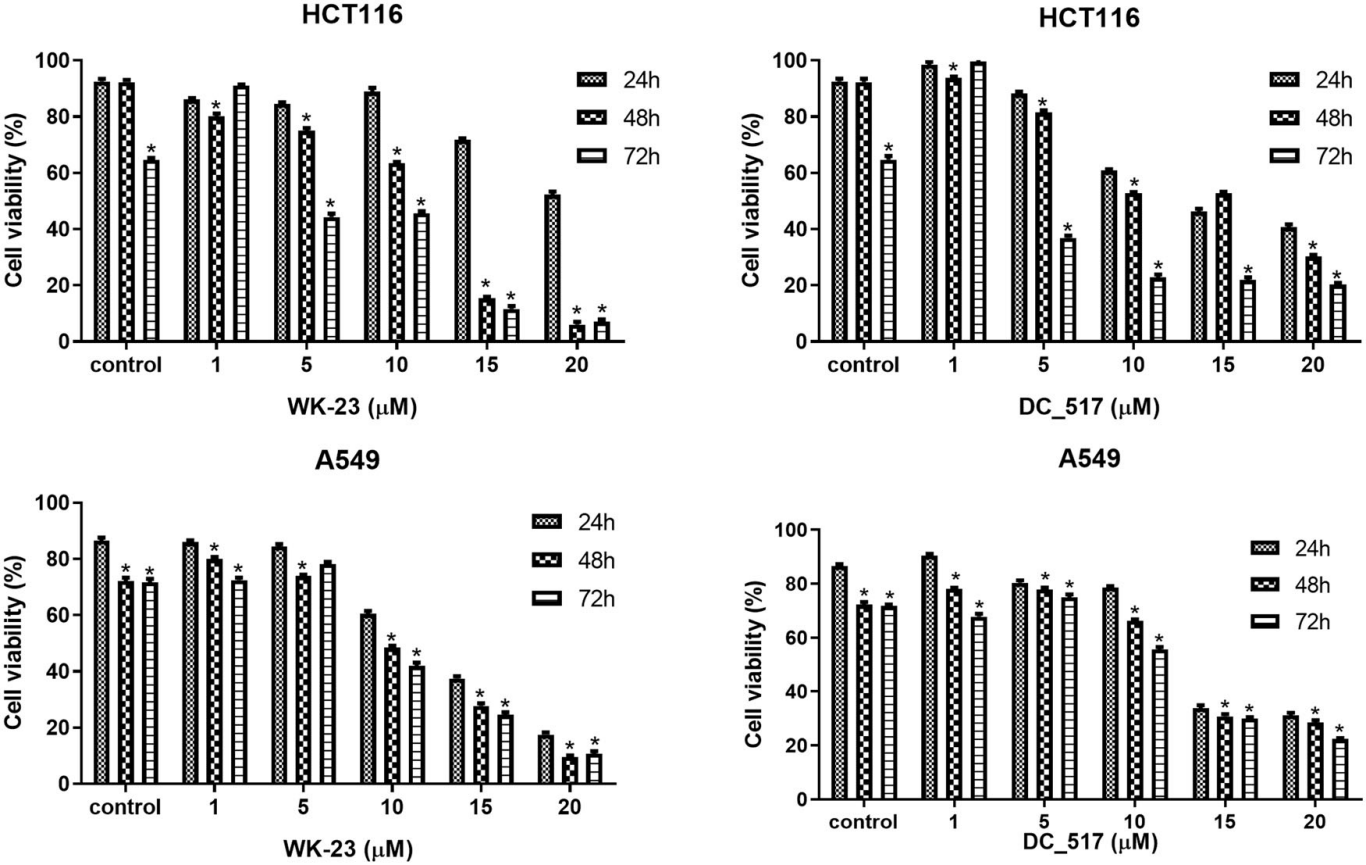
Effect of **WK-23** and **DC_517** on the viability of human colon cell lines and human lung cell lines. **WK-23** has stronger concentration dependence and time dependence than **DC_517**, which inhibited the viability of HCT116 and A549 cells in the cell viability assay. Significance between groups was analysed by one-way analysis of variance (ANOVA) using IBM SPSS software. *p < 0.05.

Collectively, compounds **WK-1**, **WK-12**, **WK-13**, **WK-19**, **WK-22**, and **WK-23** showed significant DNMT1 inhibiting activity, and we concomitantly explored the anti-proliferative effect of these most interesting compounds on cancer cell lines. As shown in Table 3, we tested these molecules in HCT116^34^ and A549^35^. Notably, **WK-22** and **WK-23** displayed the highest anti-proliferative effects in these two types of cancer cell lines, which is consistent with those of the DNMT1 inhibition assays. The results also demonstrated that **WK-22** and **WK-23** led to obvious dose-dependence and time-dependence anti-proliferation in HCT116 cancer cells (Figure 4). Moreover, in comparison with A549, generally, these compounds are more sensitive to HCT116 cells. Regarding the Pharmacokinetics study, **WK-23** exhibited an advantage in elimination half-life (*T*_1/2_ = 7.9 h) and oral bioavailability (*F*% = 37.1 ± 1.7) over other compounds. Consequently, we further explored the binding mode of **WK-23** to DNMT1.

**Table 3.** Derivatives’ Inh % at 50 μM on HCT116 and A549 cell lines

Cpd.	HCT116			A549		
	24 h	48 h	72 h	24 h	48 h	72 h
**WK-1**	90.9	88.0	94.9	88.2	78.6	87.6
**WK-12**	77.7	88.8	96.7	59.9	80.1	84.7
**WK-13**	–	86.3	93.2	–	69.2	84.1
**WK-19**	5.6	59.4	88.3	7.9	68.4	79.9
**WK-22**	77.6	91.8	97.4	64.3	98.0	95.7
**WK-23**	87.7	92.9	97.5	75.9	58.1	93.6

Values are the means of at least two independent experiments.

### 3.8. Molecular docking

**Figure 5.**
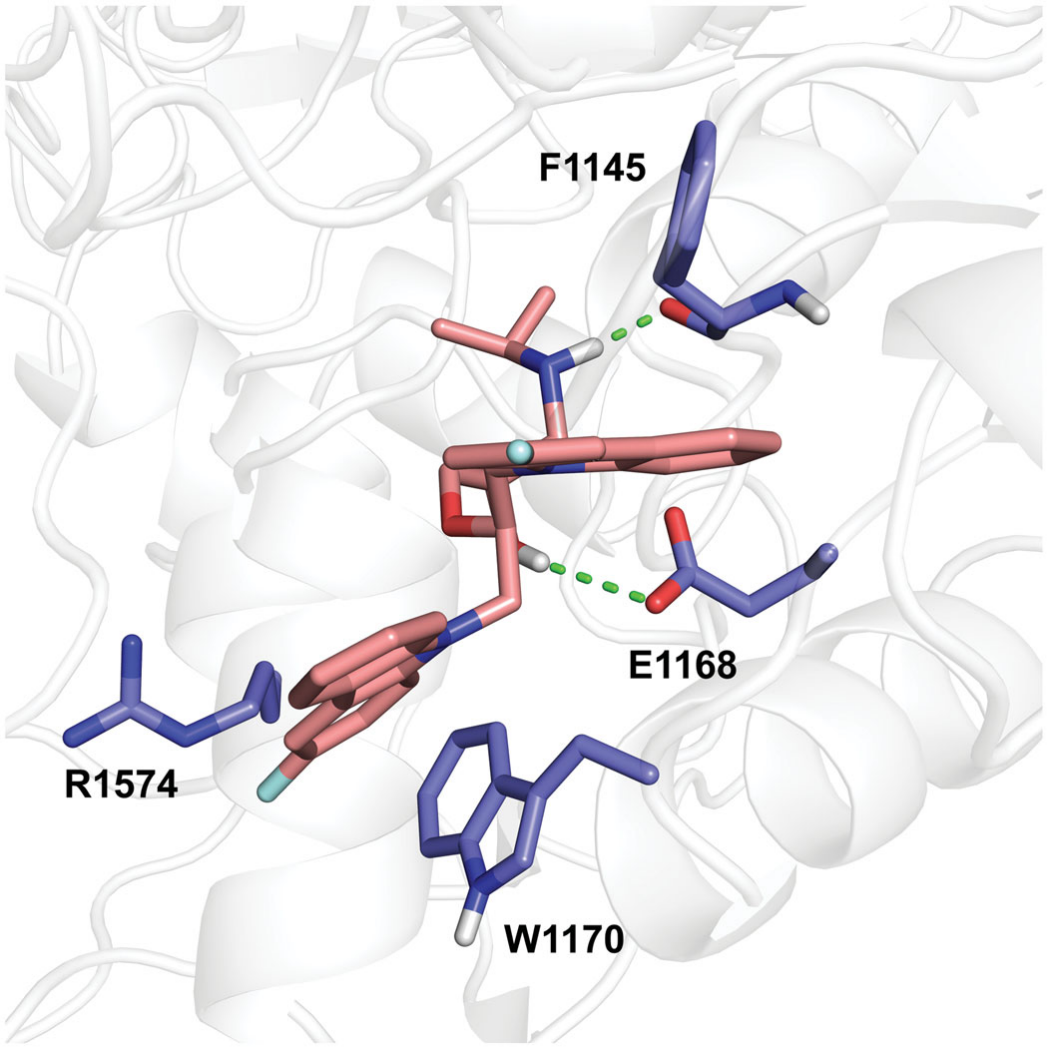
Putative binding mode of **WK-23** with DNMT1 (PDB code 4WXX). The protein (white) is shown as a cartoon. The compound (deep salmon) and the side chain of F1145, E1168, W1170, and R1574 were shown in sticks (blue).

Molecular docking was performed to explore the binding mode of **WK-23** to DNMT1. The docking procedure was validated by reproducing the SAH binding mode with a root-mean-square deviation (RMSD) of 0.965 Å (Figure S1). According to our previous study, there is no obvious difference between the enantiomers of **DC_517**, which implies the racemic of **WK-23** is applicable for molecular docking simulations with DNMT1 (PDB code 4WXX). As shown in Figure 5, the binding pattern of **WK-23** with DNMT1 is similar to that of **DC_517**^20^. One carbazolyl of **WK-23** occupied the SAM pocket, and the other carbazolyl forms cation − π interactions with R1574 and W1170. Besides, the amino group of **WK-23** interacts with the main chain of F1145, and the hydroxyl group forms hydrogen bonds with E1168. These polar interactions further stabilise the binding of the inhibitor to DNMT1.

## 4. Conclusion

In summary, a series of novel carbazole-based derivatives were designed, synthesised, and evaluated for their biological activity. The structure-activity relationship of their anti-proliferative activity was explored. Among these compounds, **WK-22** and **WK-23** displayed appreciable human DNMT1 inhibitory activity in the micromolar range (IC_50_ = 4.9 µM and 5.0 µM). Simultaneously, both **WK-22** and **WK-23** has promising anti-proliferative effect on A549 and HCT116 cell lines. In further *in vivo* pharmacokinetic study, **WK-23** displayed a better plasma exposure and prolonged elimination half-life (*T*_1/2_ = 7.9 h), especially the more acceptable oral bioavailability of (*F*% = 37.1) than **WK-22** (*F*% = 27.0). Concomitantly, the molecule docking showed the binding pattern of **WK-23** with DNMT1 is similar to that of **DC_517**, forming stable binding to DNMT1.

In conclusion, due to its favourable biological performance, compound **WK-23** warrants further assessment as a potential therapeutic agent for the treatment of human cancers.

## Author contributions

The manuscript was written through the contributions of all authors. All authors have approved the final version of the manuscript.

## Supplementary Material

Supplemental Material
