# Supplementary material for: Design, synthesis, and biological evaluation of novel carbazole derivatives as potent DNMT1 inhibitors with reasonable PK properties
Source: J Enzyme Inhib Med Chem. 2022 Jun 7;37(1):1537–55. doi: 10.1080/14756366.2022.2079640 (PMC9186373; doi:10.1080/14756366.2022.2079640)
Supplement: Supplemental Material [file IENZ_A_2079640_SM1233.pdf]

Supporting information

content

Scheme S1 3
1-((2-(1H-indol-3-yl)ethyl)amino)-3-(3-fluoro-9H-carbazol-9-yl)propan-2-ol (wk-1). 6
1-(3-fluoro-9H-carbazol-9-yl)-3-((4-fluorophenethyl)amino)propan-2-ol (WK-2). 8
4-(2-((3-(3-fluoro-9H-carbazol-9-yl)-2-hydroxypropyl)amino)ethyl)phenol (WK-3). 10
1-((3-butoxypropyl)amino)-3-(3-fluoro-9H-carbazol-9-yl)propan-2-ol (WK-5). 13
1-(3-fluoro-9H-carbazol-9-yl)-3-((2-hydroxypropyl)amino)propan-2-ol (WK-6). 14
2-((3-(3-fluoro-9H-carbazol-9-yl)-2-hydroxypropyl)amino)propan-1-ol (WK-7). 16
1-(3-fluoro-9H-carbazol-9-yl)-3-(((R)-2-phenylpropyl)amino)propan-2-ol (WK-8). 17
1-(3-fluoro-9H-carbazol-9-yl)-3-((4-fluorobenzyl)amino)propan-2-ol (WK-9). 19
1-(3-fluoro-9H-carbazol-9-yl)-3-((S)-3-(hydroxymethyl)piperazin-1-yl)propan-2-ol (WK-11). 22
1-((2-(1H-indol-3-yl)ethyl)amino)-3-(3,6-difluoro-9H-carbazol-9-yl)propan-2-ol (WK-12). 24
4-(2-((3-(3, 6-difluoro-9H-carbazol-9-yl)-2-hydroxypropyl)amino)ethyl)phenol (WK-13). 26
1-(3, 6-difluoro-9H-carbazol-9-yl)-3-((4-fluorophenethyl)amino)propan-2-ol (WK-14). 28
1-(3,6-difluoro-9H-carbazol-9-yl)-3-(phenethylamino)propan-2-ol (WK-15). 30
1-(3,6-difluoro-9H-carbazol-9-yl)-3-((2-(2-methoxyphenoxy)ethyl)amino)propan-2-ol (WK-16). 32
1-((3-butoxypropyl)amino)-3-(3,6-difluoro-9H-carbazol-9-yl)propan-2-ol (WK-17). 34
1-(3, 6-difluoro-9H-carbazol-9-yl)-3-((2-hydroxypropyl)amino)propan-2-ol (WK-18). 36
2-((3-(3,6-difluoro-9H-carbazol-9-yl)-2-hydroxypropyl)amino)propan-1-ol (WK-19). 38
1-((2-(1H-indol-3-yl)ethyl)amino)-3-(9H-pyrido[3,4-b]indol-9-yl)propan-2-ol (WK-20). 40
1-((2-(1H-indol-3-yl)ethyl)amino)-3-(9H-pyrido[2,3-b]indol-9-yl)propan-2-ol (WK-21) 42
1-((1,3-bis(3,6-difluoro-9H-carbazol-9-yl)propan-2-yl)oxy)-3-(isopropylamino)propan-2-ol (WK-22). 44
1-((1,3-bis(3-fluoro-9H-carbazol-9-yl)propan-2-yl)oxy)-3-(isopropylamino)propan-2-ol (WK-23). 46
1-((1,3-bis(3-fluoro-9H-carbazol-9-yl)propan-2-yl)oxy)-3-(cyclopropylamino)propan-2-ol (WK-24). 48
1-((1,3-bis(3-fluoro-9H-carbazol-9-yl)propan-2-yl)oxy)-3-(tert-butylamino)propan-2-ol (WK-25).

....50

(2R)-tert-butyl 4-(3-((1-(3,6-difluoro-9H-carbazol-9-yl)-3-(3-fluoro-9H-carbazol-9-yl)propan-2-yl)oxy)-2-hydroxypropyl)-2-(hydroxymethyl)piperazine-1-carboxylate (WK-26). 52

1-((1-(3,6-difluoro-9H-carbazol-9-yl)-3-(3-fluoro-9H-carbazol-9-yl)propan-2-yl)oxy)-3-((R)-3-(hydroxymethyl)piperazin-1-yl)propan-2-ol (WK-27). 54

1-((2-(1H-indol-3-yl)ethyl)amino)-3-((1-(3,6-difluoro-9H-carbazol-9-yl)-3-(3-fluoro-9H-carbazol-9-yl)propan-2-yl)oxy)propan-2-ol (WK-28). 56

1-((3-butoxypropyl)amino)-3-((1-(3,6-difluoro-9H-carbazol-9-yl)-3-(3-fluoro-9H-carbazol-9-yl)propan-2-yl)oxy)propan-2-ol (WK-29)..58

1-((1-(3,6-difluoro-9H-carbazol-9-yl)-3-(3-fluoro-9H-carbazol-9-yl)propan-2-yl)oxy)-3-isopropoxypropan-2-ol (WK-30). 60

Relative selectivity index 62

Docking validation..62

Scheme S1

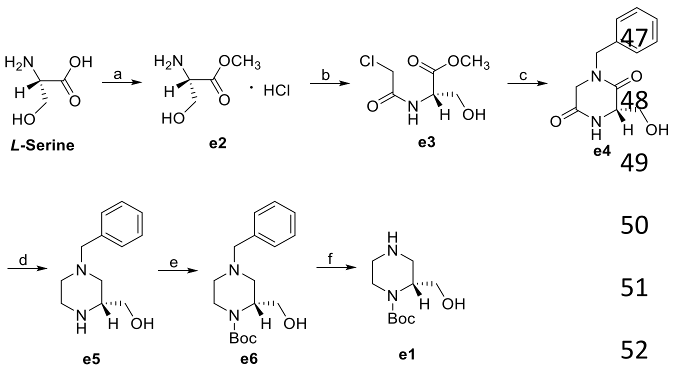

Reagents and conditions: a. $SOCl_2$, CH_3OH, r.t. ; b. $ClCH_2COCl$, $NaHCO_3$, DCM, r.t. , 6h; c. Phenylmethanamine, TEA, CH_3OH, reflux; d. $LiAlH_4$, anhyd.THF, N_2, reflux, 4h; e. $(Boc)_2O$, TEA, DCM, r.t. ; f. Pd/C, $Pd(OH)_2/C$; CH_3OH, H_2, r.t. , 5h.

(S)-methyl 2-amino-3-hydroxypropanoate hydrochloride (**e2**)

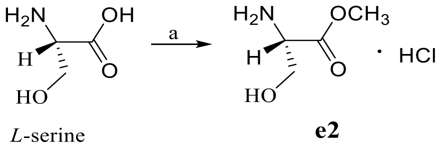

A solution of L-serine (50.00 g, 476.0 mmol) in methanol (500 ml) was stirred under ice bath, then $SOCl_2$ (226.67 g, 1904.8 mmol) was added dropwise and the resultant mixture was stirred at room temperature overnight. The solvent was concentrated in vacuo to afford white solid **e2** (71.86 g), which was used to next step without further purification.

(S)-methyl 2-(2-chloroacetamido)-3-hydroxypropanoate (**e3**)

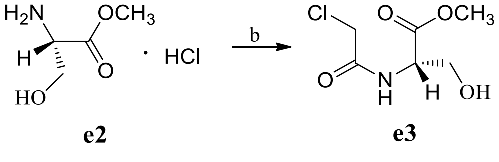

A solution of **e2** (71.86 g, 464.0 mmol) in H_2O (250 mL) was stirred under salt-ice bath, then $NaHCO_3$ (93.54 g, 1113.6 mmol) was added and stirred for 5 min. Another solution of $ClCH_2COCl$ (57.68 g, 510.4 mmol) in dichloromethane (200 mL) was added dropwise and the mixture was stirred at room temperature for 6 h. The mixture was treated with dichloromethane and water. The organic layer was washed

with brine, dried over anhydrous Na_2SO_4, and concentrated in vacuo. Finally afforded (*S*)-methyl 2-(2-chloroacetamido)-3-hydroxypropanoate (**e3**) as a canary yellow liquid, 80.37 g (yield 89%), which was used to next step without further purification.

(S)-1-benzyl-3-(hydroxymethyl) piperazine-2,5-dione (**e4**)

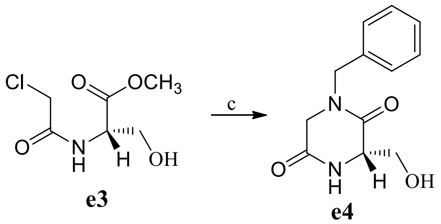

e3 **e4**

A solution of **e3** (36.00 g, 185.0 mmol) in methanol (180 mL) was stirred under ice bath, then Et_3N (56.06 g, 555.0 mmol) was added and stirred for 5 min. Benzylamine (23.75 g, 222.0 mmol) was added in portions and the mixture was reflux overnight. The mixture was treated with 40 mL ethyl acetate during -30°C cold storage, and filtrated to get a white precipitated (31.50 g). All of the white solid was dissolved in MeOH: EA=150: 90 and recrystallized under 4°C after stirring 30 min, to afford a white solid, 24.00 g (yield 55%).

(R)-(4-benzylpiperazin-2-yl) methanol (**e5**)

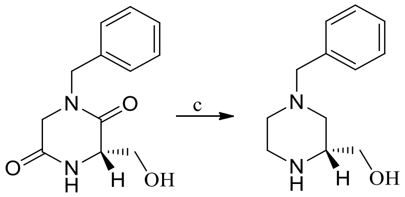

e4 **e5**

A solution of $LiAlH_4$ (12.35 g, 324.9 mmol) in extra dry THF (200 mL) was stirred under ice bath, then **e4** (20.00 g, 85.5 mmol) was added partially and stirred for 30 min under N_2 atmosphere at room temperature. Then the reaction was heated to reflux for 4 h. After the complete consumption of compound **e4**, the reaction was quenched with water and filtrated. The aqueous layer was extracted by dichloromethane (30 mL*3), and the combined organics was washed with saturated aqueous NaCl, dried over anhydrous Na_2SO_4, filtered, and concentrated in vacuo, to afford *(R)-(4-benzylpiperazin-2-yl) methanol* (**e5**), as a yellow liquid, 17.85 g (yield 27%).

(R)-tert-butyl 4-benzyl-2-(hydroxymethyl) piperazine-1-carboxylate (**e6**)

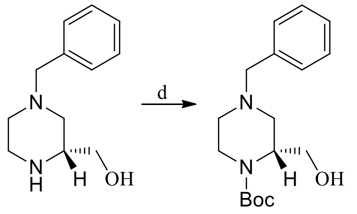

e5 **e6**

A mixture of intermediate compound **e5** (17.85 g, 87.0 mmol, 1 equiv) and triethylamine (9.67 g, 95.7 mmol, 1.1 equiv) dissolved in 75 mL DCM, was introduced into a 250 mL flask. Then a solution of $(Boc)_2O$ (20.86 g, 95.7 mmol) in DCM (75 mL) was added dropwise. The mixture was stirred at room temperature overnight, and monitored by TLC (PE: EA = 5:1) until compound **e5** was completely consumed. The reaction was quenched with water. The aqueous layer was extracted by dichloromethane (100 mL*2), and the combined organics was washed with saturated aqueous NaCl, dried over anhydrous Na_2SO_4, filtered, and concentrated in vacuo. The residue was purified by chromatography (SiO_2, PE: EA= 10:1- 2:1) to yield compound **e6** as a faint yellow liquid (16.60 g, 62%).

(R)-tert-butyl 2-(hydroxymethyl) piperazine-1-carboxylate (**e1**)

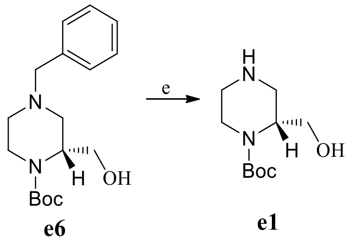

e6 **e1**

To the solution of compound **e6** (16.30 g, 53.3 mmol) in 150 mL of methanol were added Pd/C (1.6 g) and $Pd(OH)_2$ (0.8 g), then added 8 drops of acetic acid. The nitrogen was purged and replaced with a H_2 balloon. Once complete, the reaction was filtered and concentrated in vacuo, to afford (*R*)-tert-butyl 2-(hydroxymethyl) piperazine-1-carboxylate (**e1**) as a yellow white wax-like solid, 10.60 g, 92% yield. The characteristics were as follows: m.p. 85-87°C. HRMS-ESI calcd for $C_{10}H_{21}N_2O_3$ $[M+H]^+$ 217.1552, found 217.1553.

^1H NMR (600 MHz, Chloroform-d) δ 4.00 (s, 1H), 3.84 (d, *J* = 5.4 Hz, 3H), 3.17 (d, *J* = 12.1 Hz, 2H), 3.08 (s, 2H), 2.95 (d, *J* = 11.1 Hz, 1H), 2.85 (dd, *J* = 12.2, 4.2 Hz, 1H), 2.69 (td, *J* = 12.1, 3.7 Hz, 1H), 1.43 (s, 9H).

^{13}C NMR (151 MHz, Chloroform-d) δ 155.50, 80.11, 63.80, 51.23, 46.99, 45.63, 28.49.

^{1}H NMR and ^{13}C NMR spectra of all target compounds

***1-((2-(1H-indol-3-yl)ethyl)amino)-3-(3-fluoro-9H-carbazol-9-yl)propan-2-ol* (wk-1).**

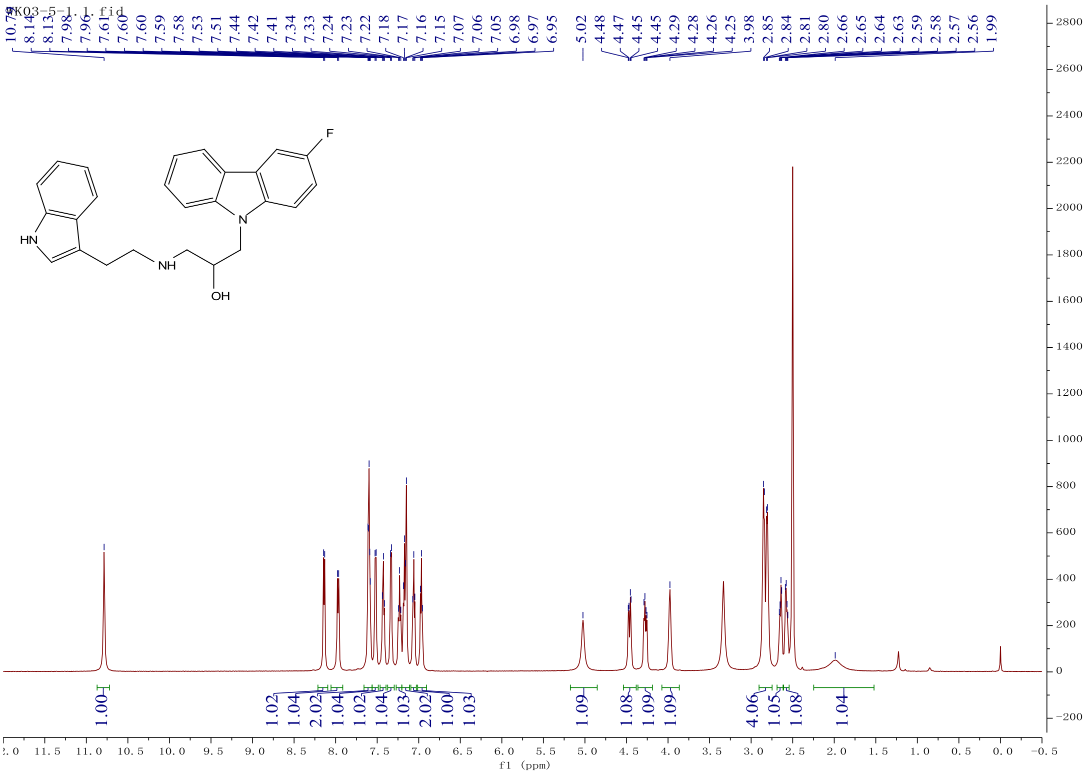

WK-1 ^{1}H NMR

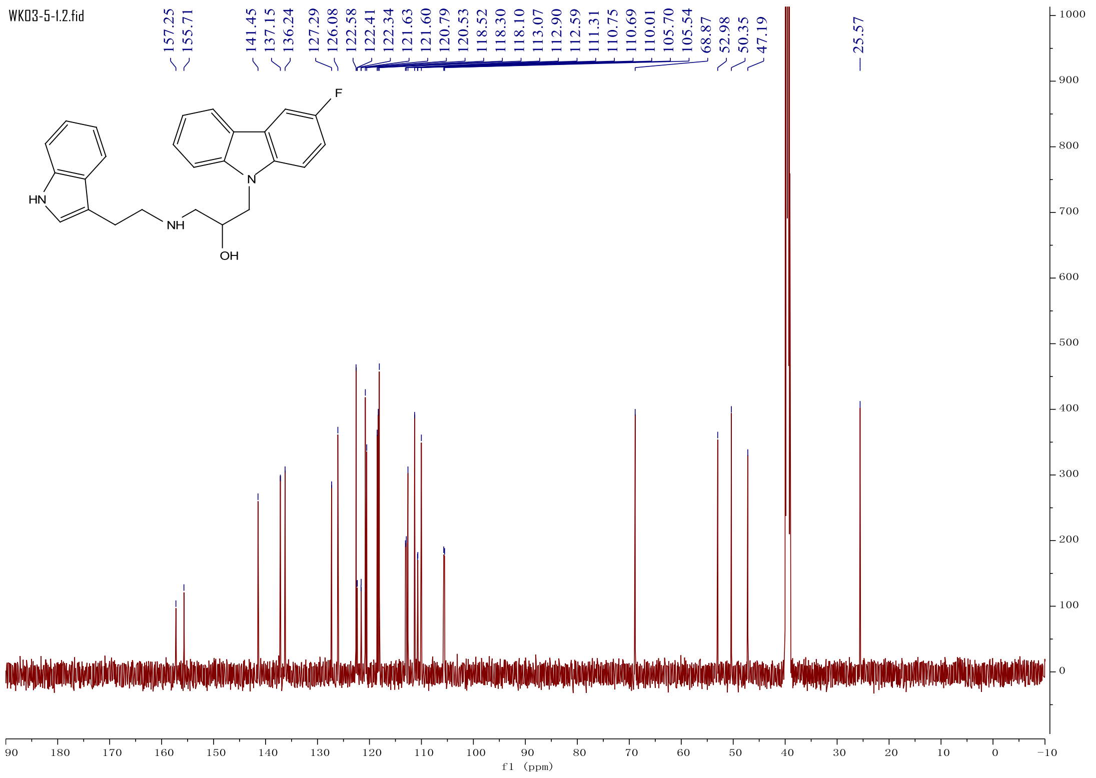

WK-2 ^{13}C NMR

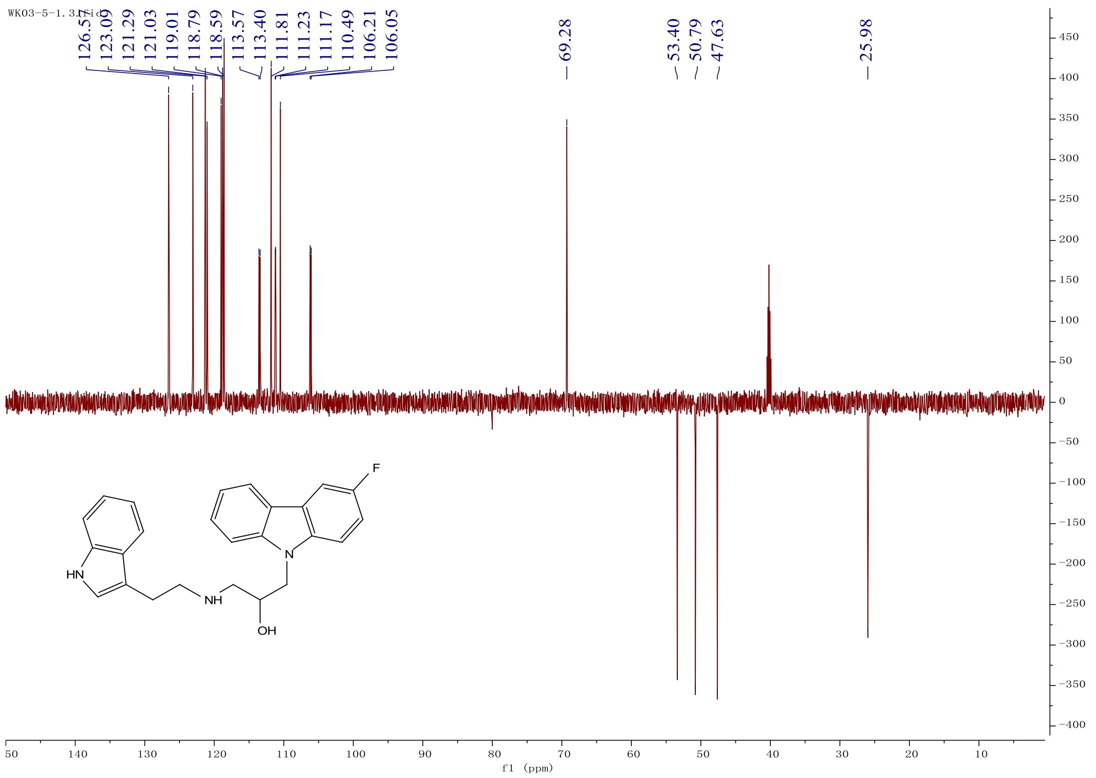

WK-2 Dept 135

***1-(3-fluoro-9H-carbazol-9-yl)-3-((4-fluorophenethyl)amino)propan-2-ol* (WK-2).**

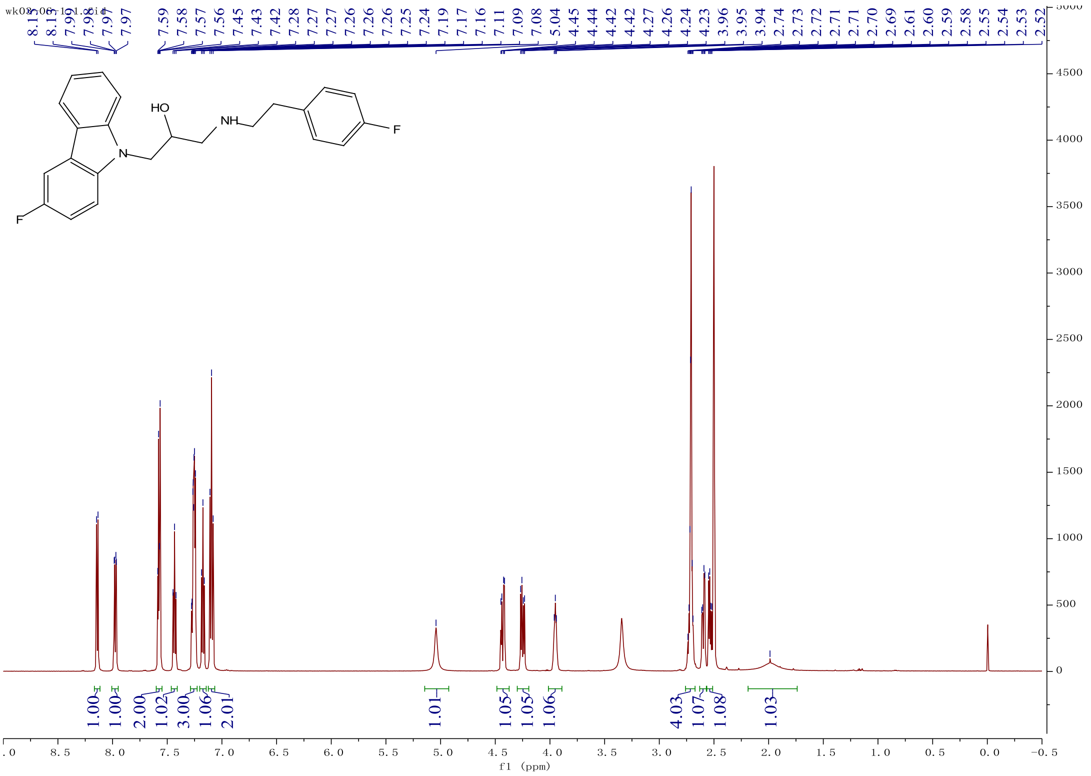

WK-2 ^{1}H NMR

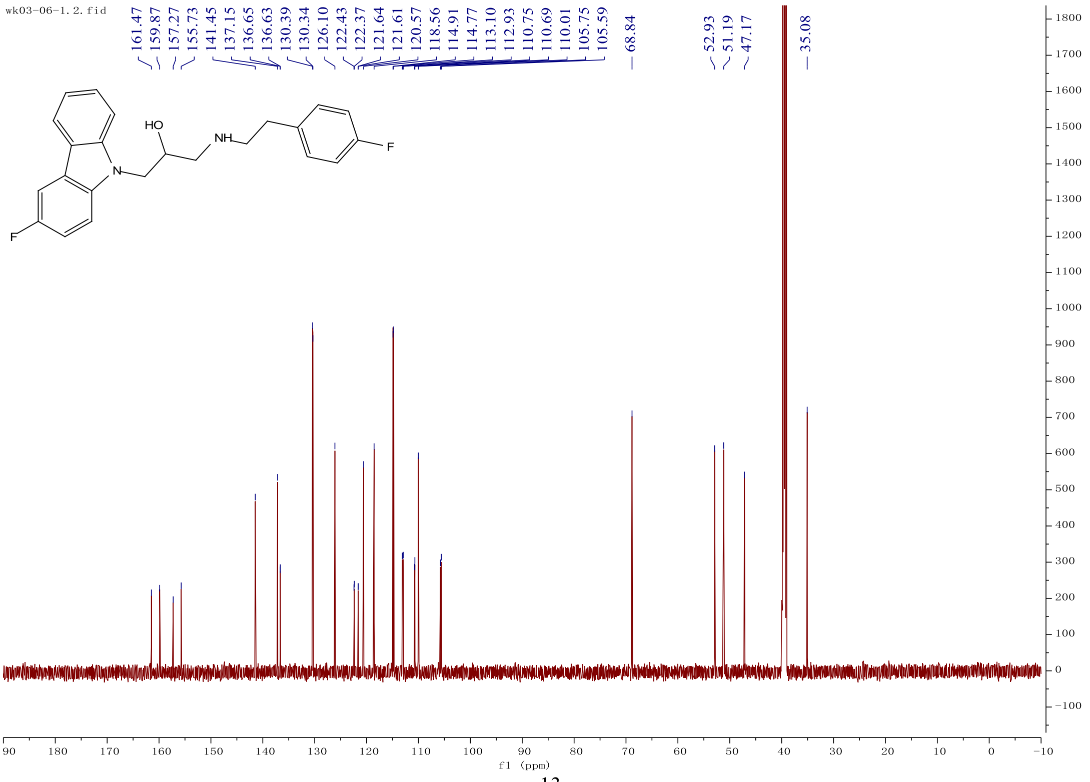

WK-2 ^{13}C NMR

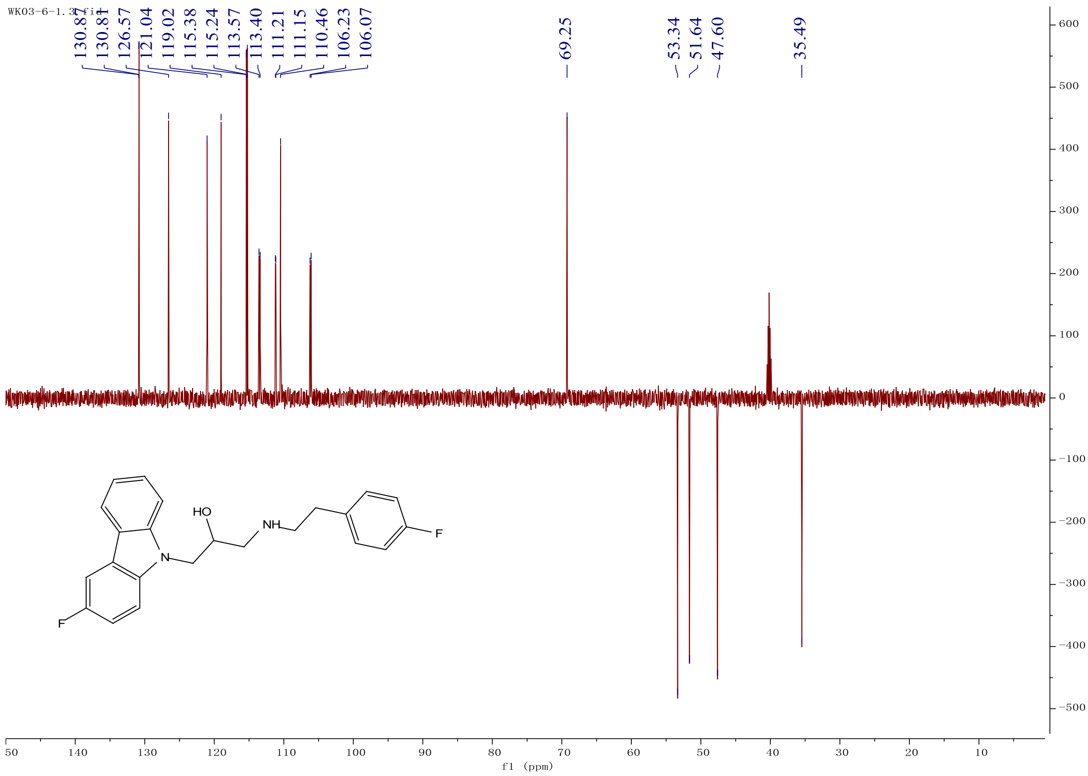
WK03-6-1.3.fid
130.87
130.81
126.57
121.04
119.02
115.38
115.24
113.57
113.40
111.21
111.15
110.46
106.23
106.07
69.25
53.34
51.64
47.60
35.49
HO
NH
N
F
F
f1 (ppm)

WK-2 Dept 135

***4-(2-((3-(3-fluoro-9H-carbazol-9-yl)-2-hydroxypropyl)amino)ethyl)phenol* (WK-3).**

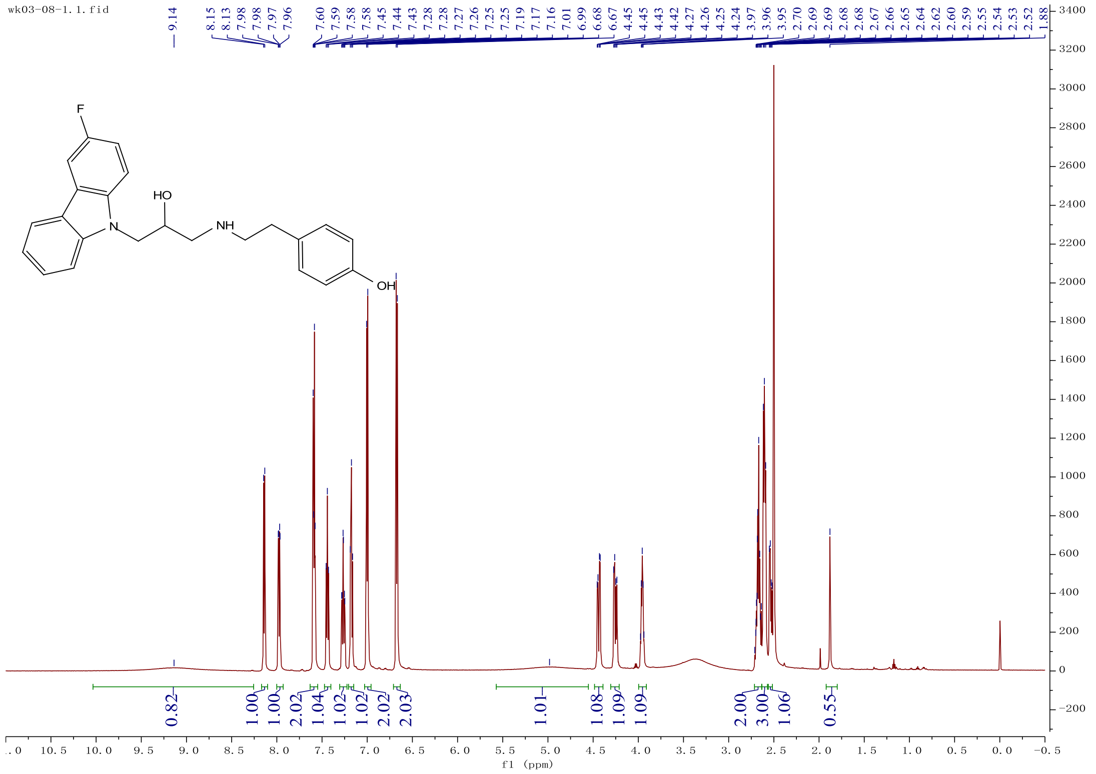

WK-3 ^{1}H NMR

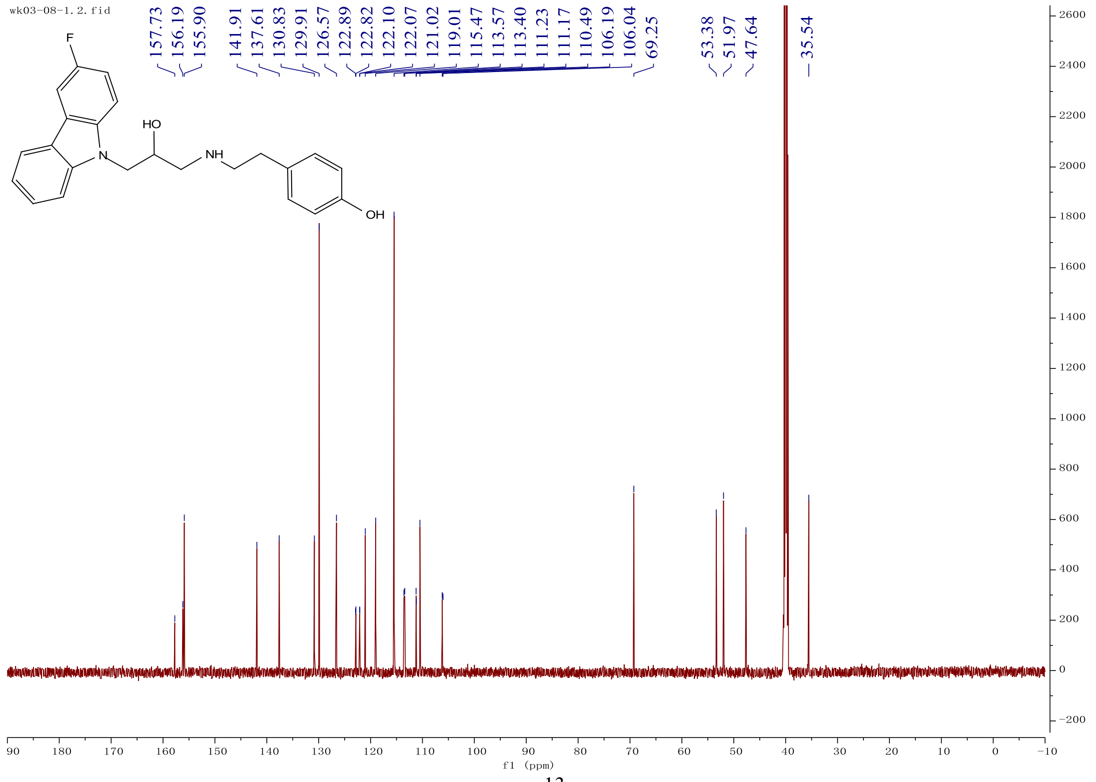

WK-3 ^{13}C NMR

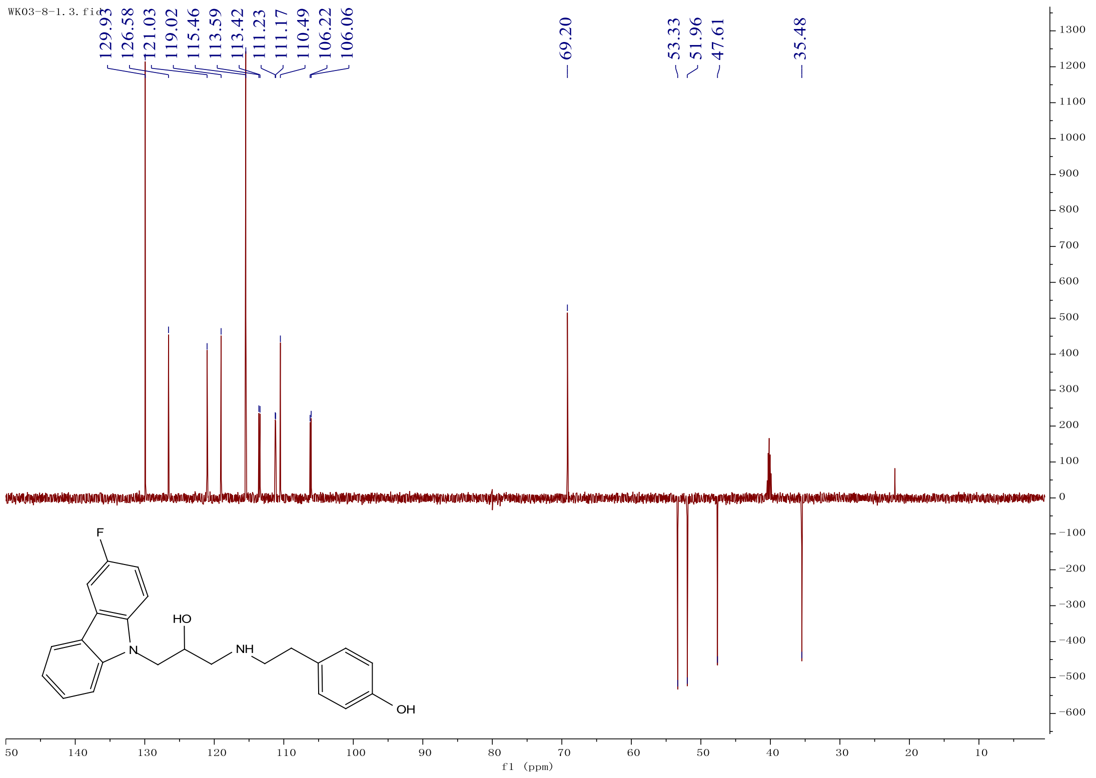

WK-3 Dept 135

1-(3-fluoro-9H-carbazol-9-yl)-3-((2-(2-methoxyphenoxy)ethyl)amino)propan-2-ol (**WK-4**).

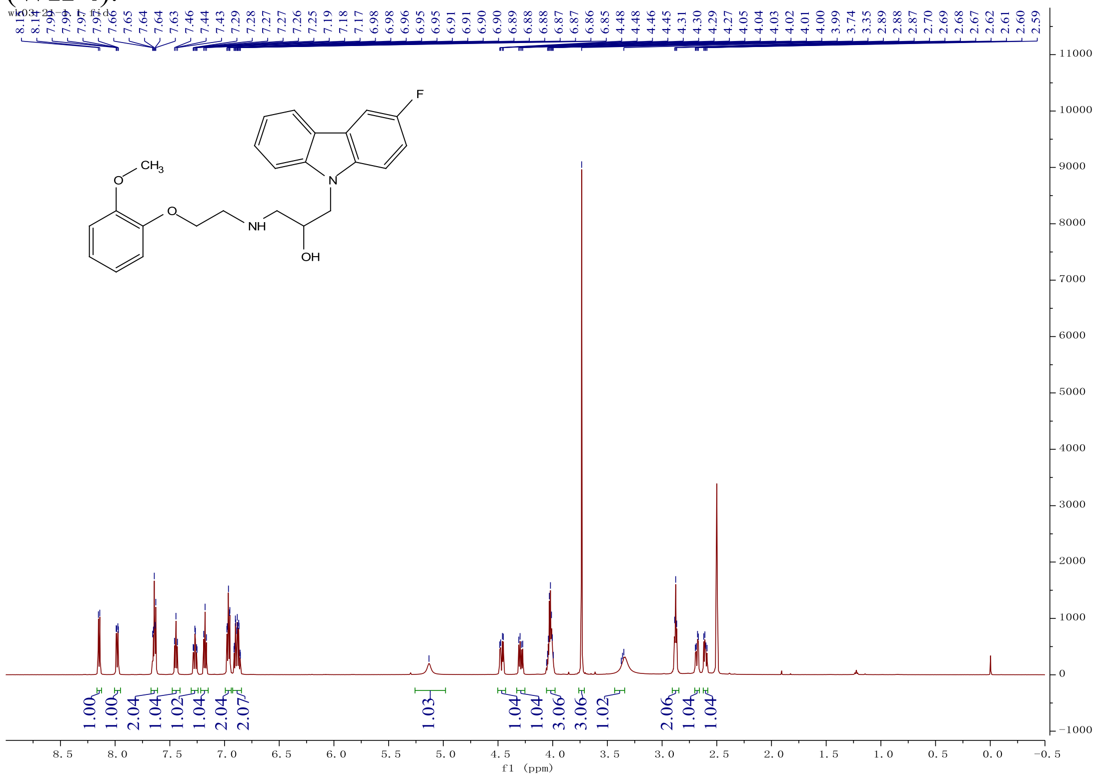

WK-4 ^{1}H NMR

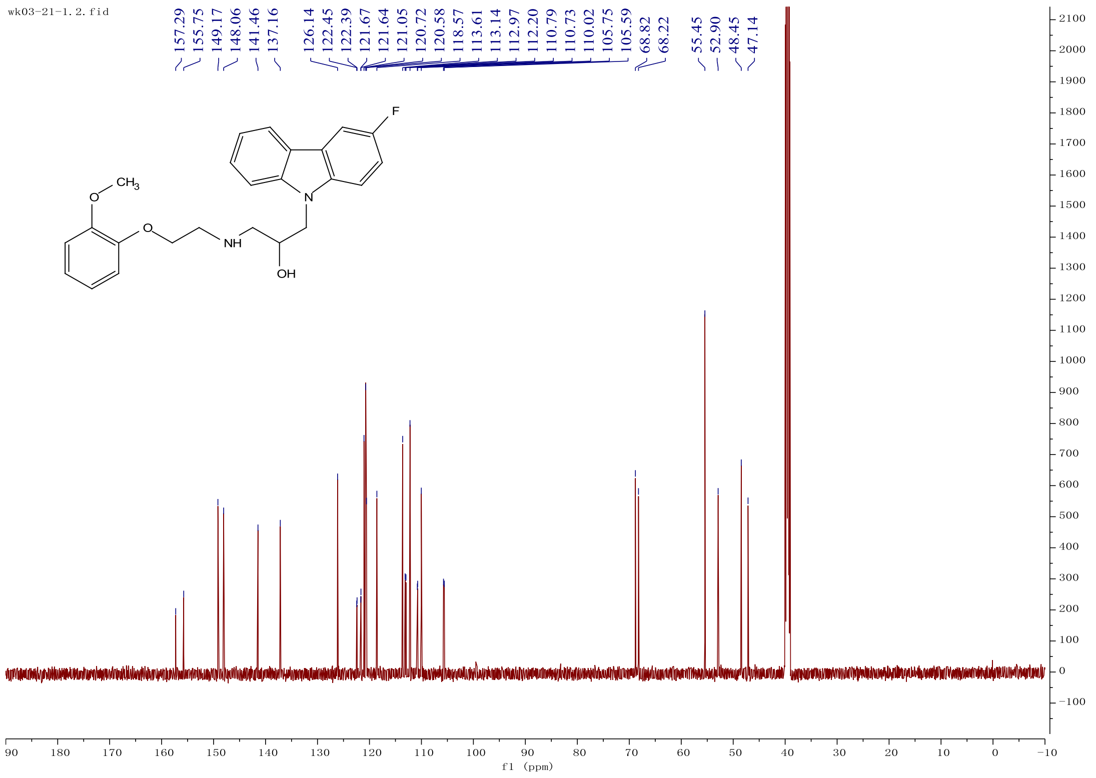

WK-4 ^{13}C NMR

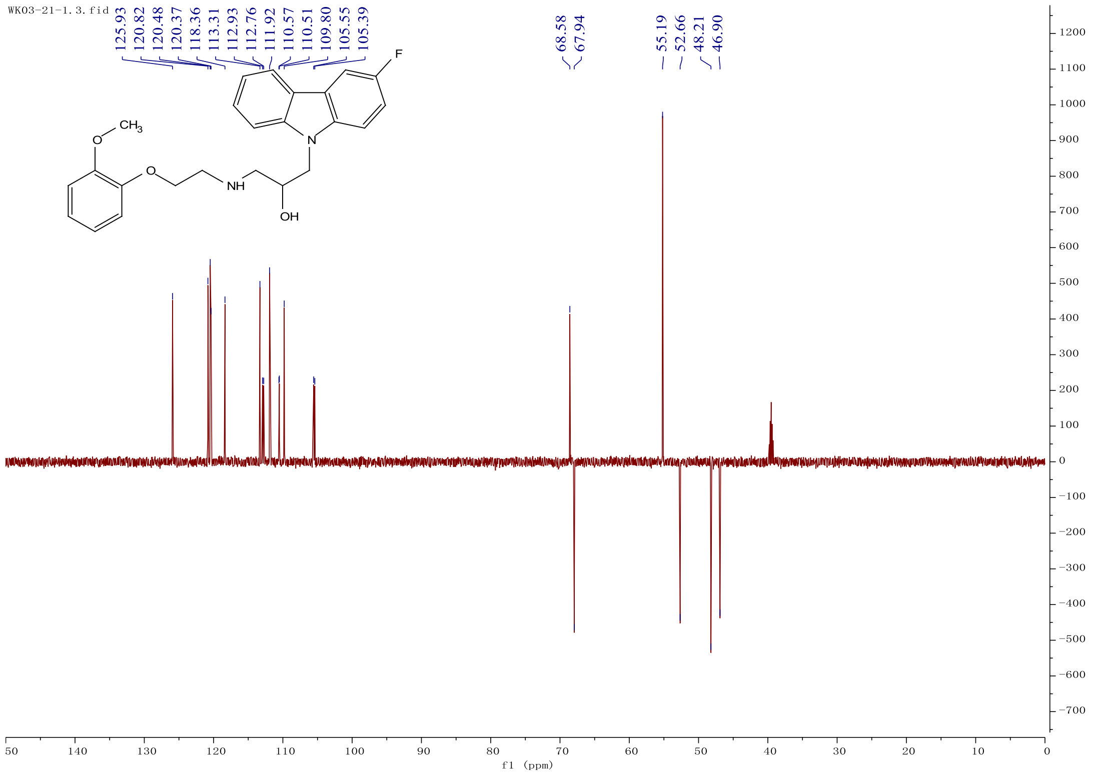

WK-4 Dept 135

***1-((3-butoxypropyl)amino)-3-(3-fluoro-9H-carbazol-9-yl)propan-2-ol* (WK-5).**

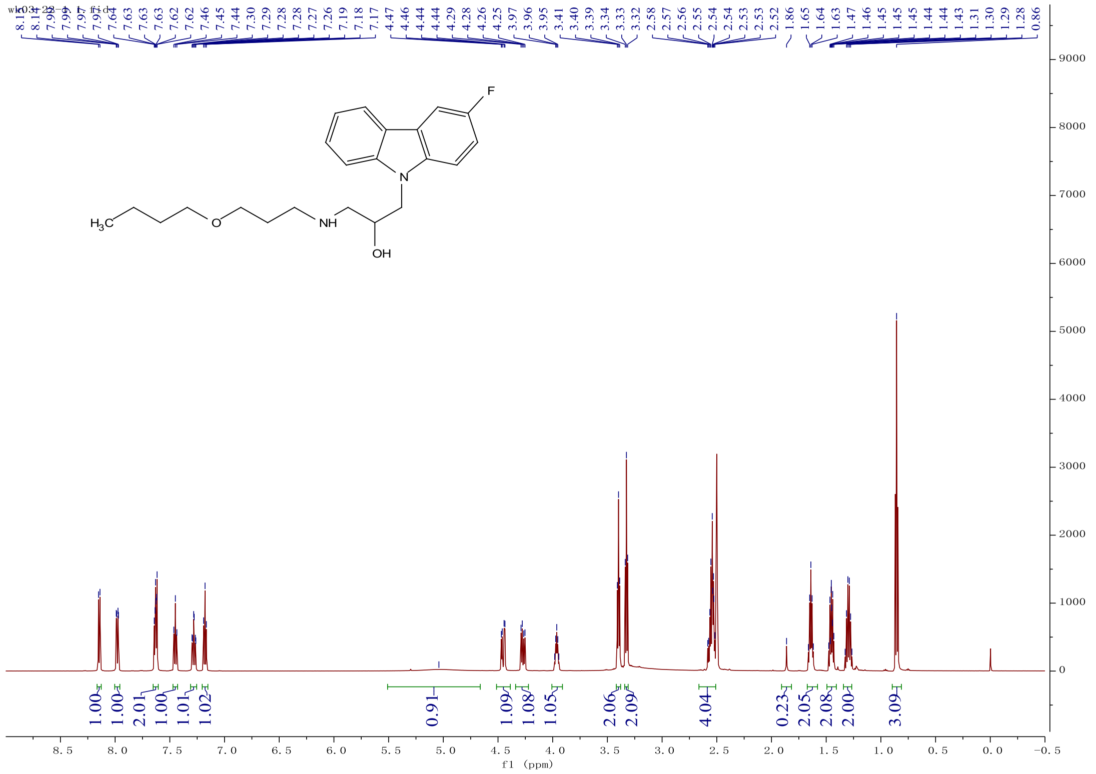

WK-5 ^{1}H NMR

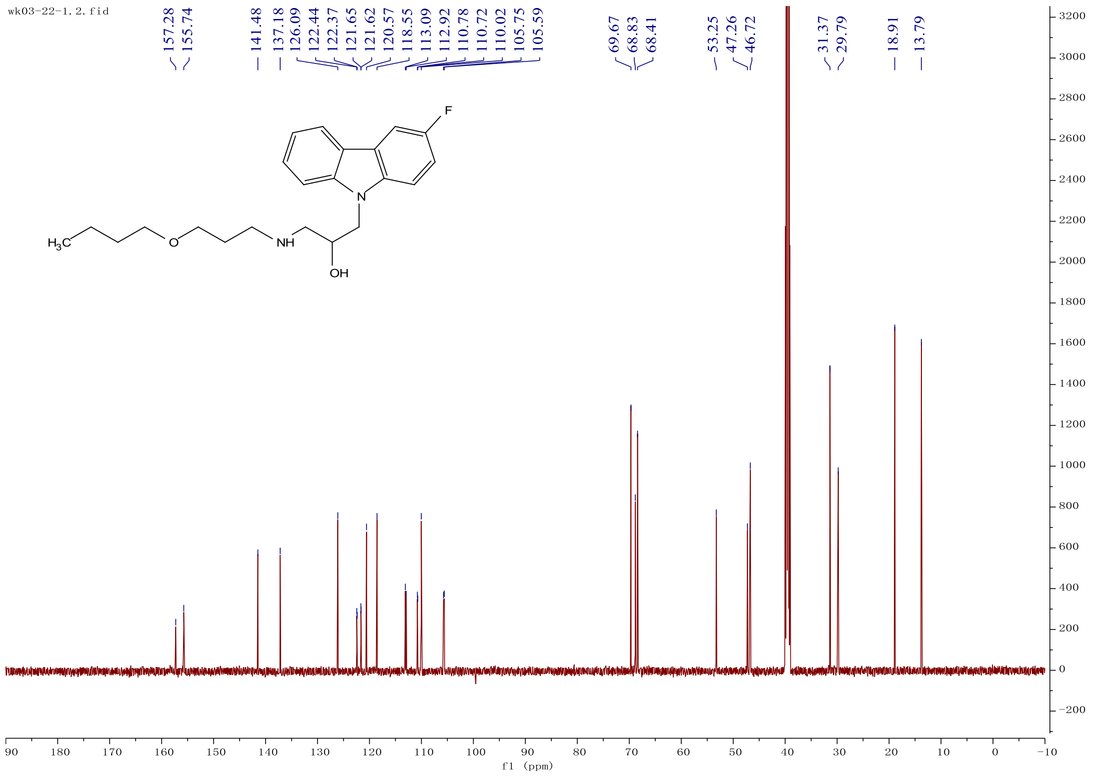

WK-5 ^{13}C NMR

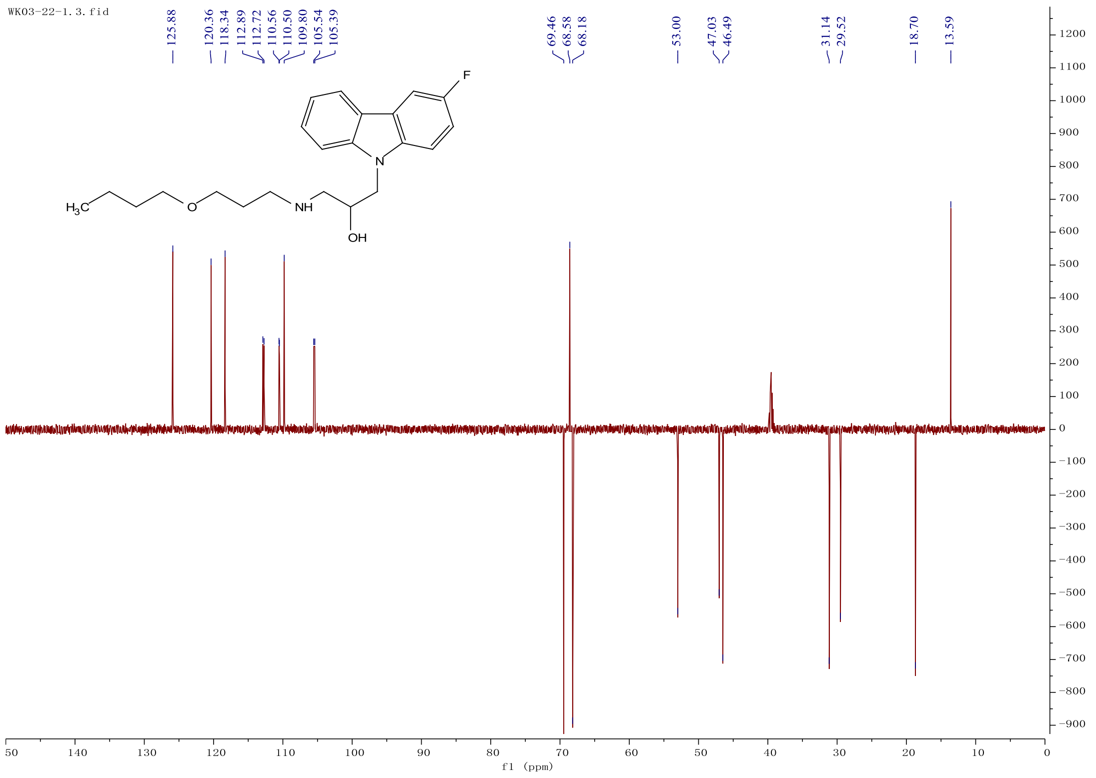

WK-5 Dept 135

1-(3-fluoro-9H-carbazol-9-yl)-3-((2-hydroxypropyl)amino)propan-2-ol **(WK-6).**

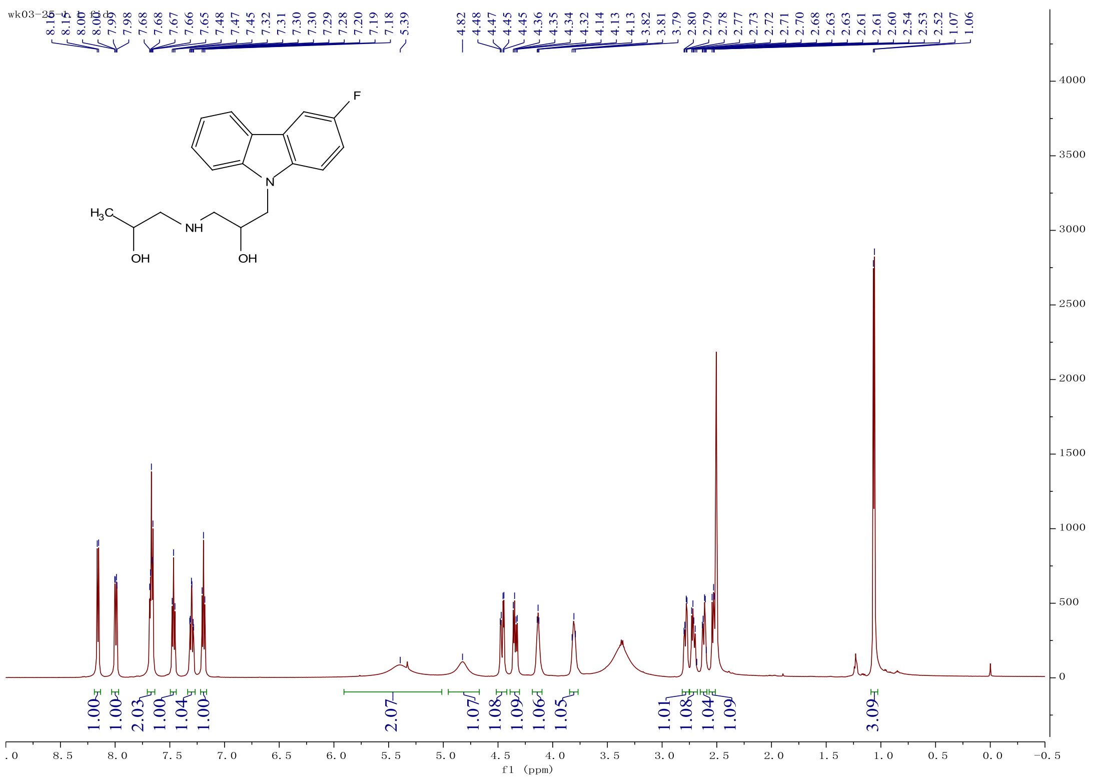

WK-6 ^{1}H NMR

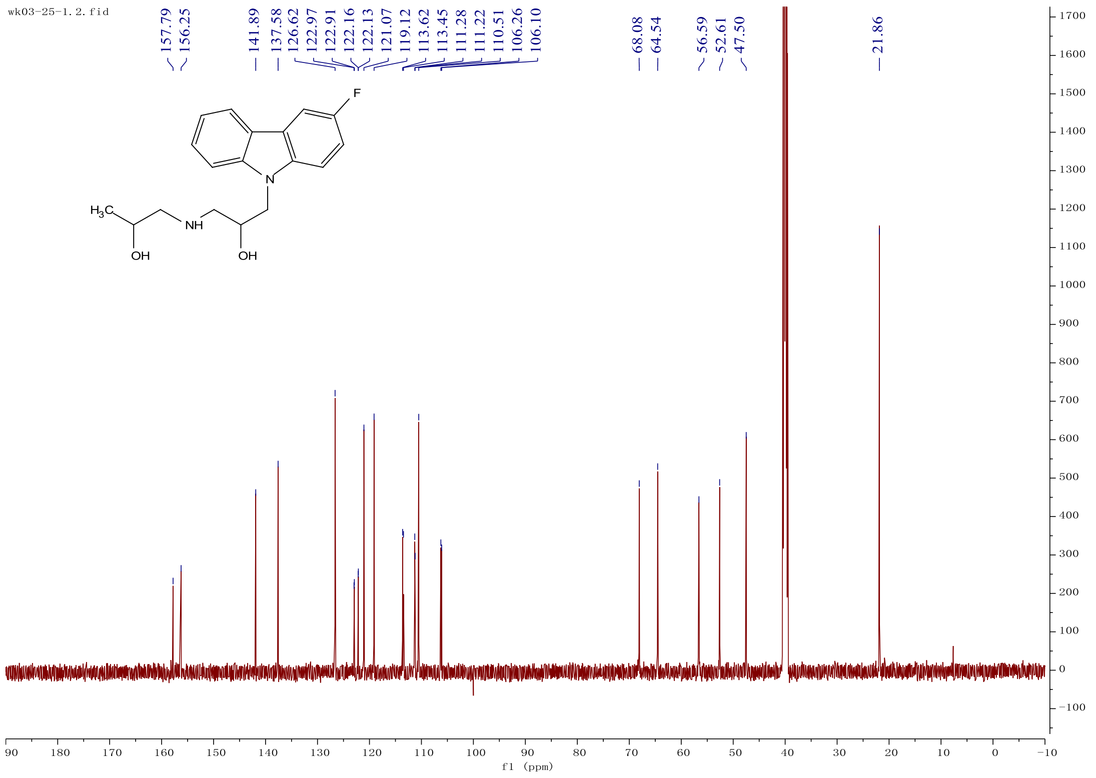

WK-6 ^{13}C NMR

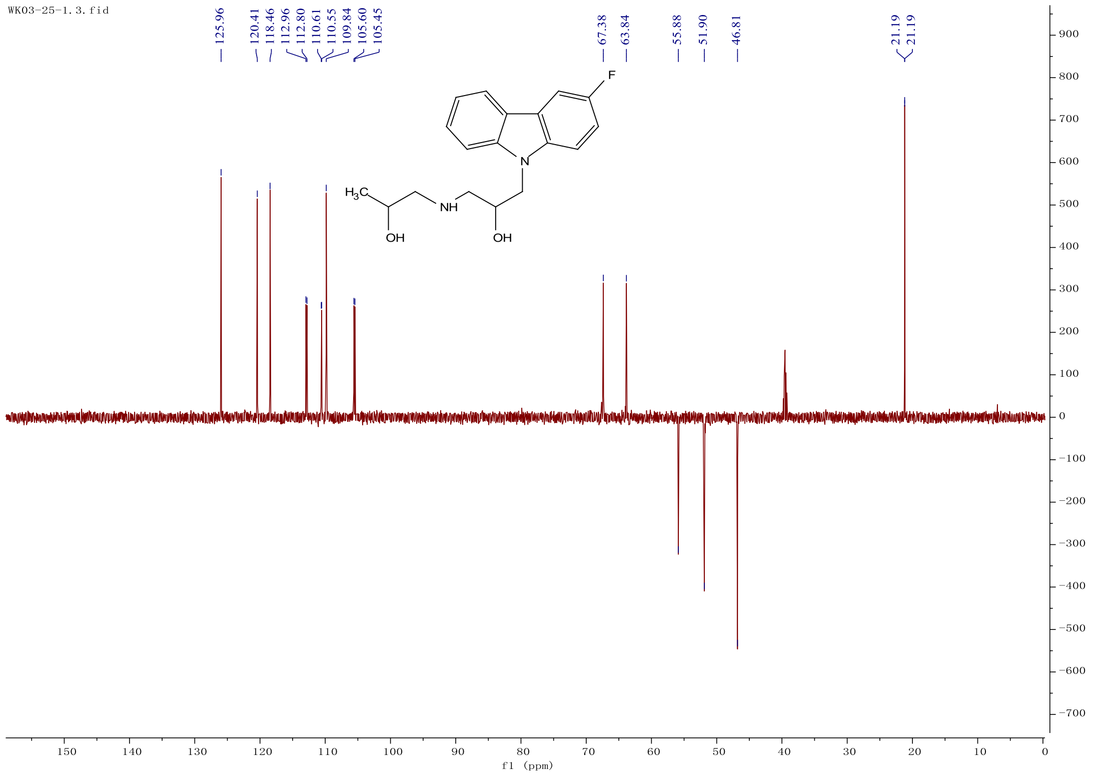

WK-6 Dept 135

2-((3-(3-fluoro-9H-carbazol-9-yl)-2-hydroxypropyl)amino)propan-1-ol **(WK-7).**

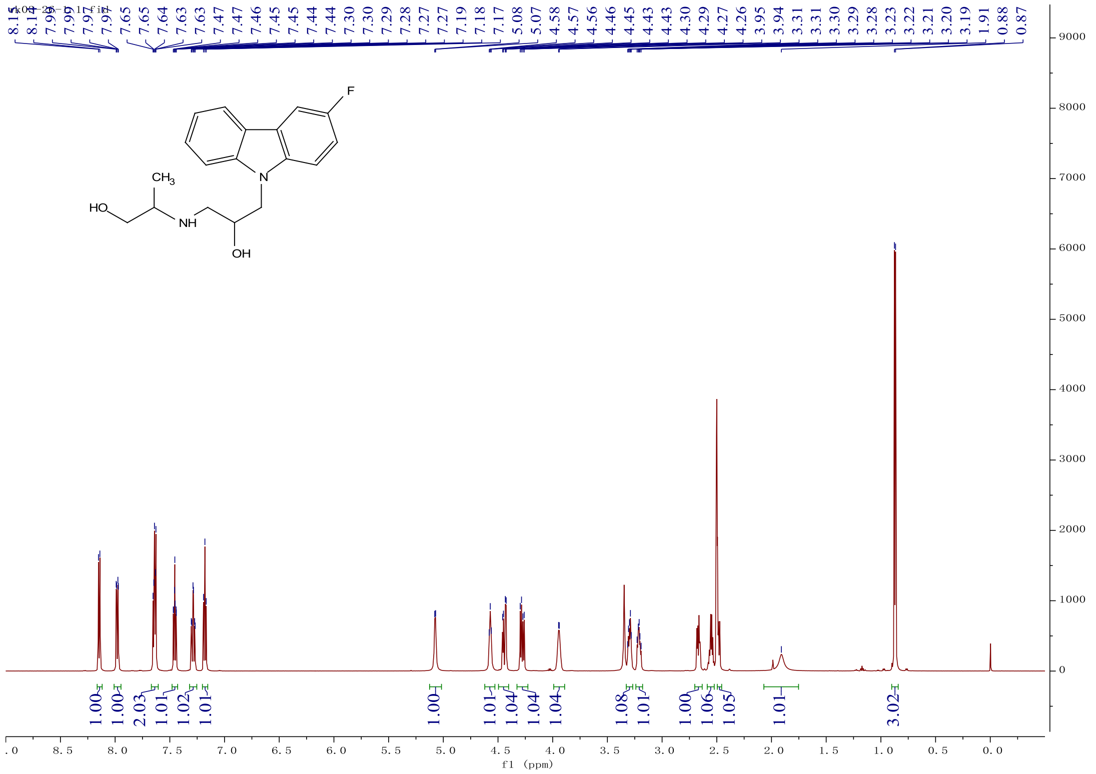

WK-7 ^{1}H NMR

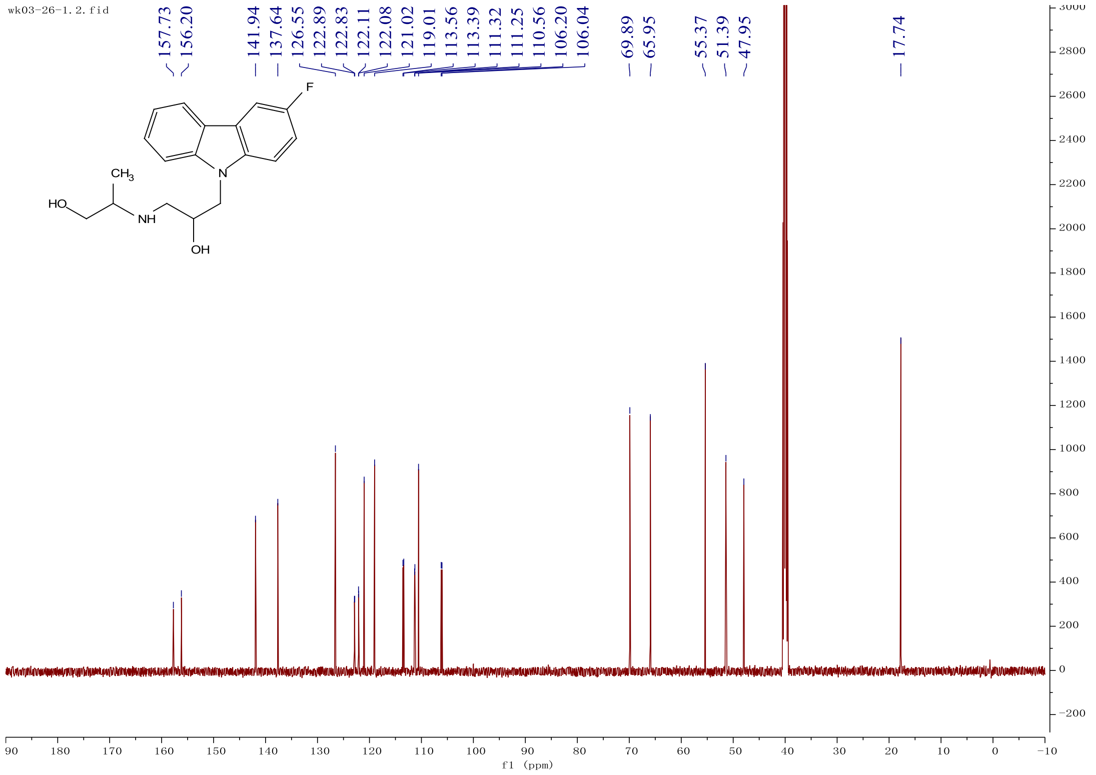

WK-7 ^{13}C NMR

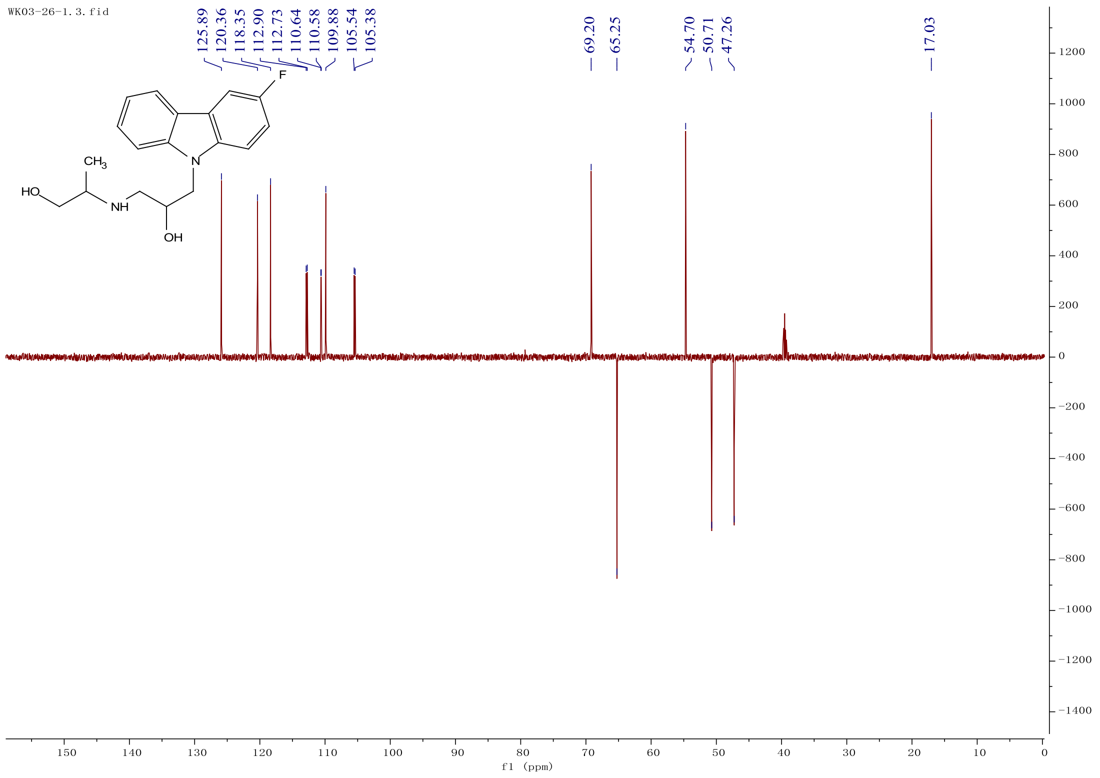

WK-7 Dept 135

***1-(3-fluoro-9H-carbazol-9-yl)-3-(((R)-2-phenylpropyl)amino)propan-2-ol* (WK-8).**

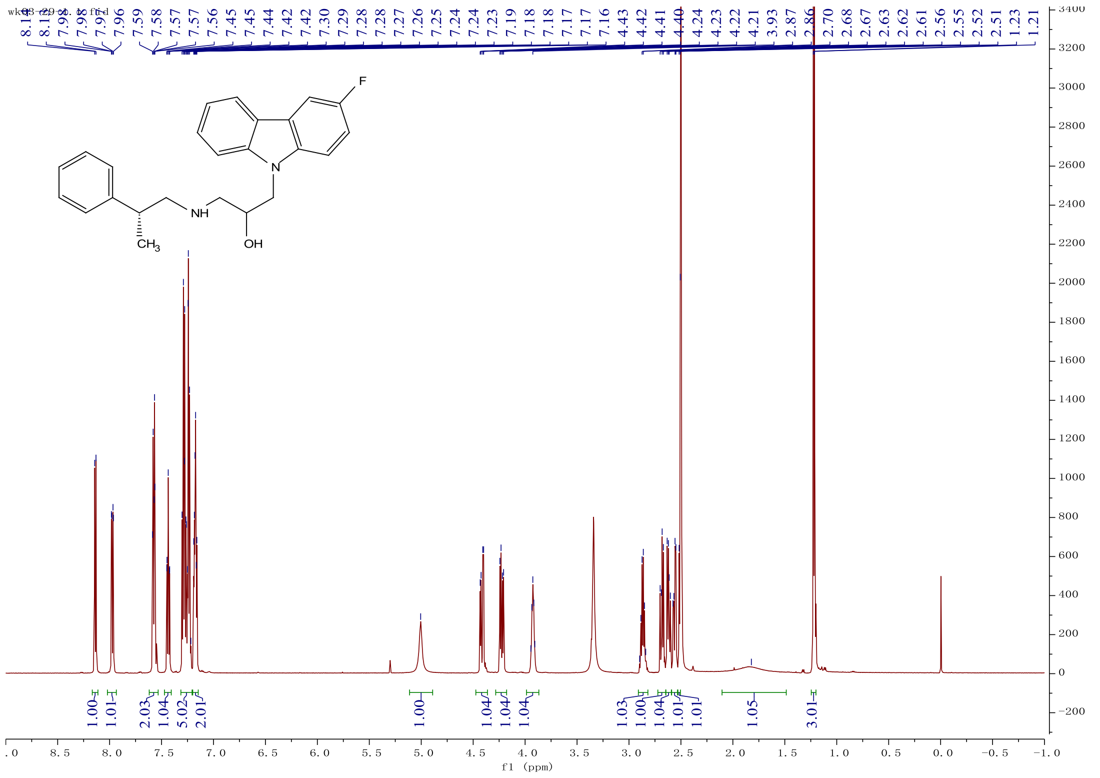

WK-8 ^{1}H NMR

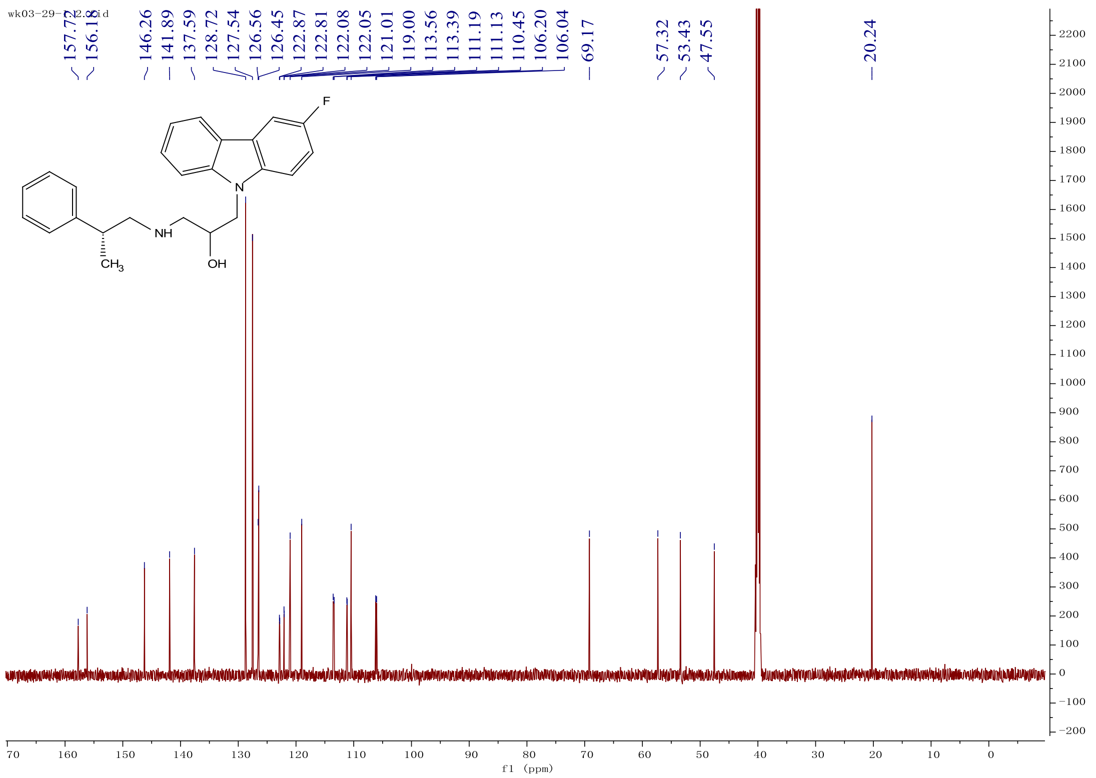

WK-8 ^{13}C NMR

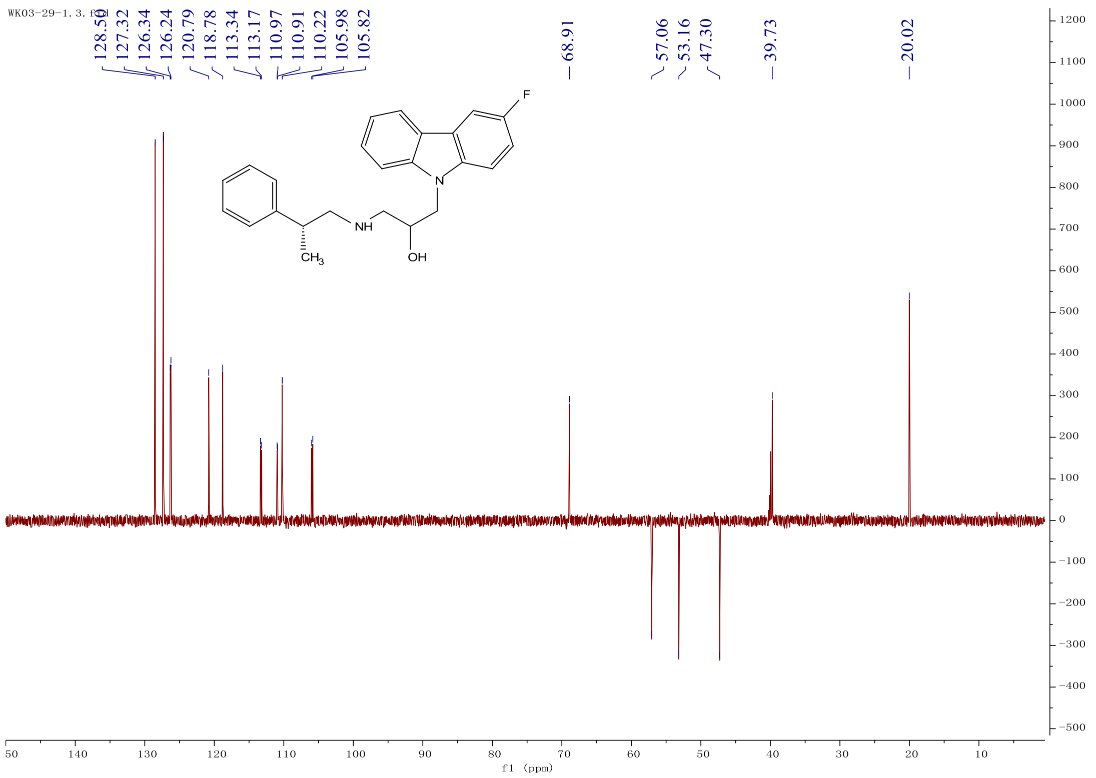

WK-8 Dept 135

***1-(3-fluoro-9H-carbazol-9-yl)-3-((4-fluorobenzyl)amino)propan-2-ol* (WK-9).**

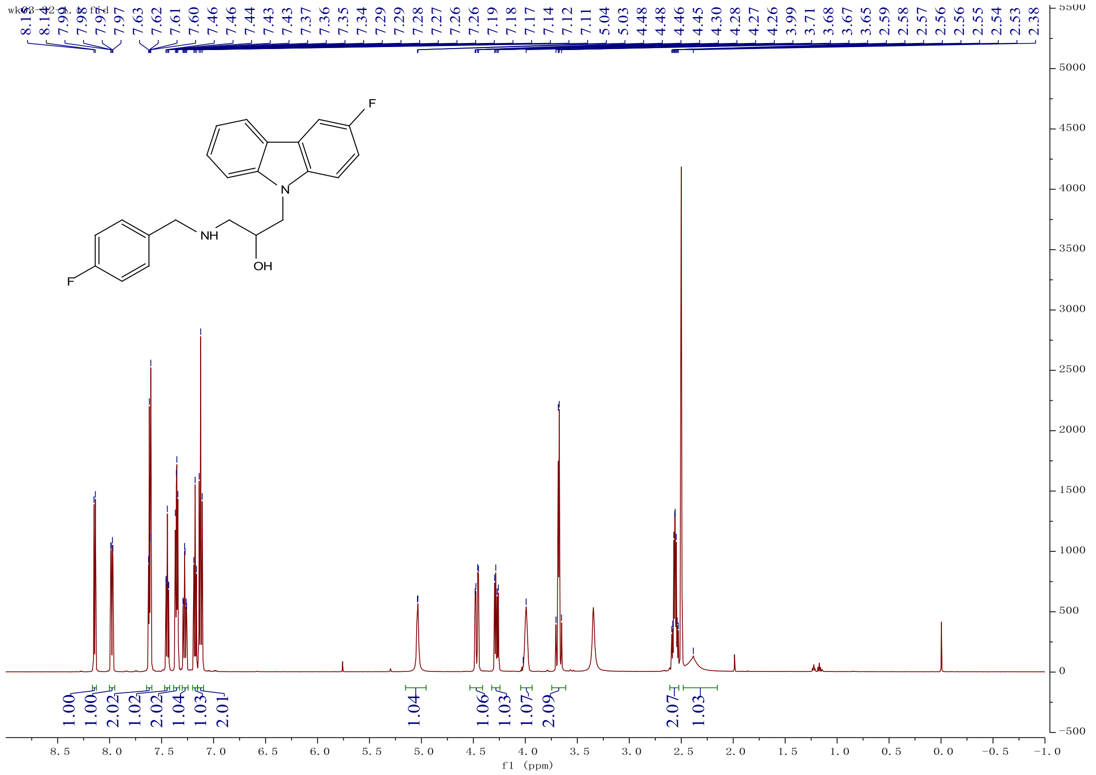

WK-9 ^{1}H NMR

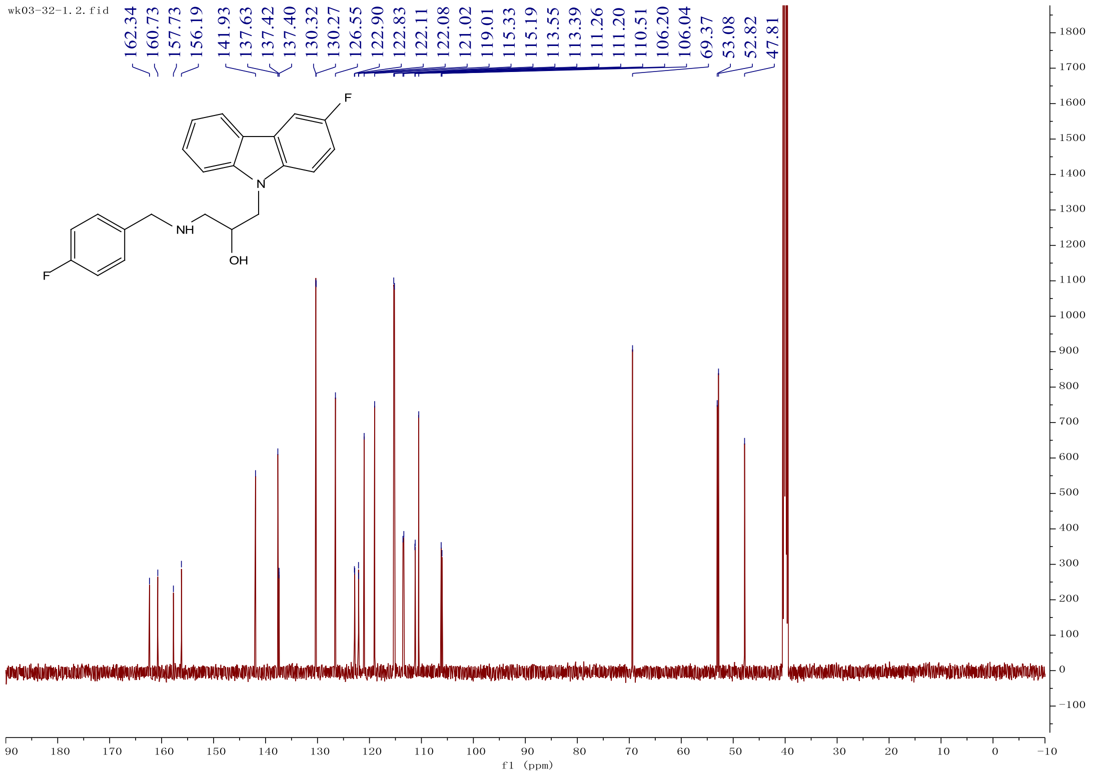

WK-9 ^{13}C NMR

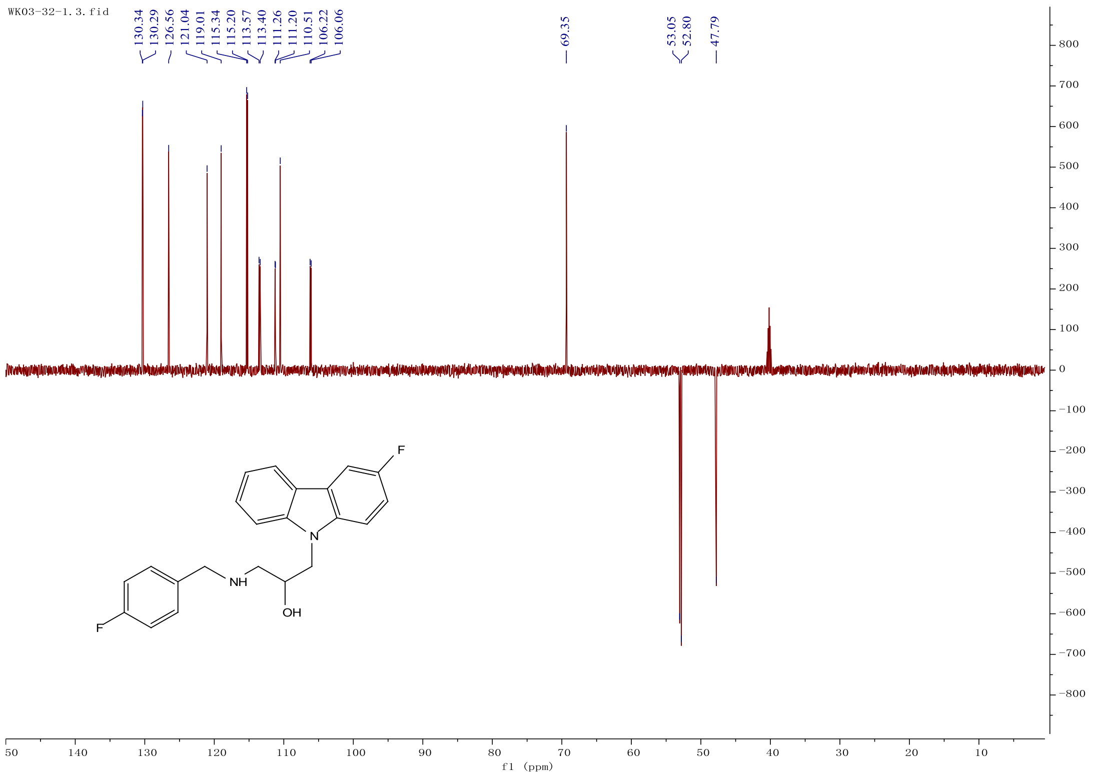

WK-9 Dept 135

(2S)-tert-butyl 4-(3-(3-fluoro-9H-carbazol-9-yl)-2-hydroxypropyl)-2-(hydroxymethyl)piperazine-1-carboxylate (**WK-10**).

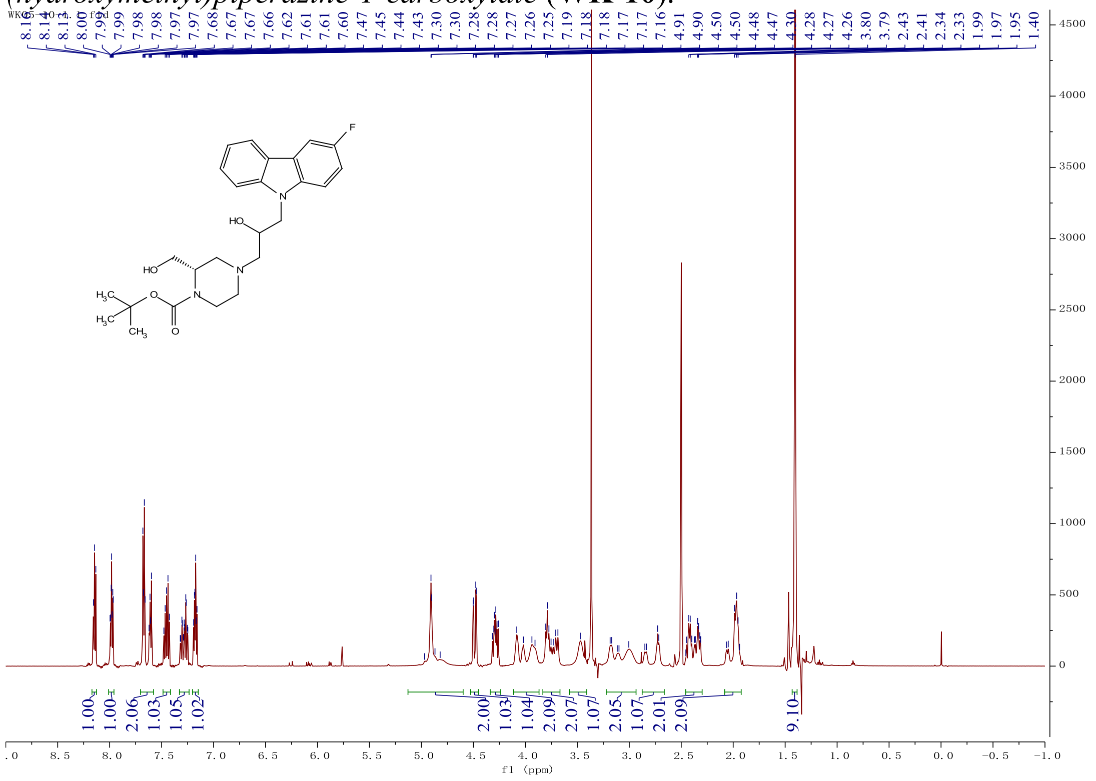

WK-10 ^{1}H NMR

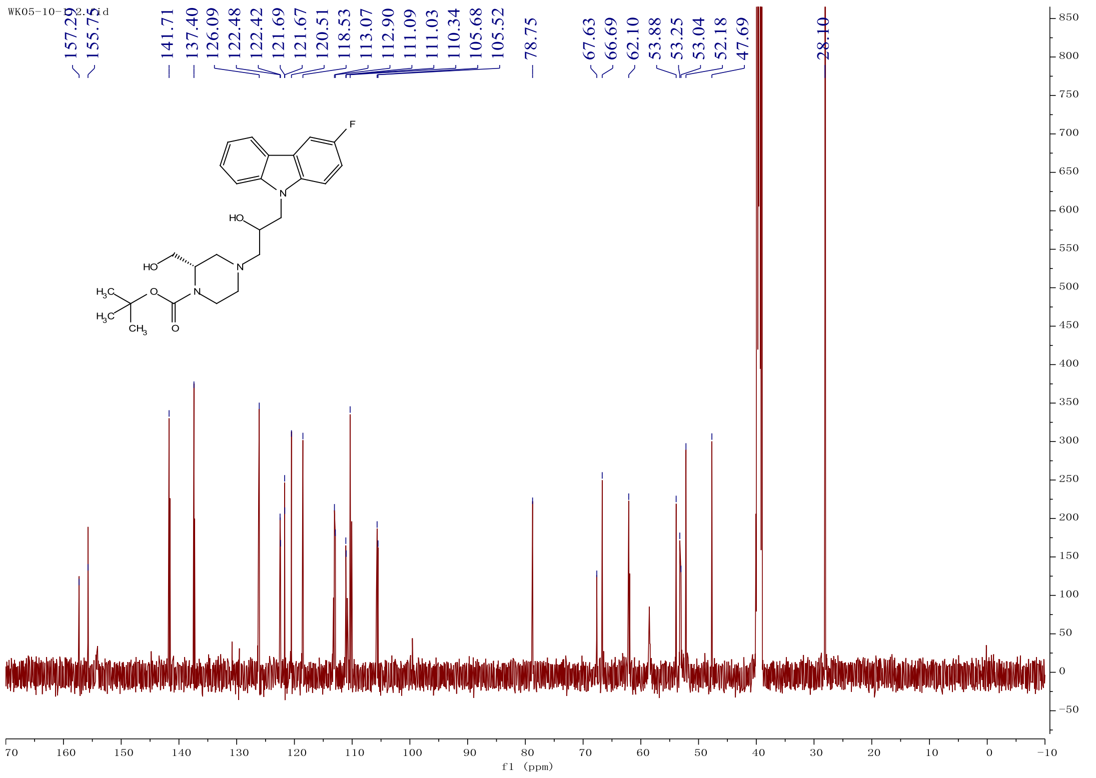

WK-10 ^{13}C NMR

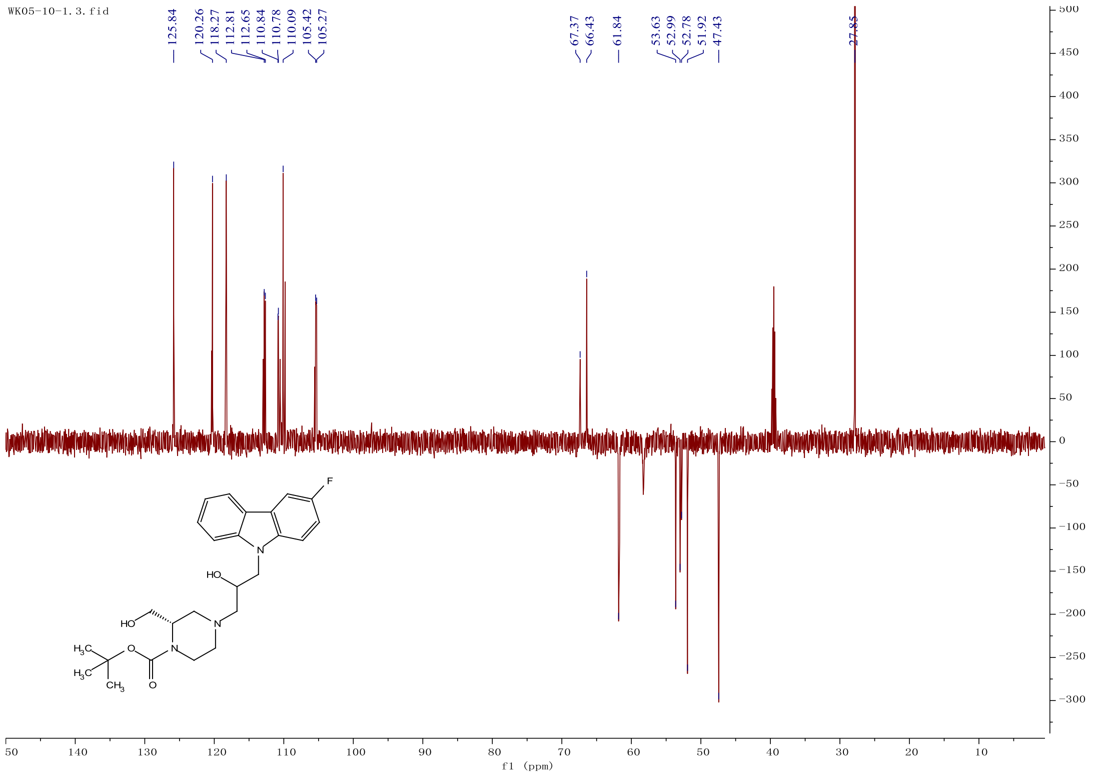

WK-10 Dept 135

1-(3-fluoro-9H-carbazol-9-yl)-3-((S)-3-(hydroxymethyl)piperazin-1-yl)propan-2-ol (WK-11).

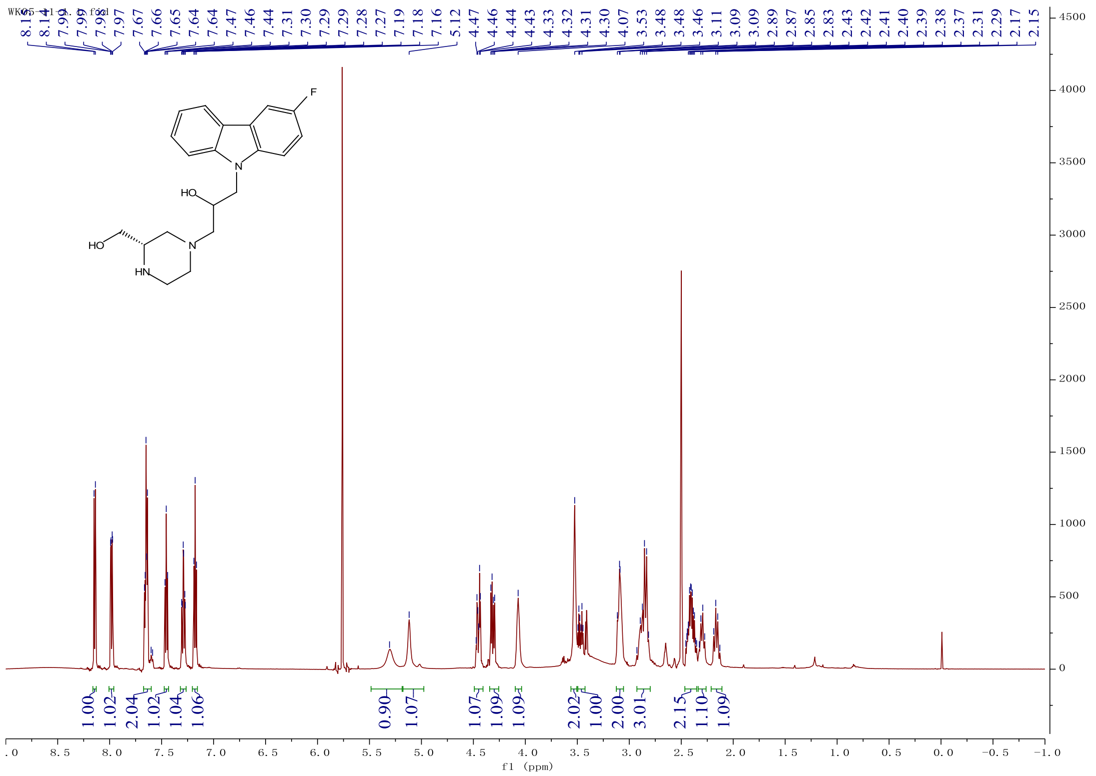

WK-11 ^{1}H NMR

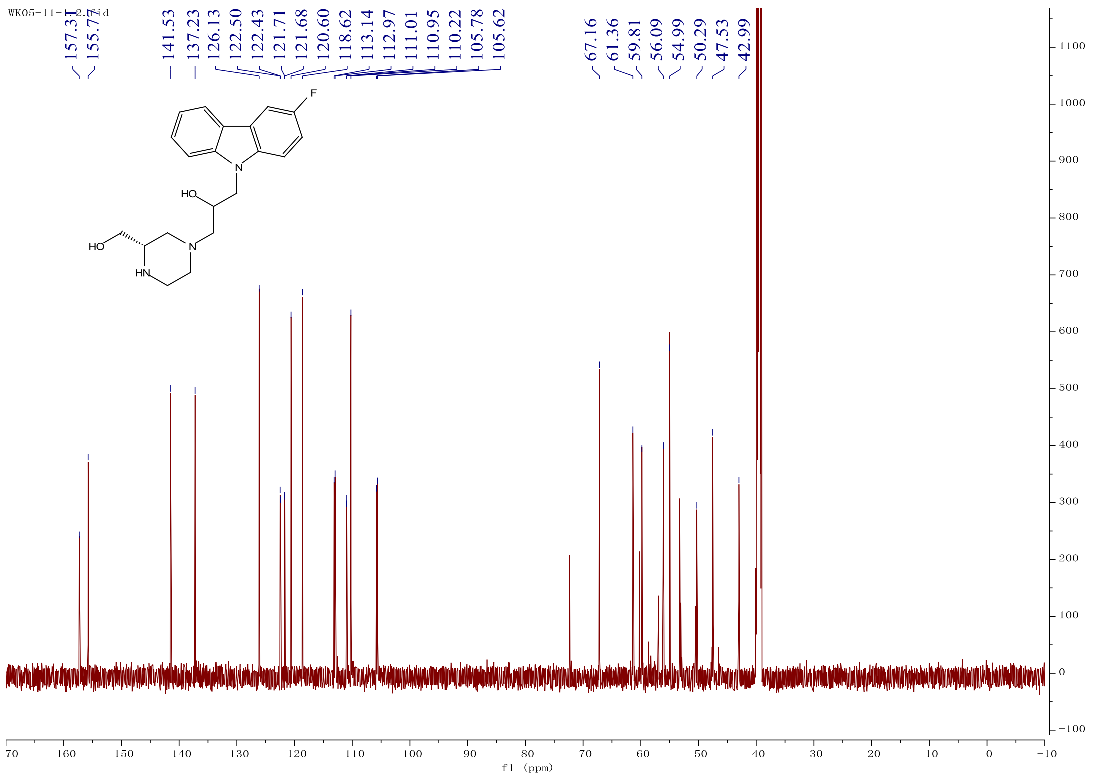

WK-11 ^{13}C NMR

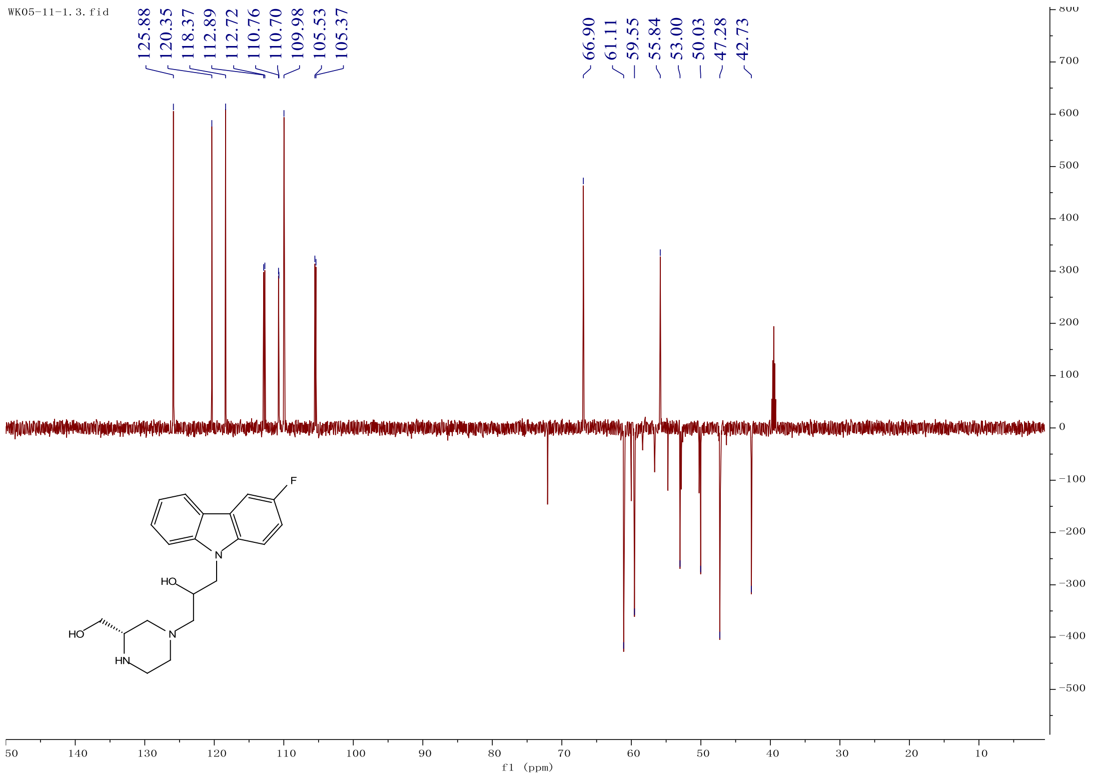

WK-11 Dept 135

***1-((2-(1H-indol-3-yl)ethyl)amino)-3-(3,6-difluoro-9H-carbazol-9-yl)propan-2-ol* (WK-12).**

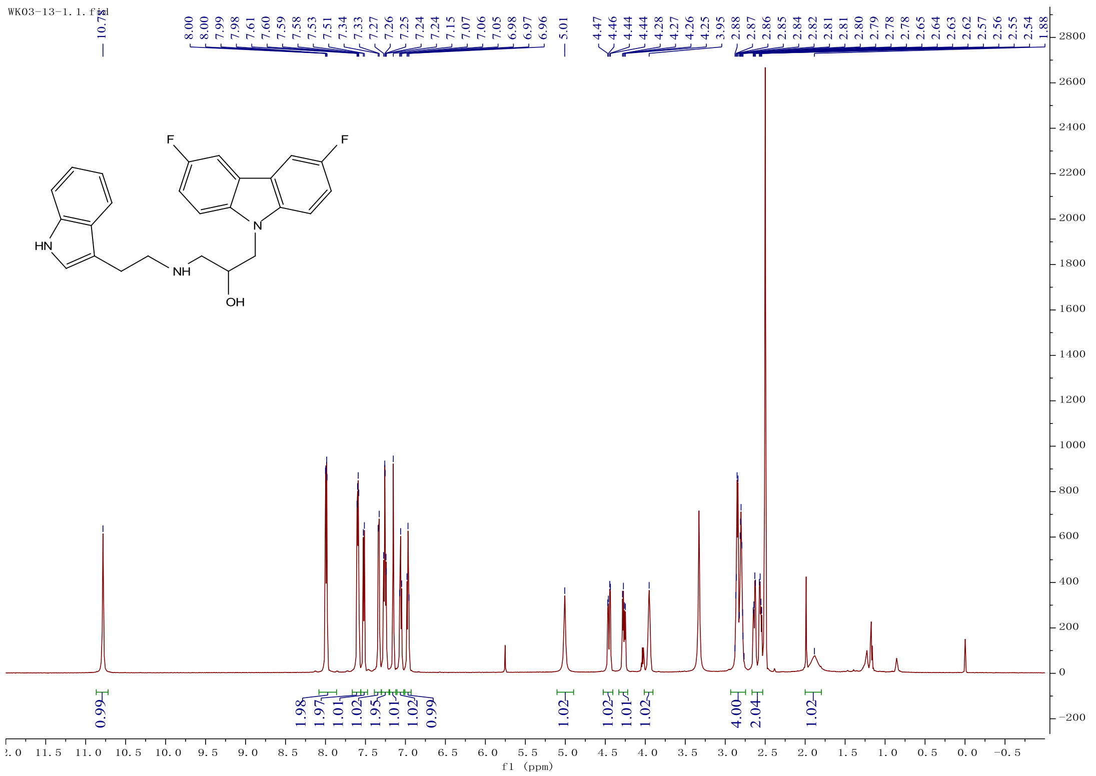

WK-12 ^{1}H NMR

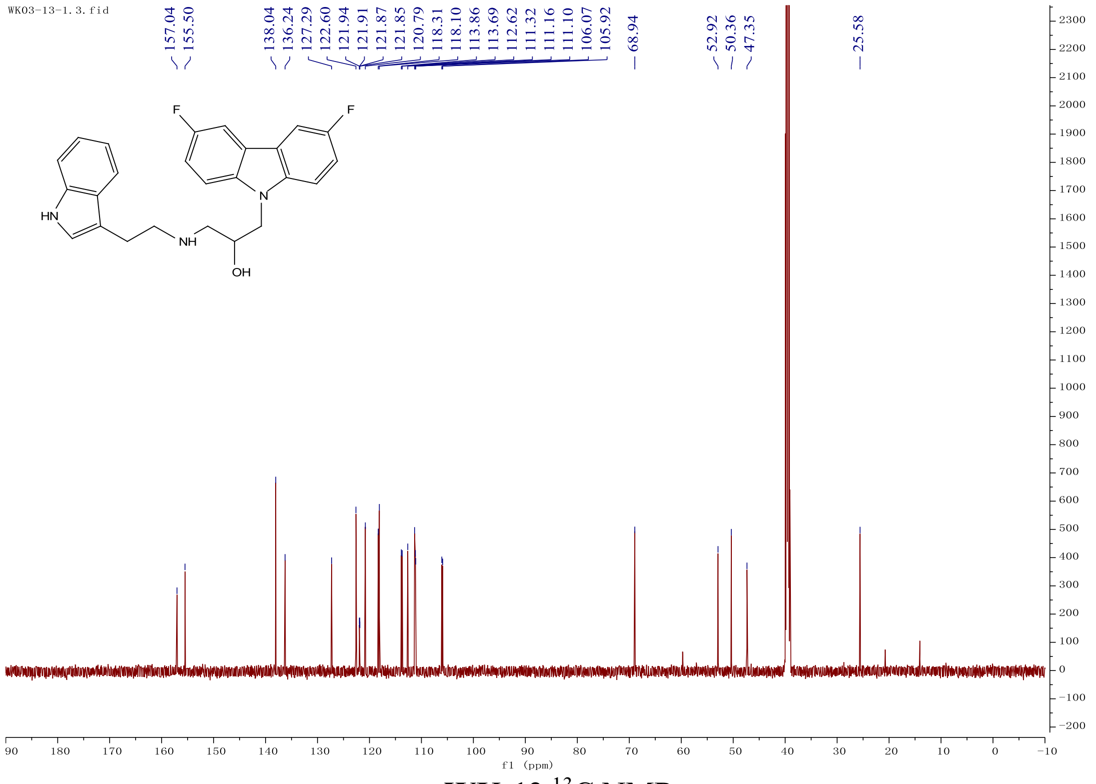

WK-12 ^{13}C NMR

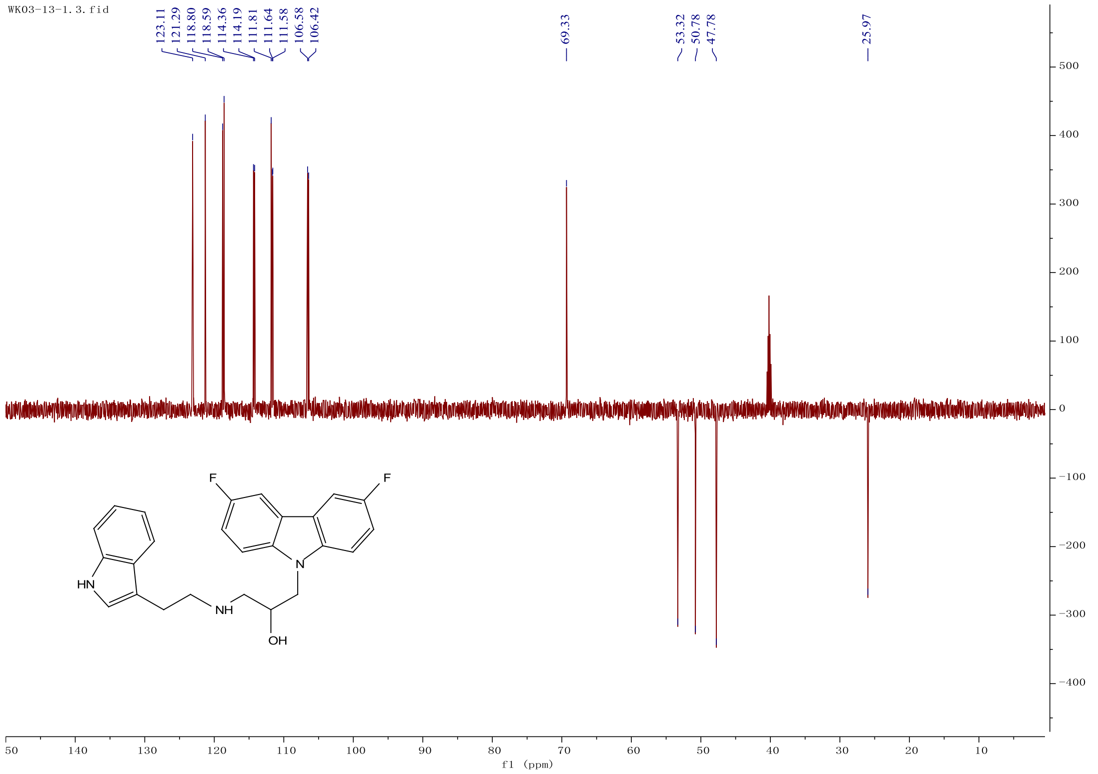

WK-12 Dept 135

***4-(2-((3-(3, 6-difluoro-9H-carbazol-9-yl)-2-hydroxypropyl)amino)ethyl)phenol* (WK-13).**

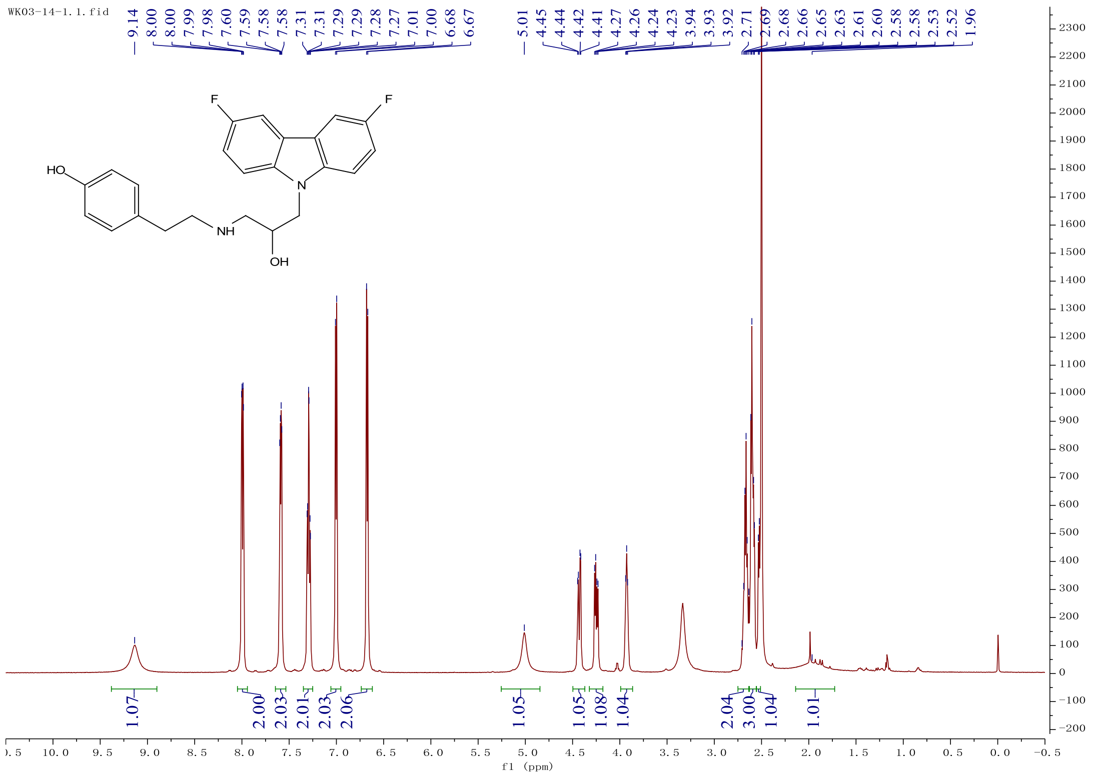

WK-13 ^{1}H NMR

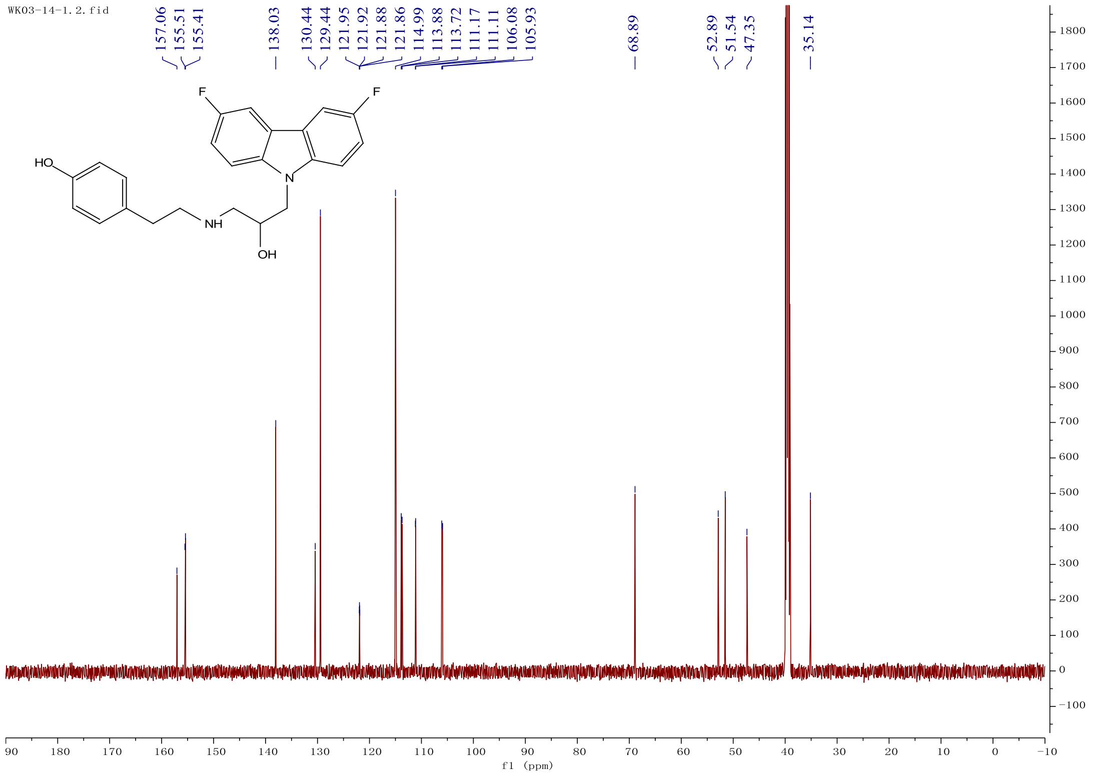

WK-13 ^{13}C NMR

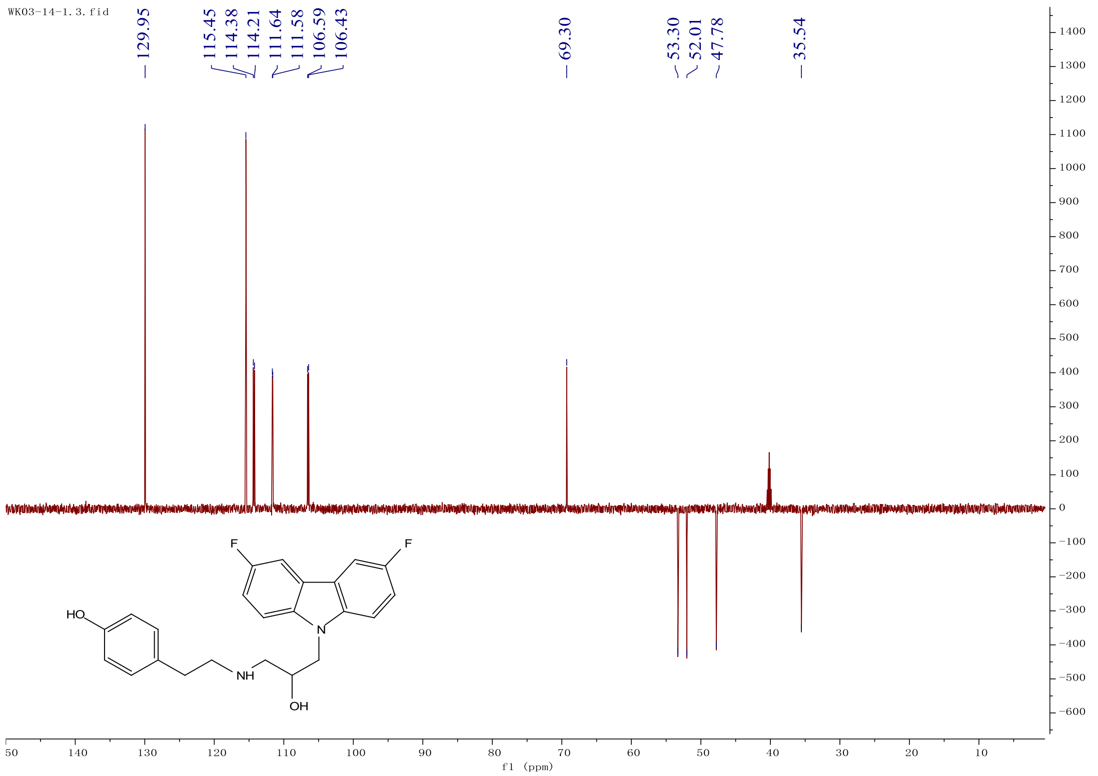

WK-13 Dept 135

***1-(3, 6-difluoro-9H-carbazol-9-yl)-3-((4-fluorophenethyl)amino)propan-2-ol* (WK-14).**

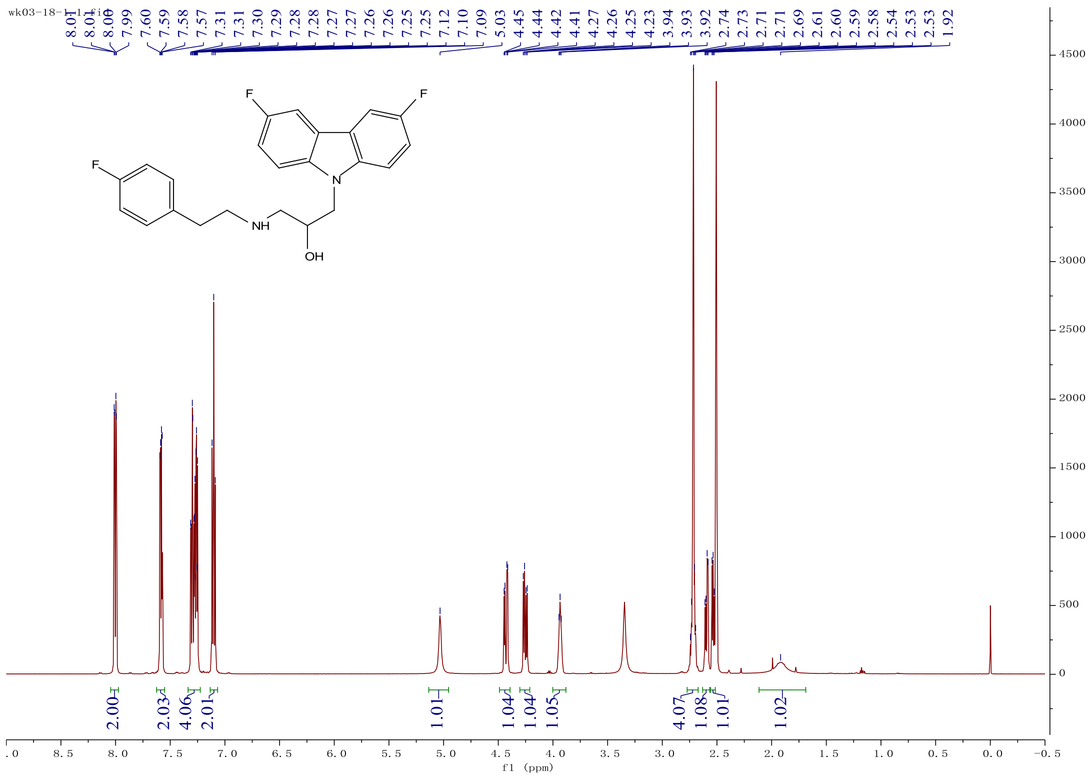

WK-14 ^{1}H NMR

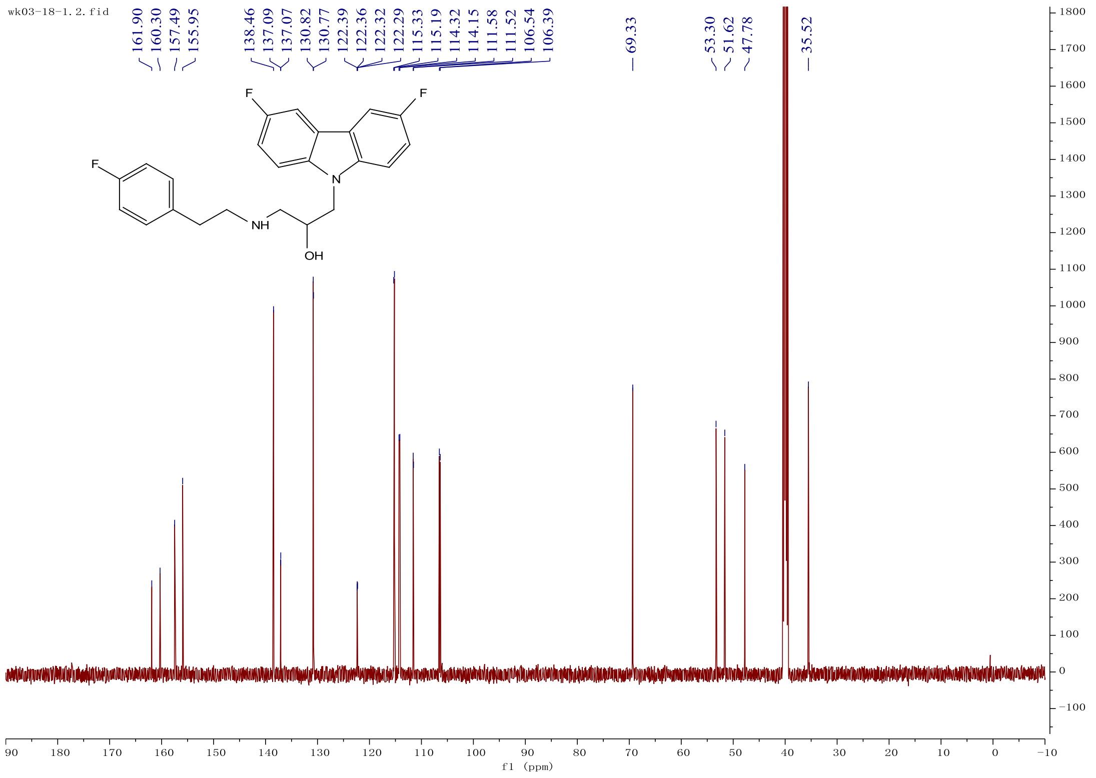

WK-14 ^{13}C NMR

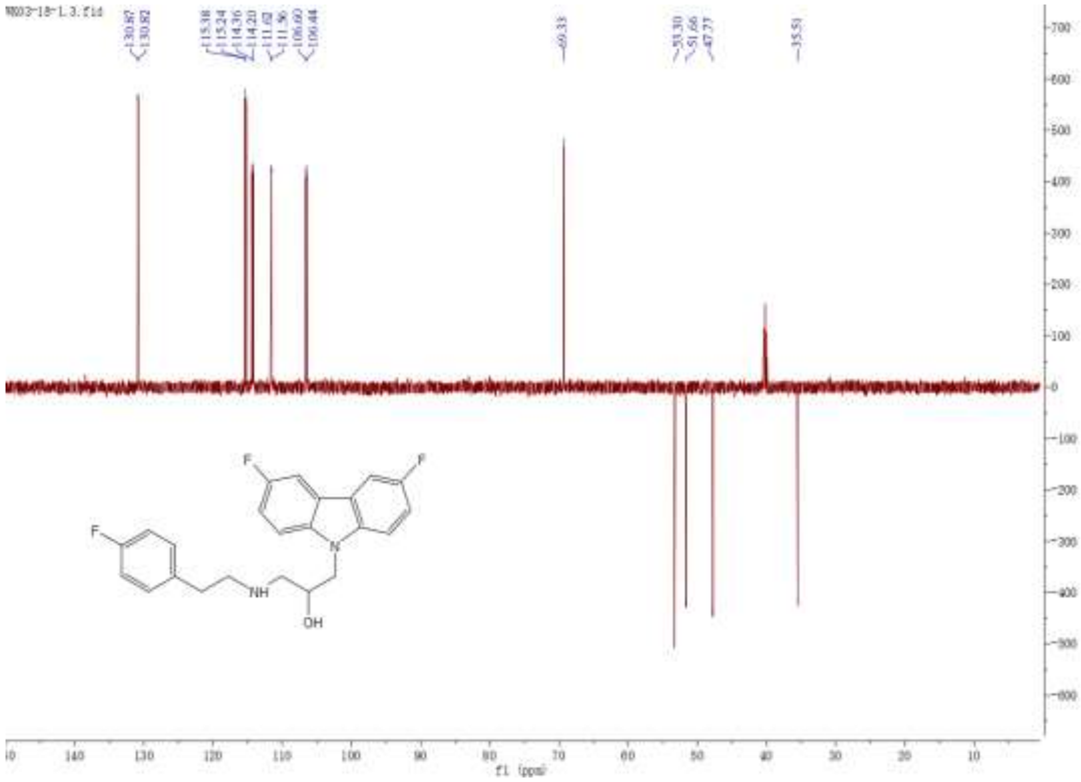

WK-14 Dept 135

***1-(3,6-difluoro-9H-carbazol-9-yl)-3-(phenethylamino)propan-2-ol* (WK-15).**

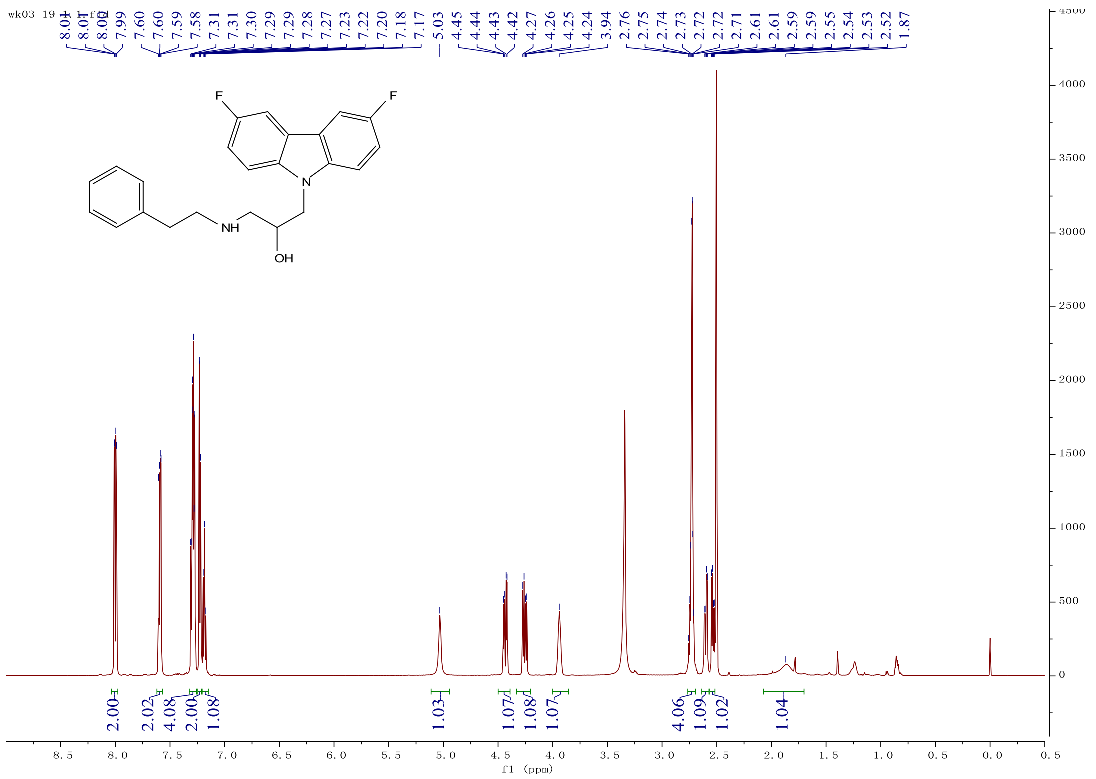

WK-15 ^{1}H NMR

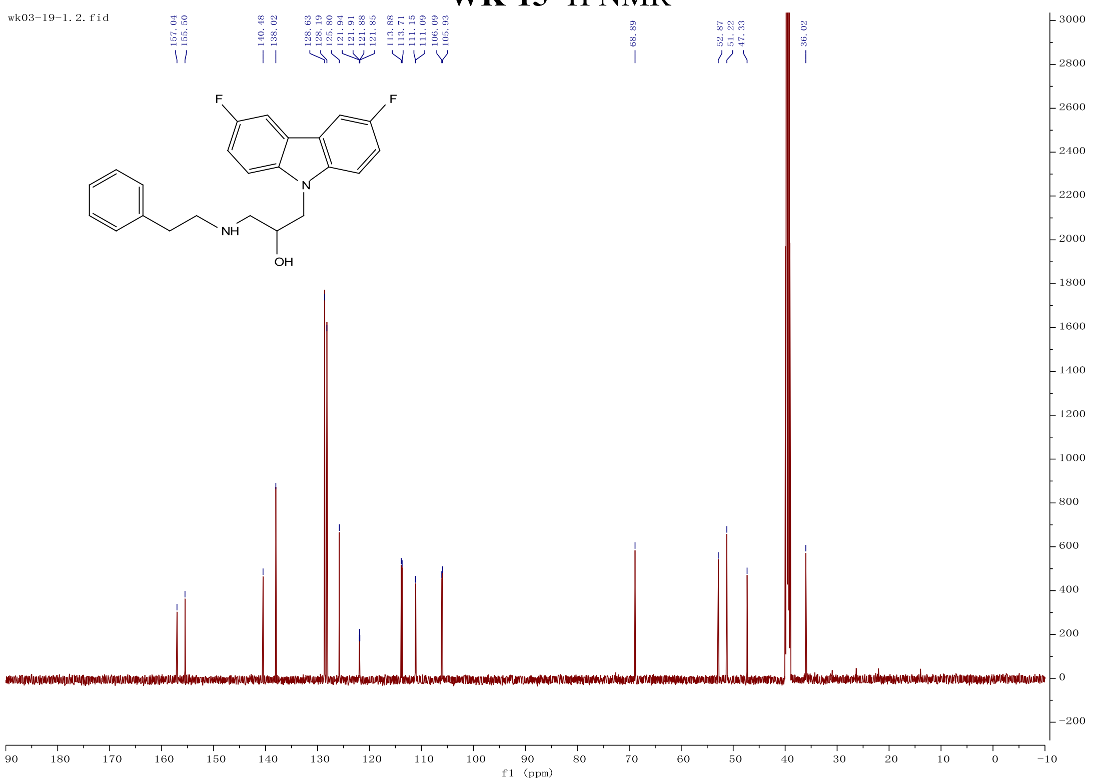

WK-15 ^{13}C NMR

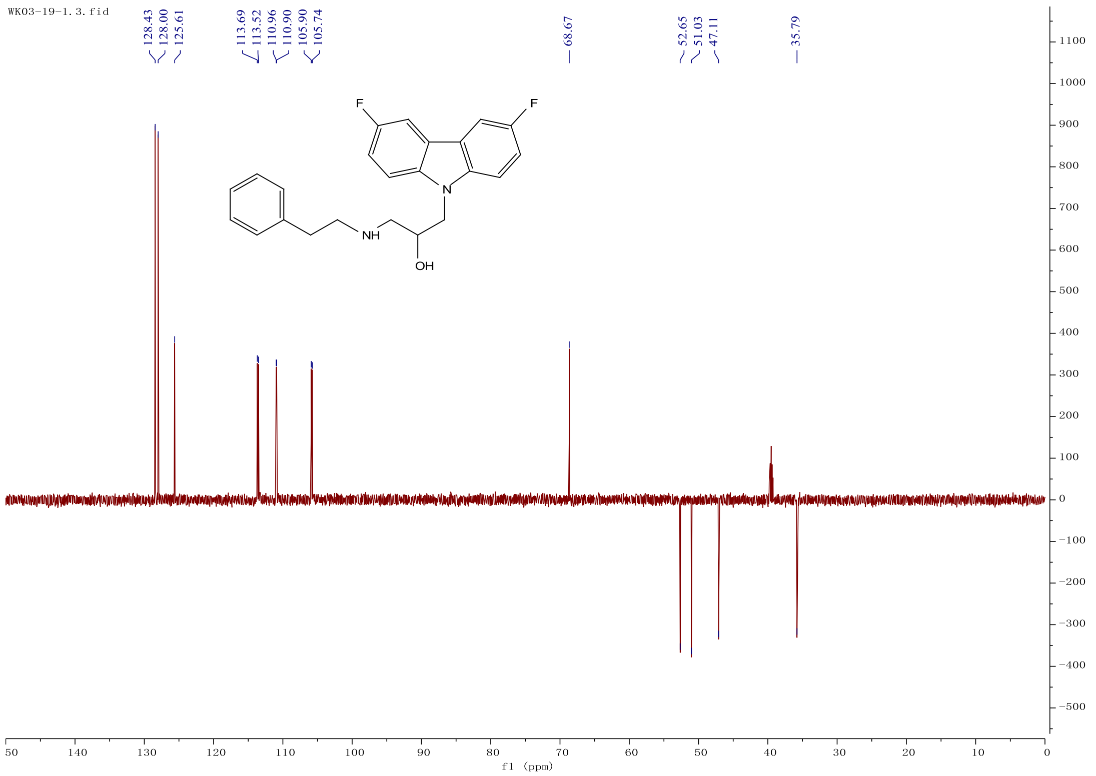

WK-15 Dept 135

***1-(3,6-difluoro-9H-carbazol-9-yl)-3-((2-(2-methoxyphenoxy)ethyl)amino)propan-2-ol* (WK-16).**

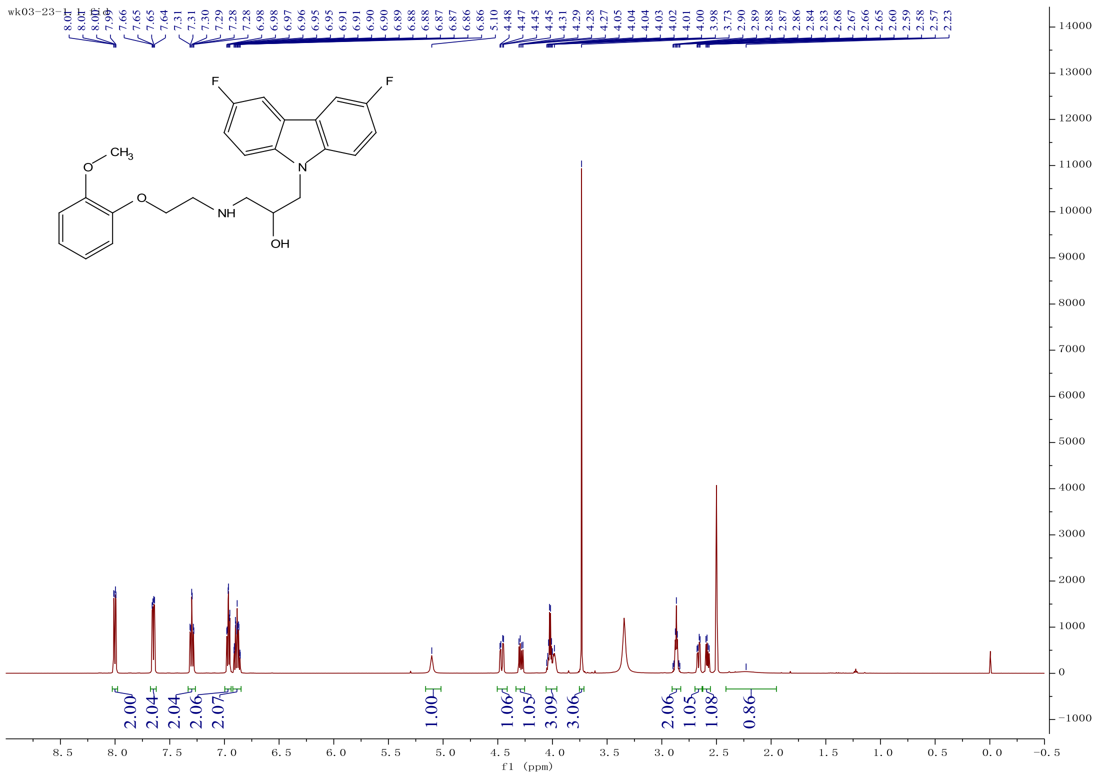

WK-16 ^{1}H NMR

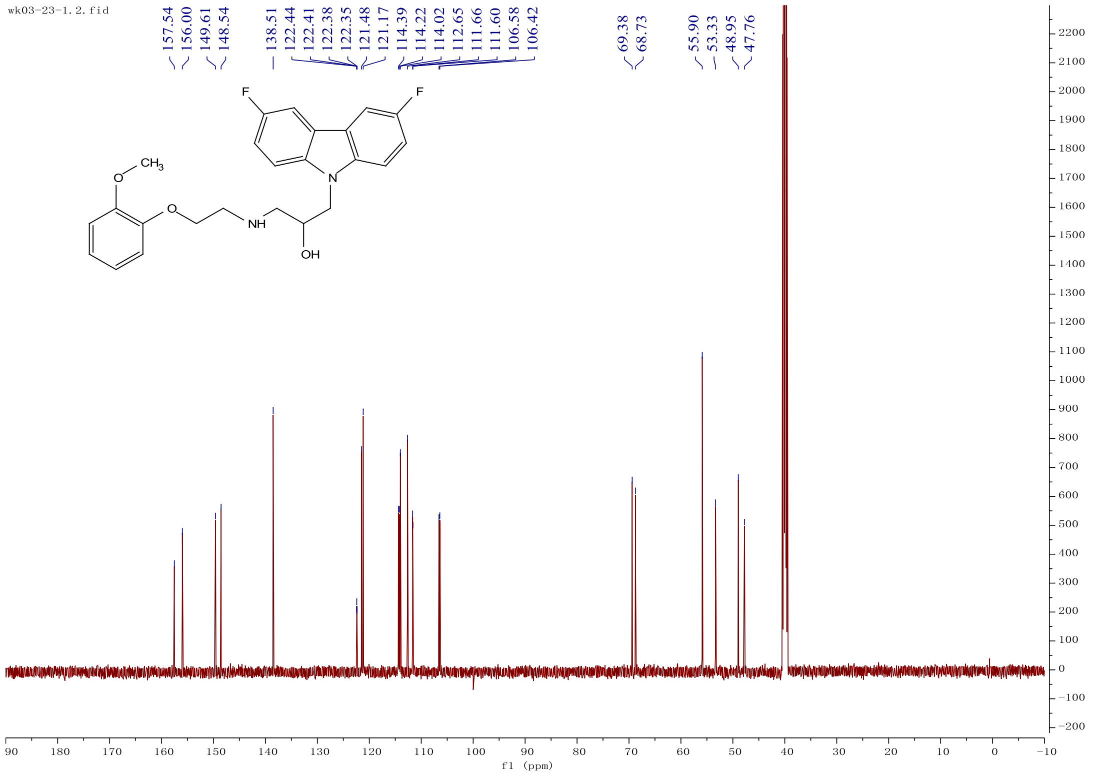

WK-16 ^{13}C NMR

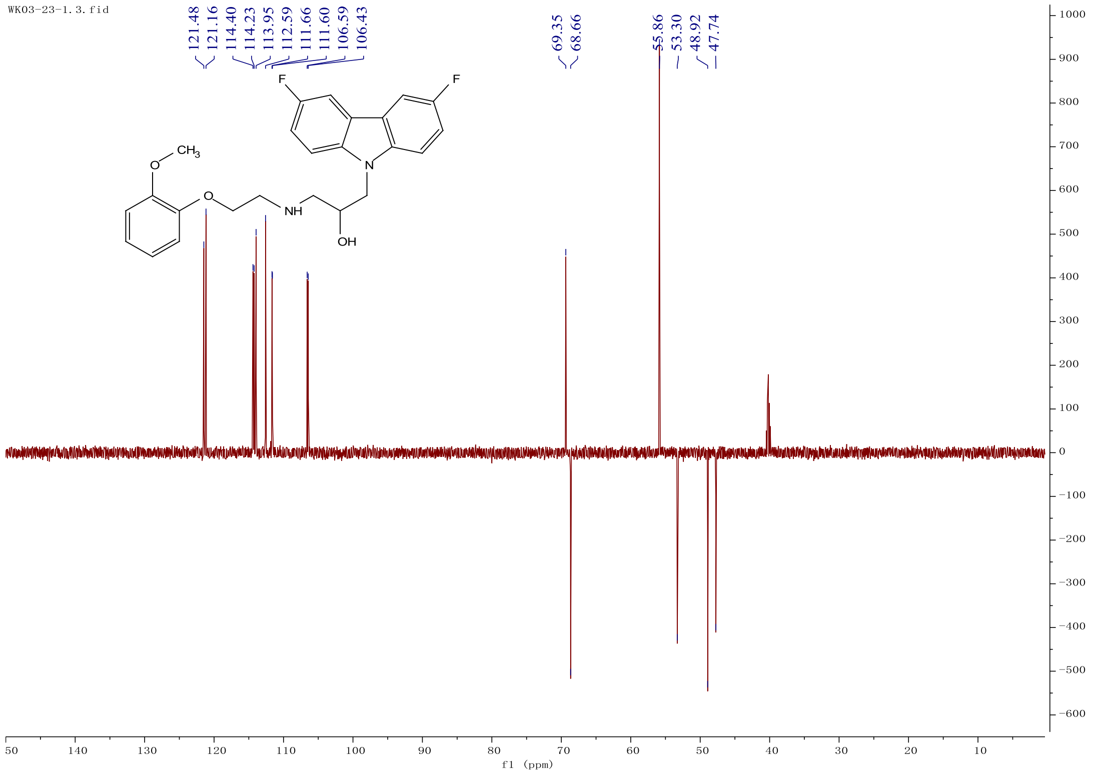

WK-16 Dept 135

***1-((3-butoxypropyl)amino)-3-(3,6-difluoro-9H-carbazol-9-yl)propan-2-ol* (WK-17).**

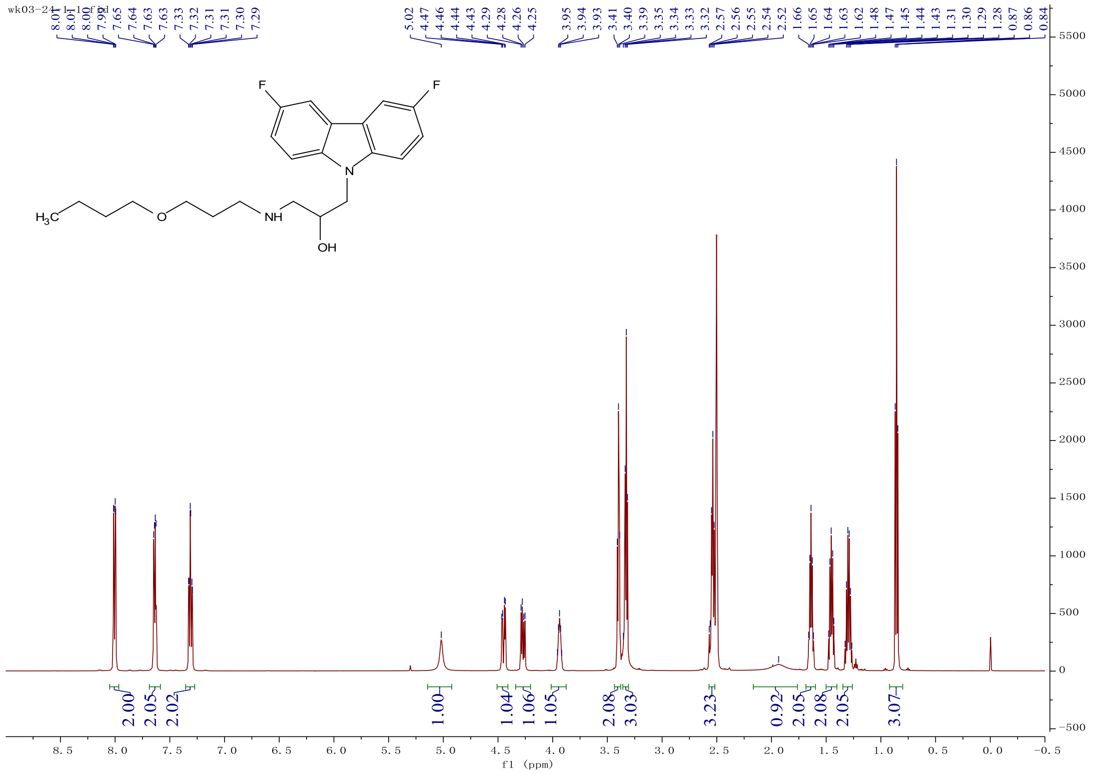

WK-17 ^{1}H NMR

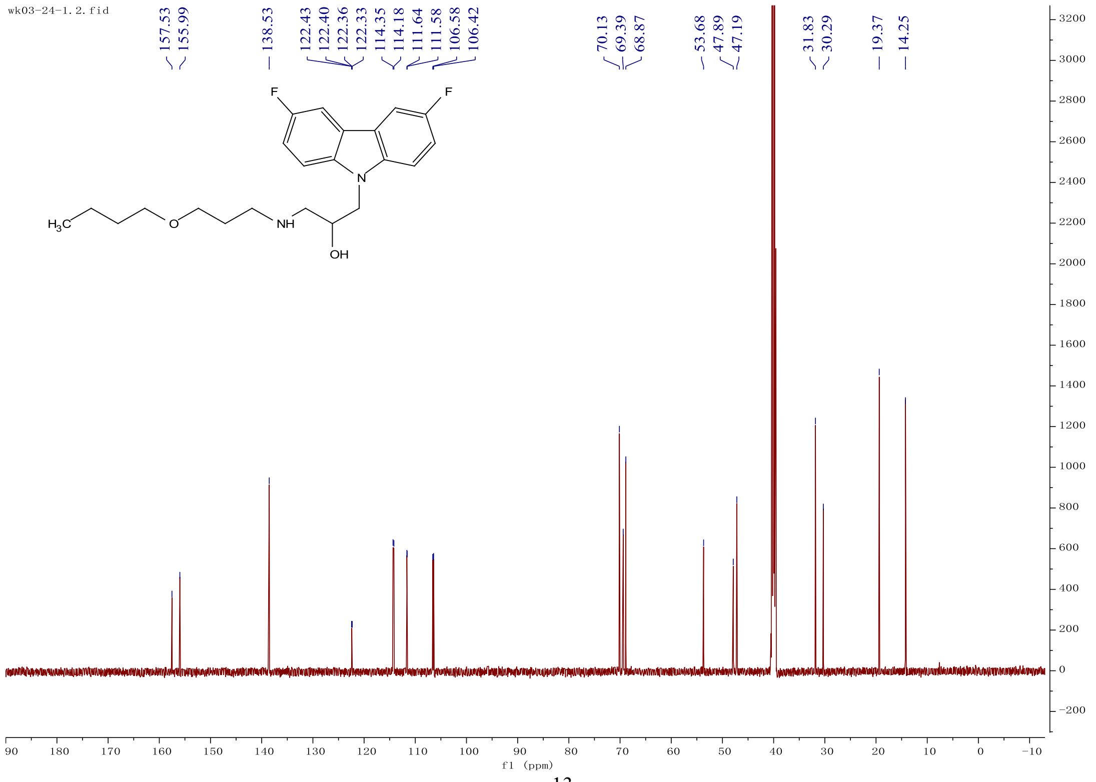

WK-17 ^{13}C NMR

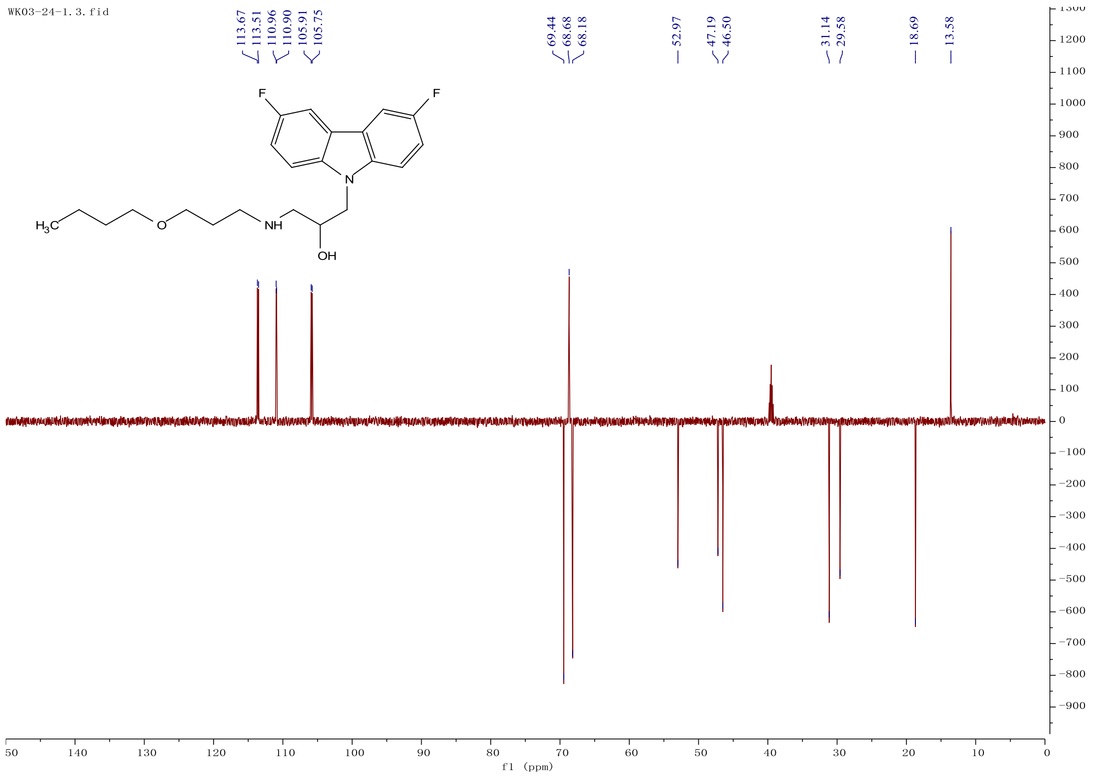

WK-17 Dept 135

***1-(3, 6-difluoro-9H-carbazol-9-yl)-3-((2-hydroxypropyl)amino)propan-2-ol* (WK-18).**

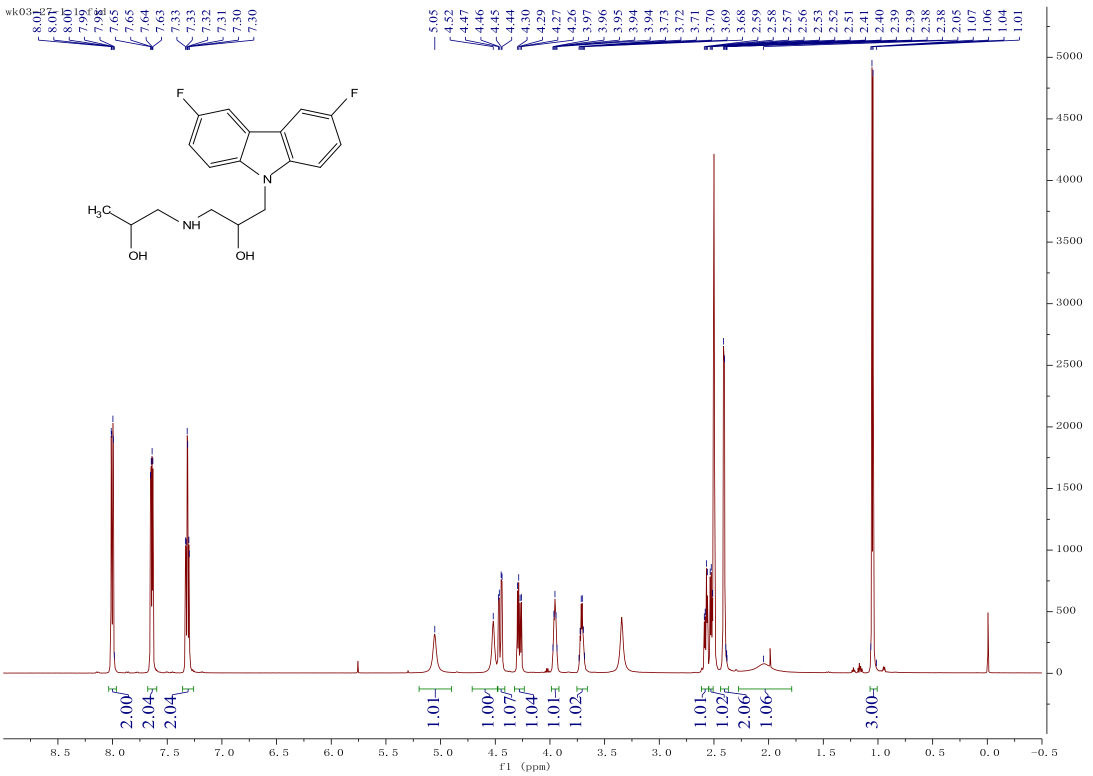

WK-18 ^{1}H NMR

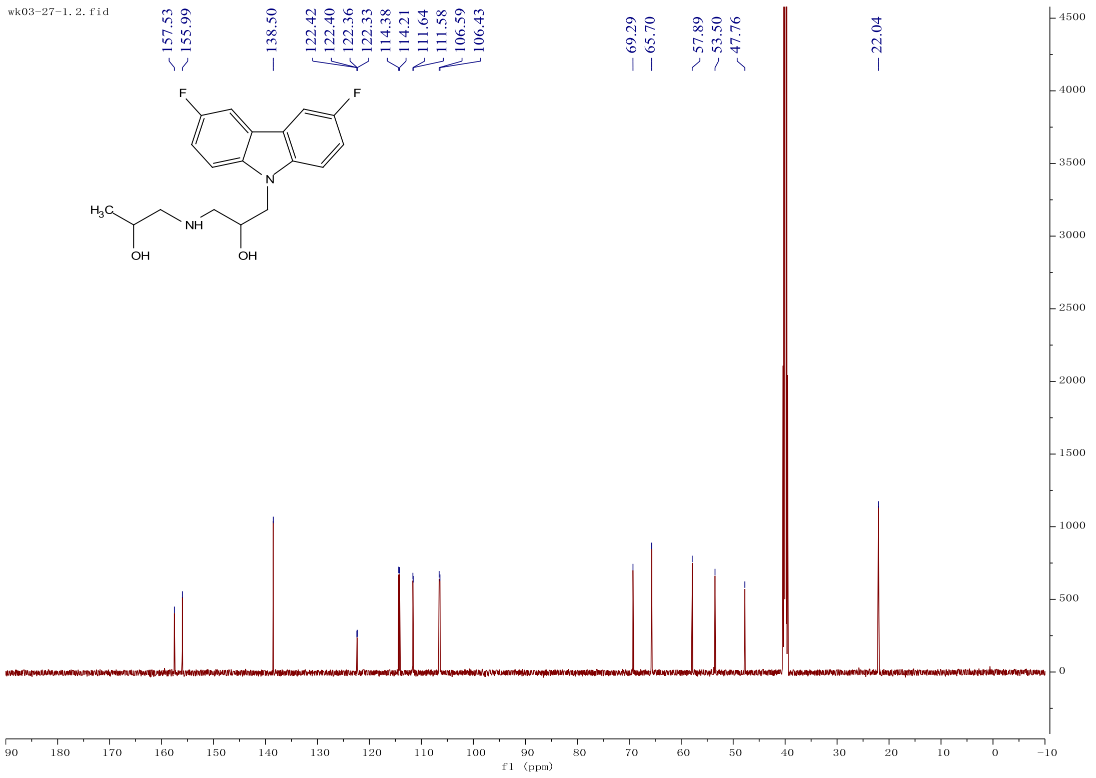

WK-18 ^{13}C NMR

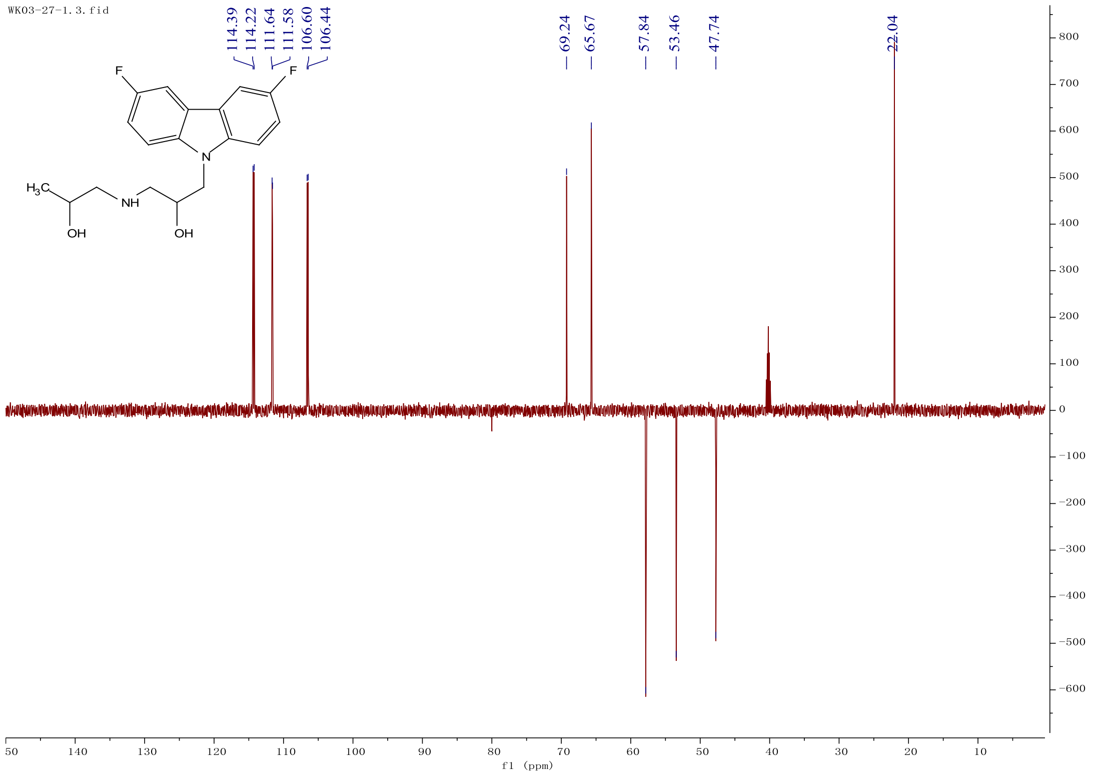

WK-18 Dept 135

2-((3-(3,6-difluoro-9H-carbazol-9-yl)-2-hydroxypropyl)amino)propan-1-ol **(WK-19).**

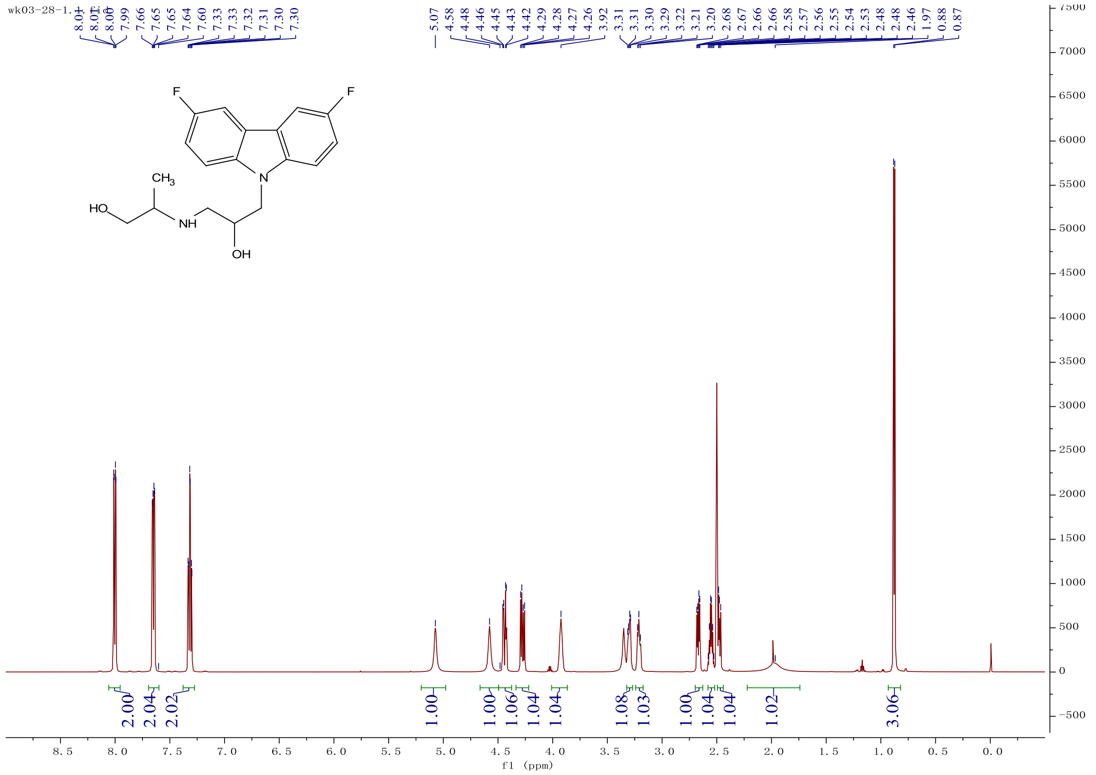

WK-19 ^{1}H NMR

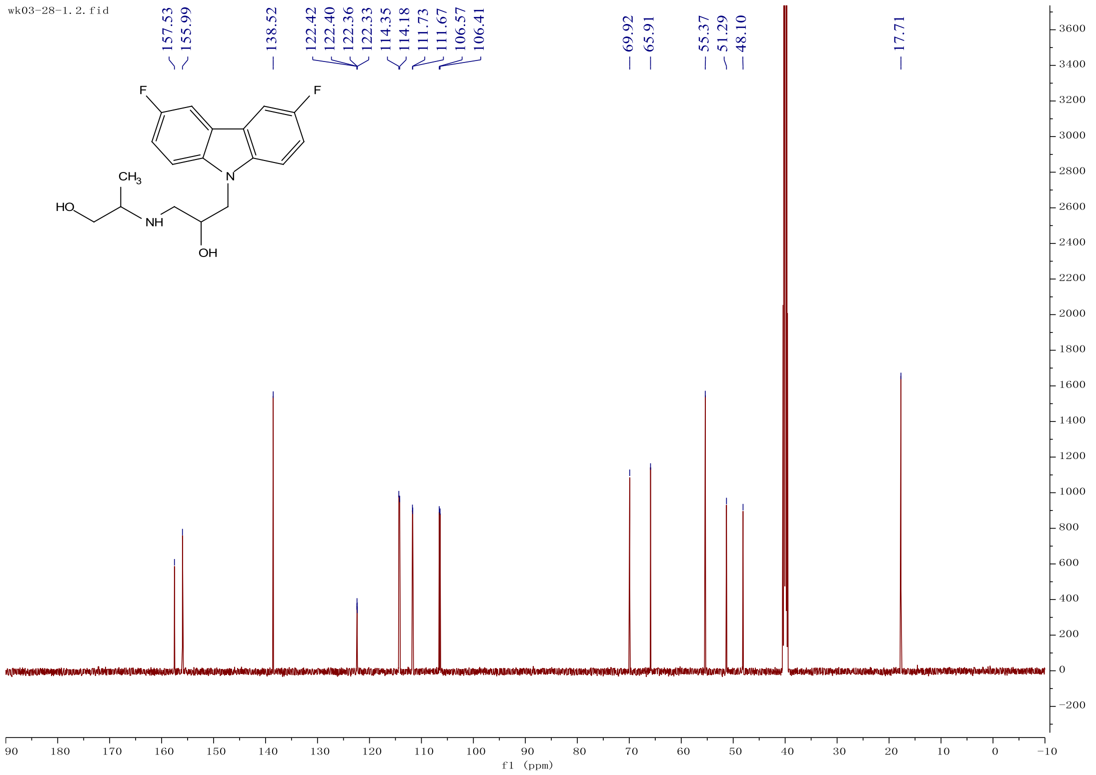

WK-19 ^{13}C NMR

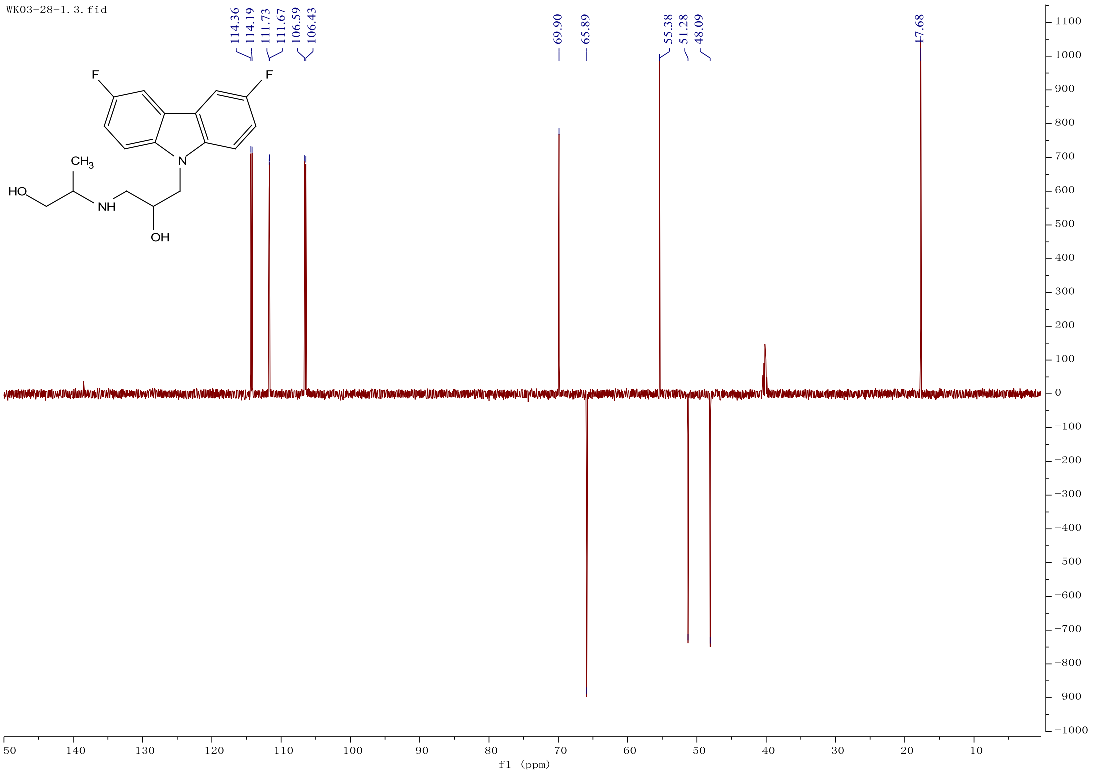

WK-19 Dept 135

***1-((2-(1H-indol-3-yl)ethyl)amino)-3-(9H-pyrido[3,4-b]indol-9-yl)propan-2-ol* (WK-20).**

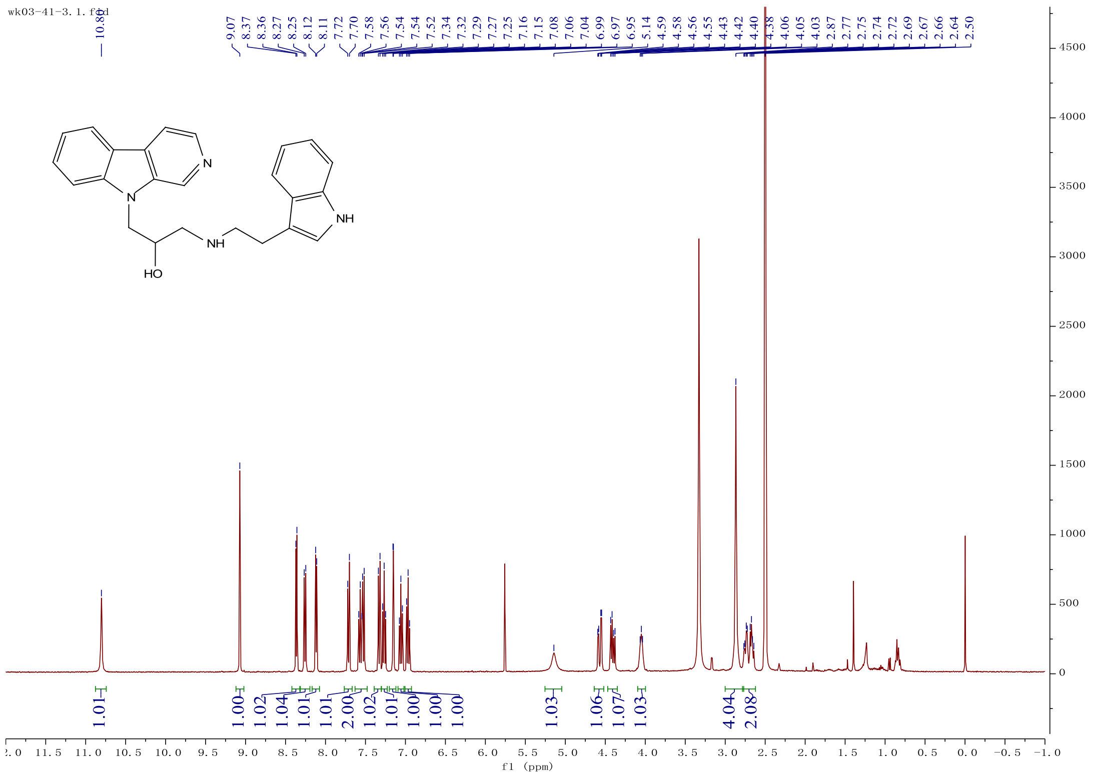

WK-20 ^{1}H NMR

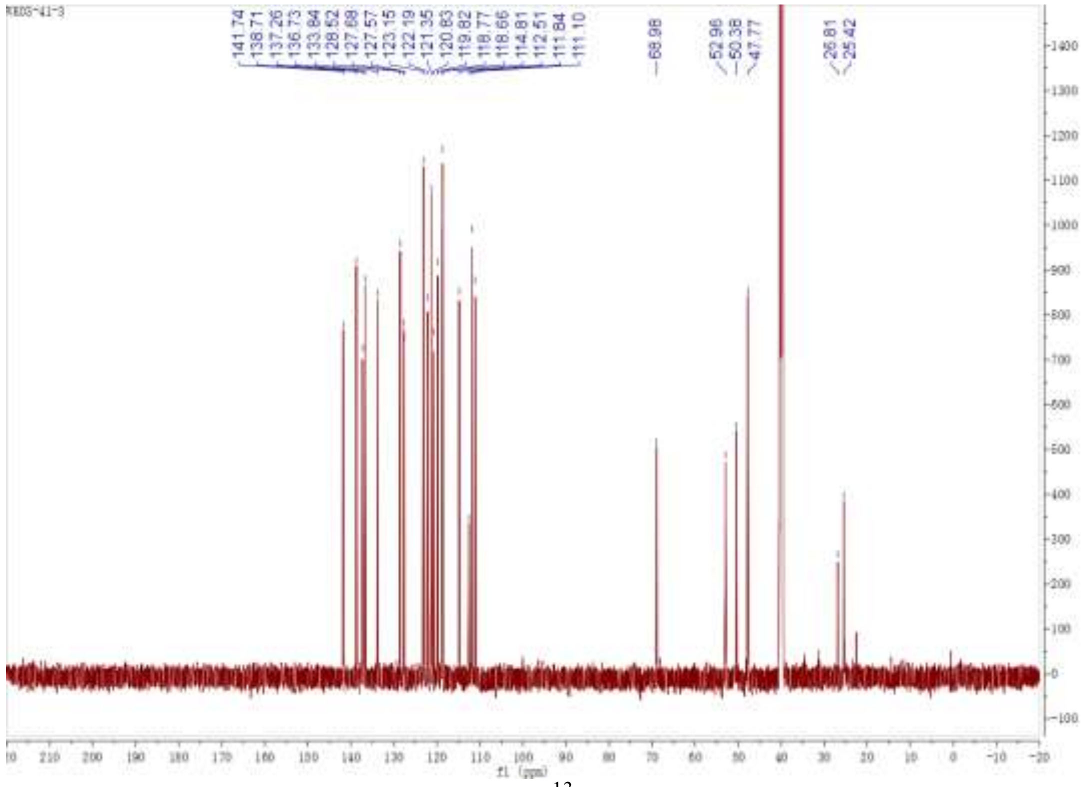

WK-20 ^{13}C NMR

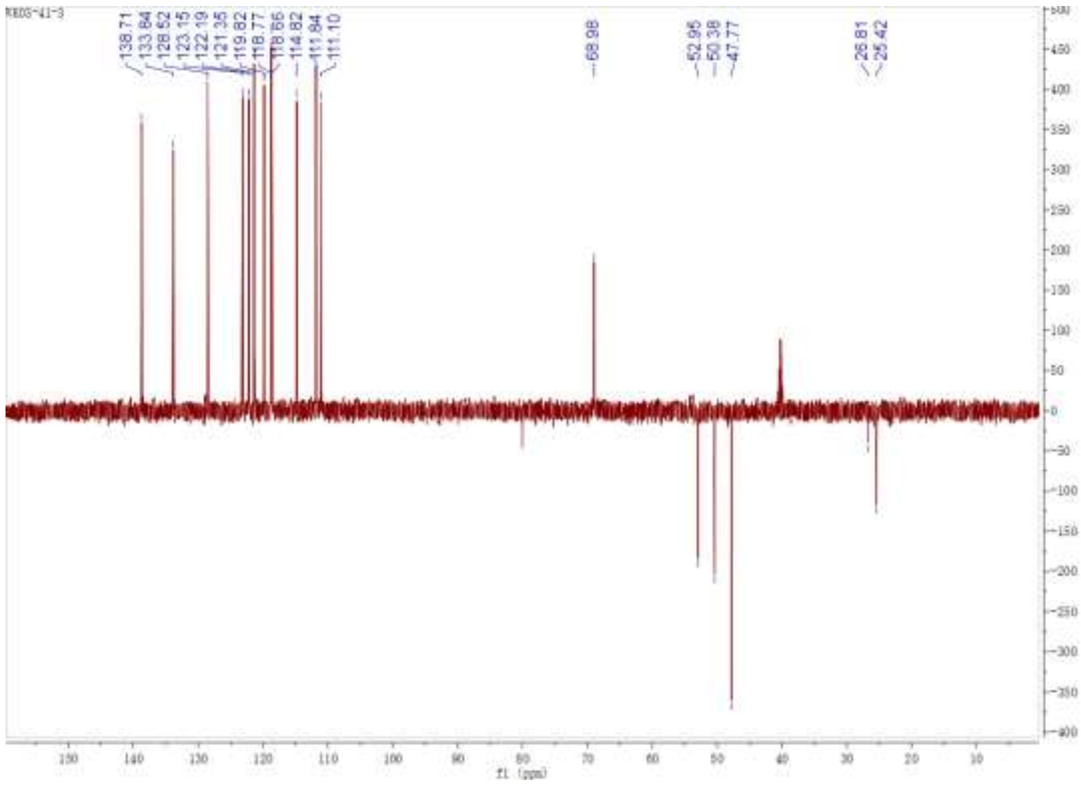

WK-20 Dept 135

***1-((2-(1H-indol-3-yl)ethyl)amino)-3-(9H-pyrido[2,3-b]indol-9-yl)propan-2-ol* (WK-21)**

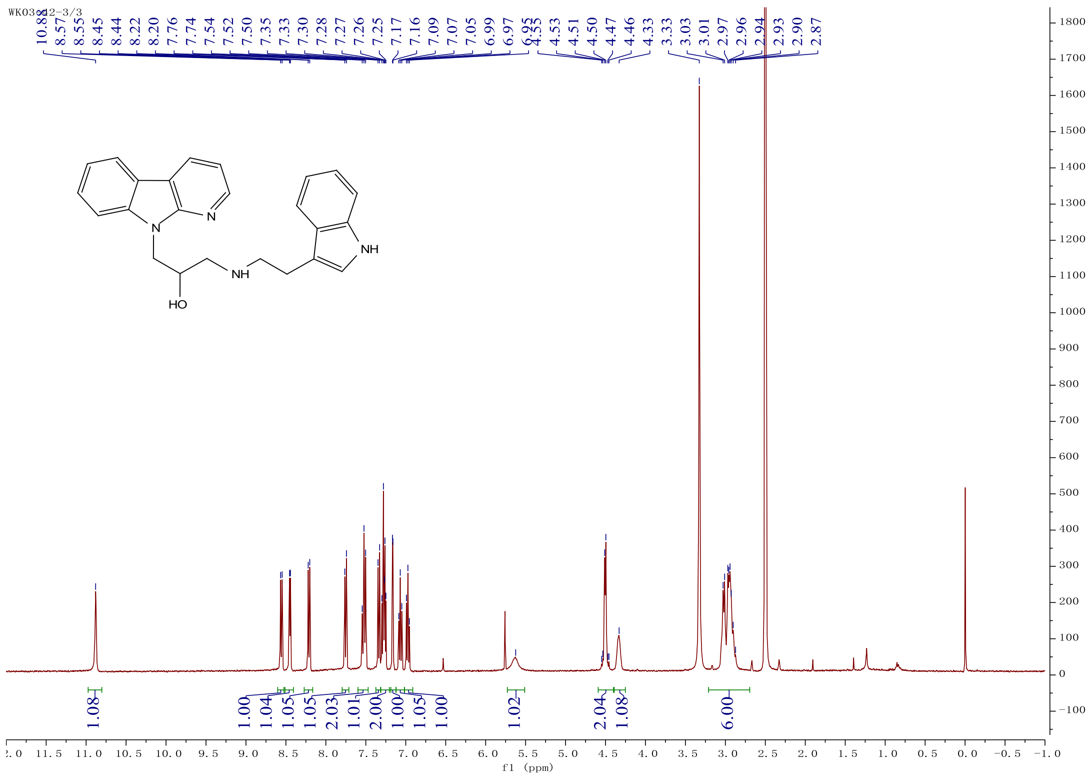

WK-21 ^{1}H NMR

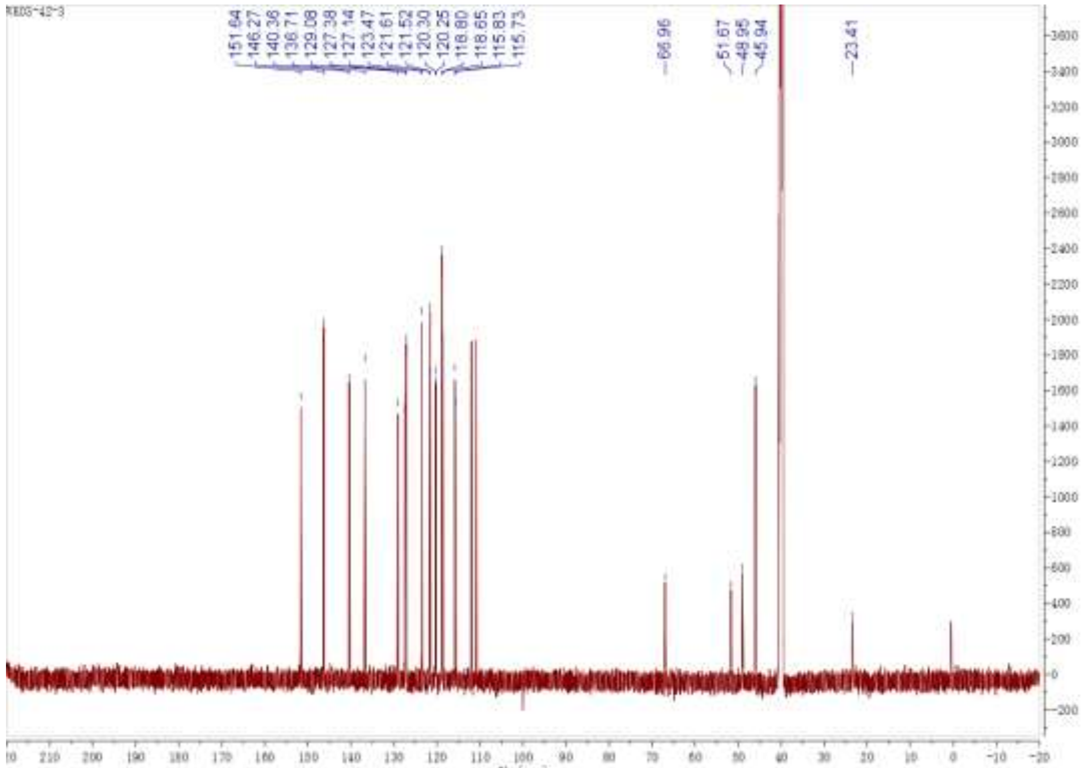

WK-21 ^{13}C NMR

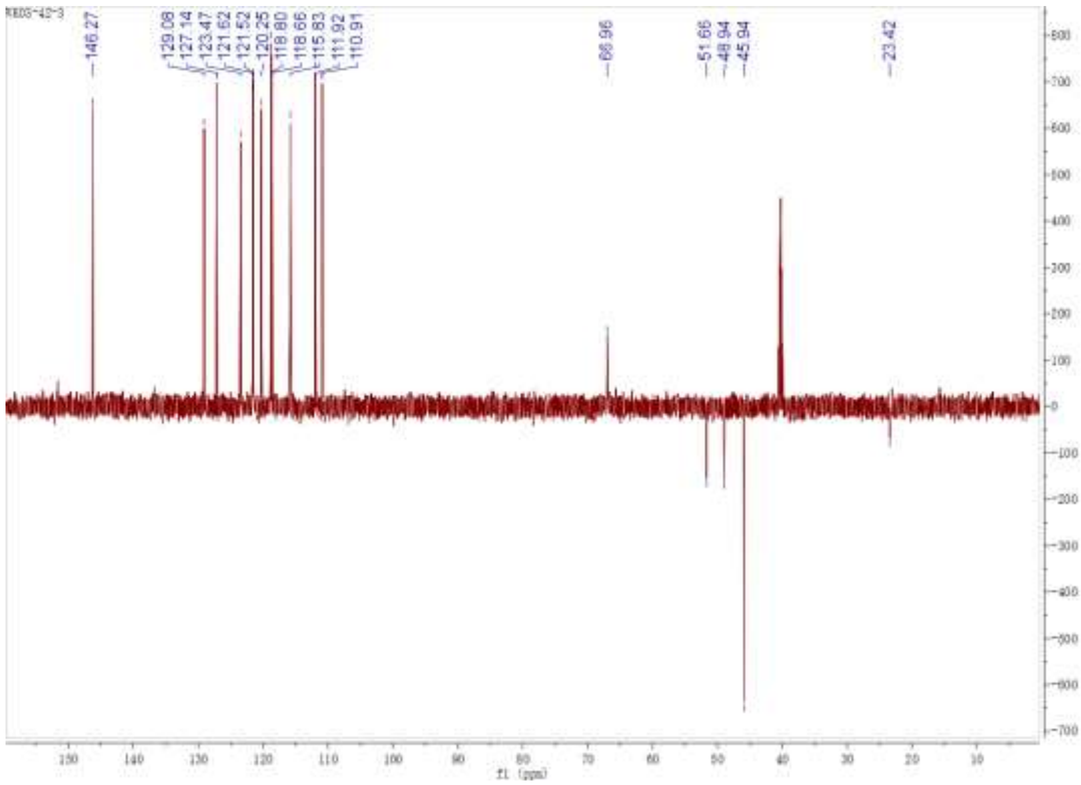

WK-21 Dept 135

***1-((1,3-bis(3,6-difluoro-9H-carbazol-9-yl)propan-2-yl)oxy)-3-(isopropylamino)propan-2-ol* (WK-22).**

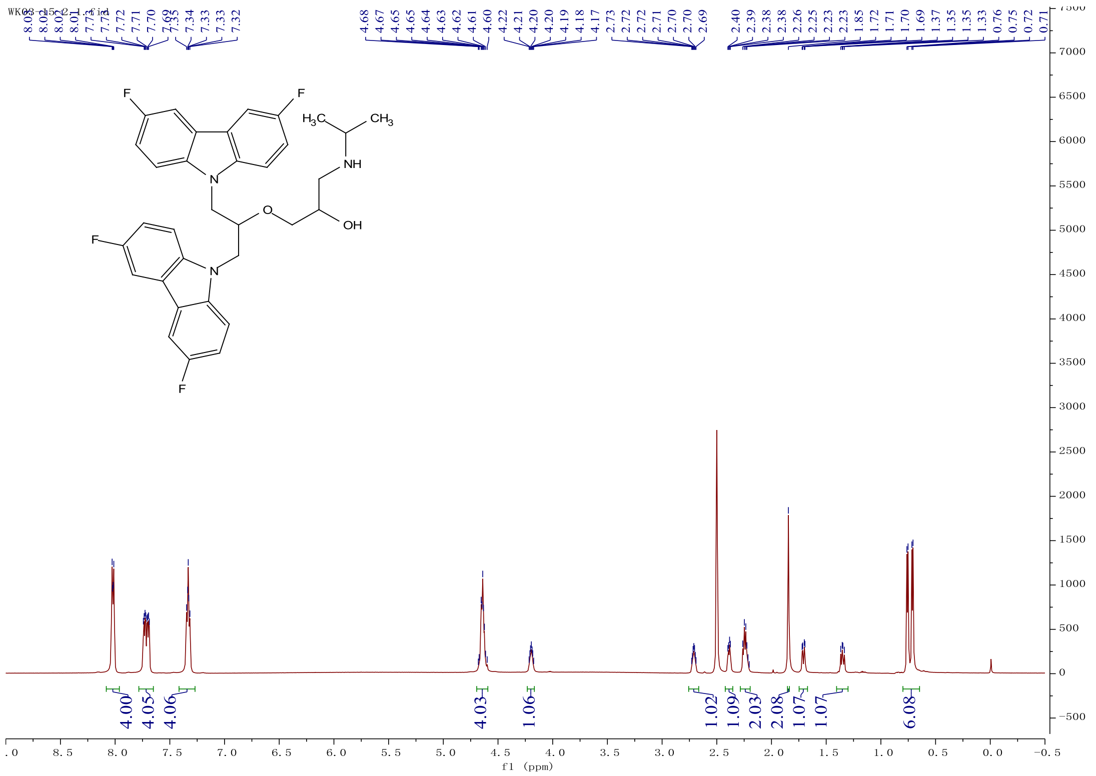

WK-22 ^{1}H NMR

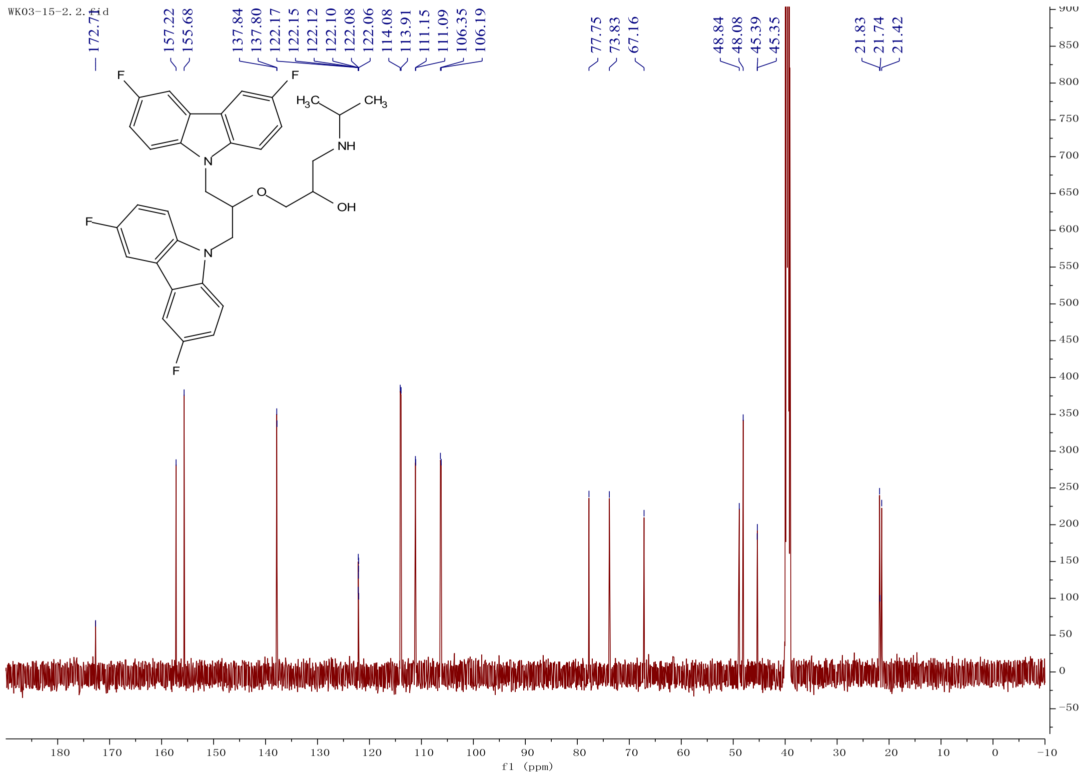

WK-22 ^{13}C NMR

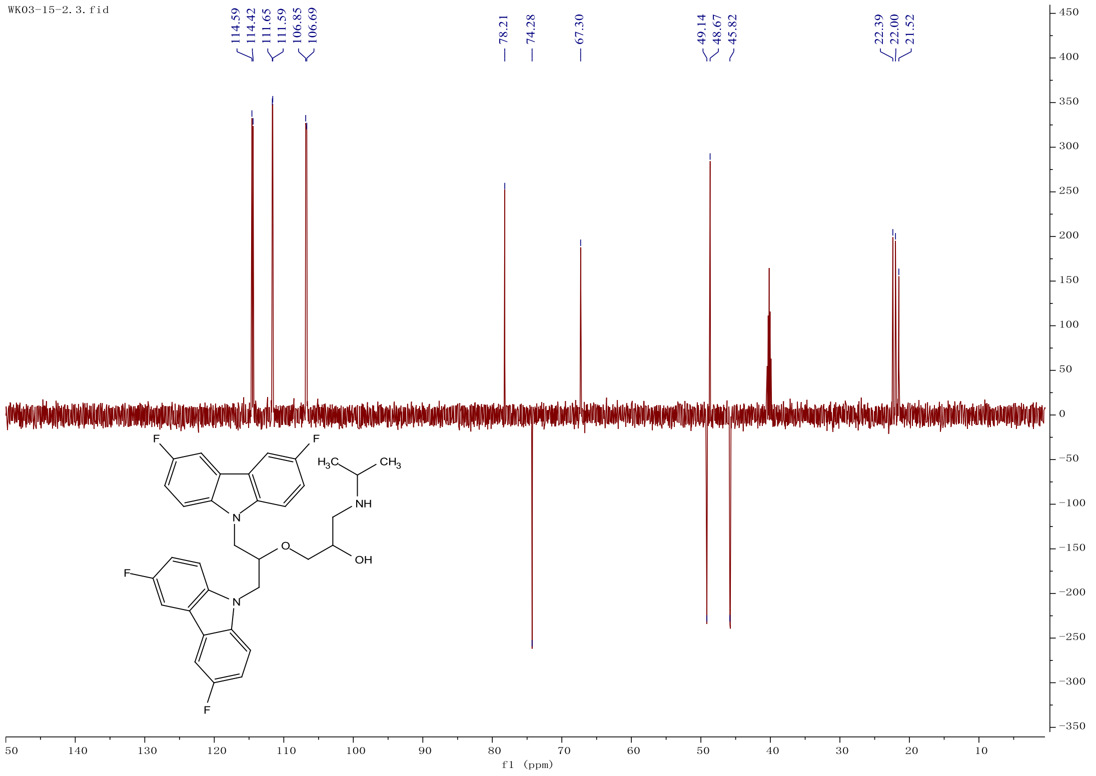

WK-22 Dept 135

***1-((1,3-bis(3-fluoro-9H-carbazol-9-yl)propan-2-yl)oxy)-3-(isopropylamino)propan-2-ol* (WK-23).**

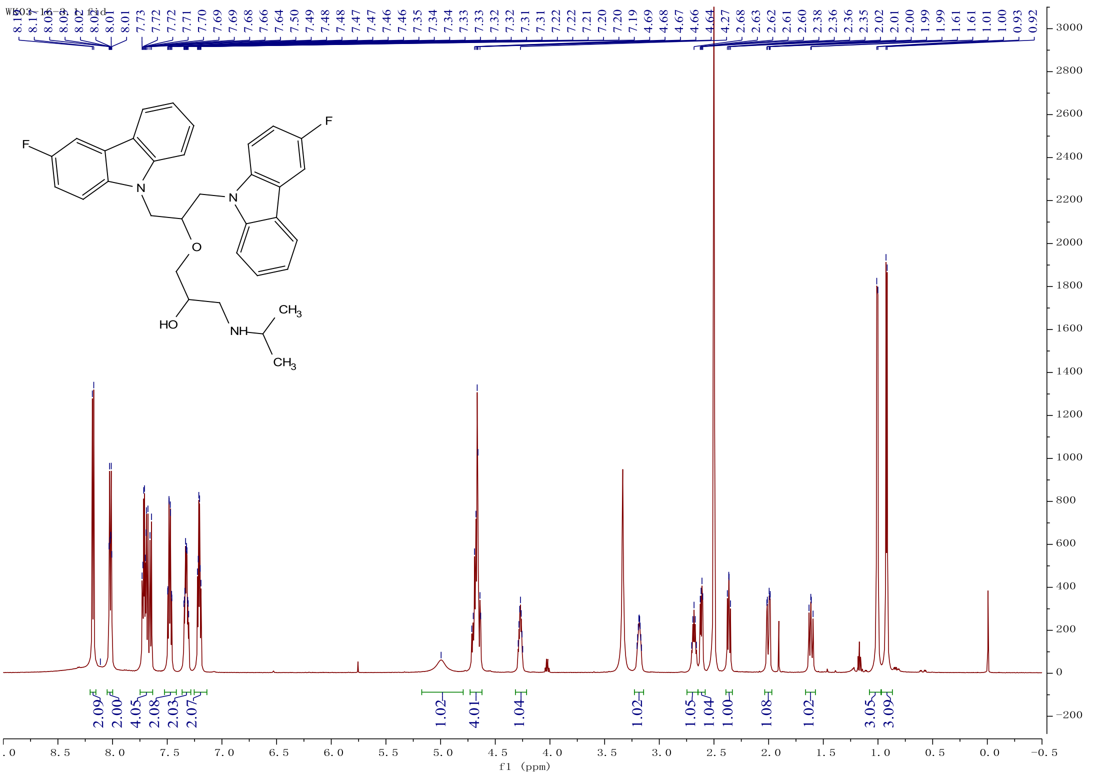

WK-23 ^{1}H NMR

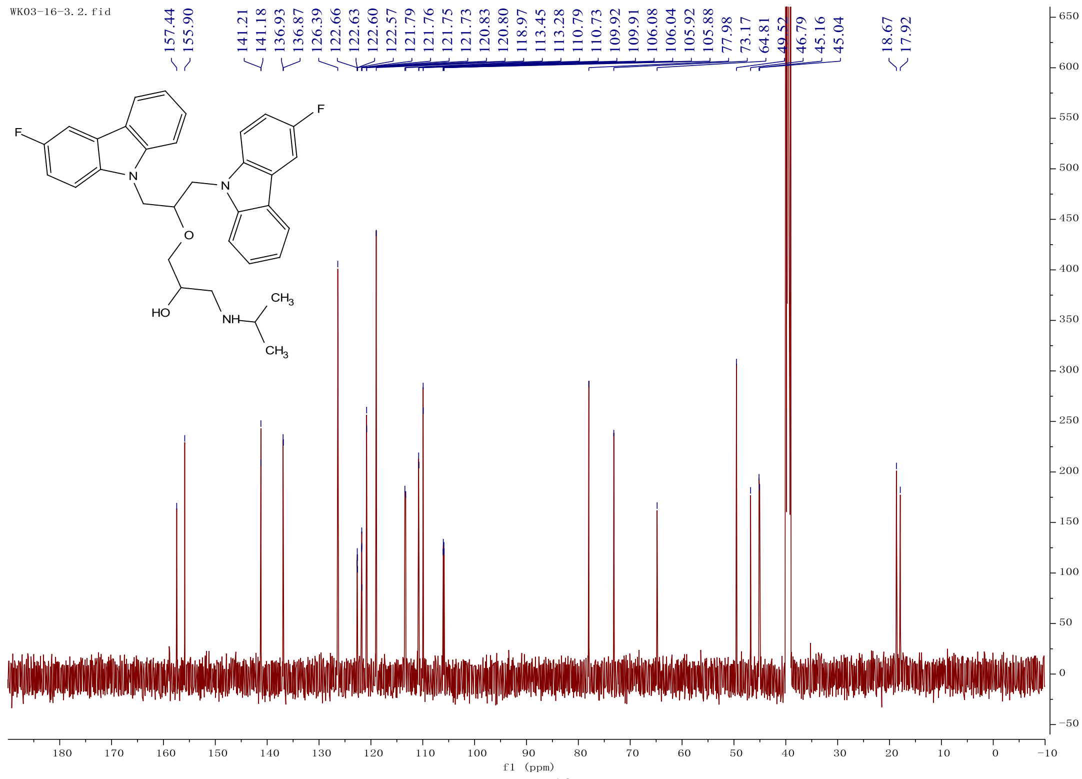

WK-23 ^{13}C NMR

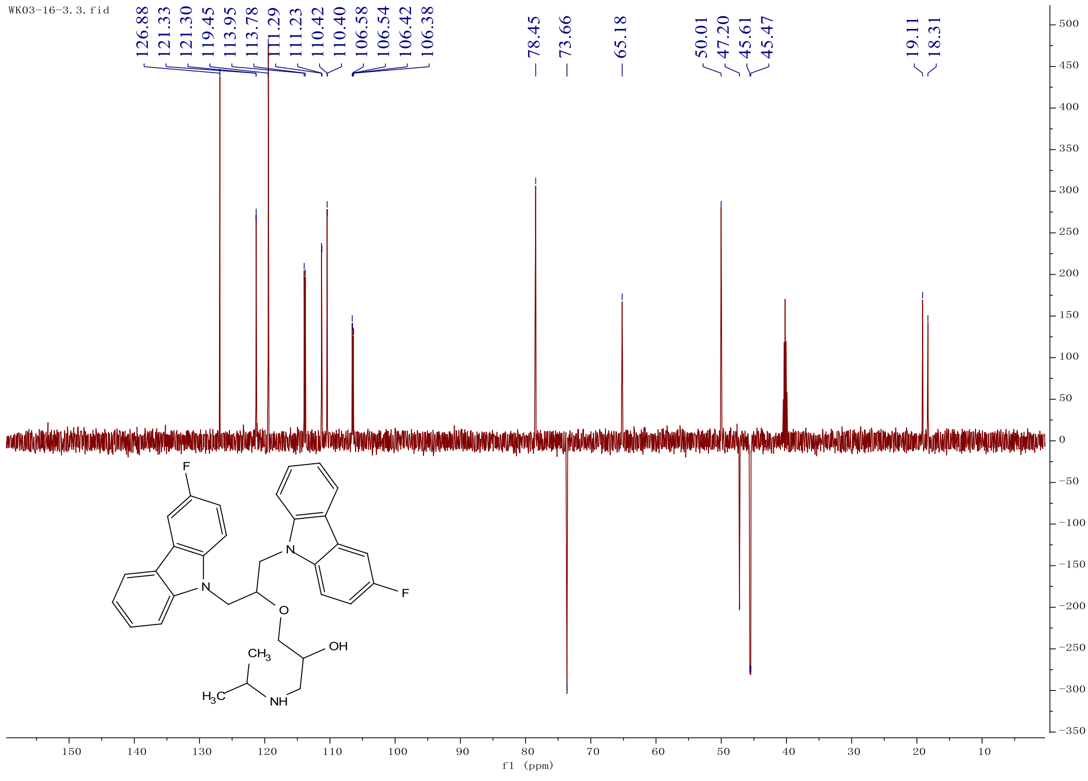

WK-23 Dept 135

***1-((1,3-bis(3-fluoro-9H-carbazol-9-yl)propan-2-yl)oxy)-3-(cyclopropylamino)propan-2-ol* (WK-24).**

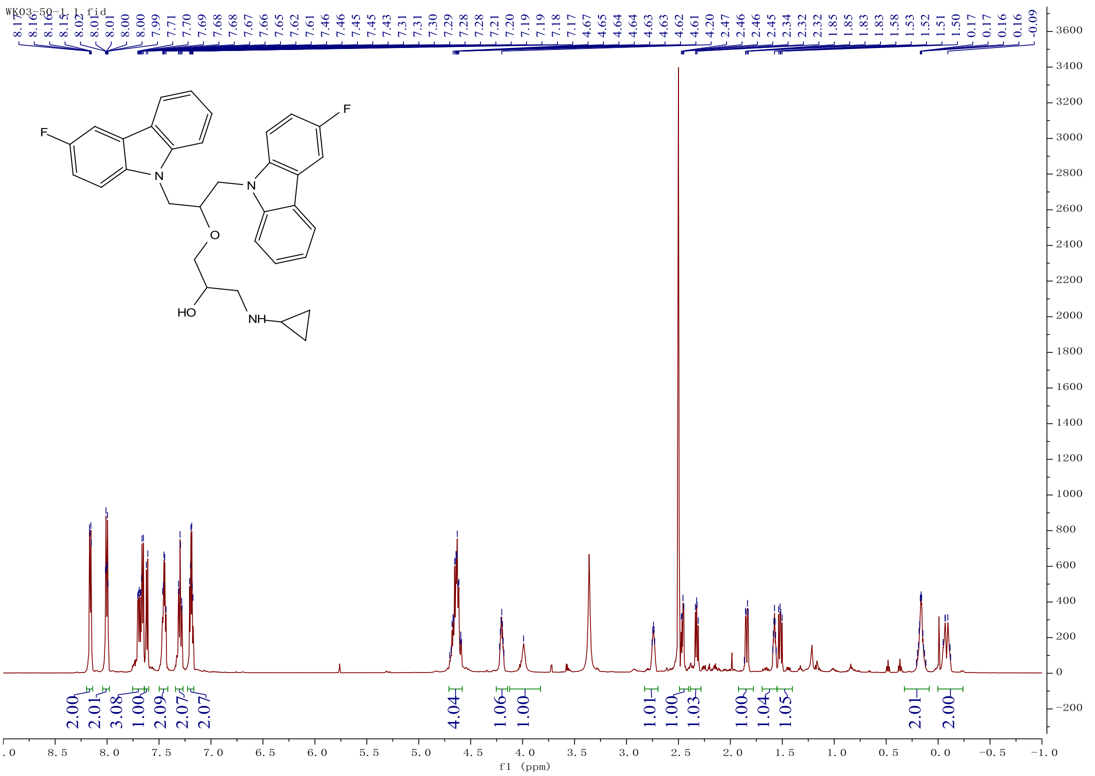

WK-24 ^{1}H NMR

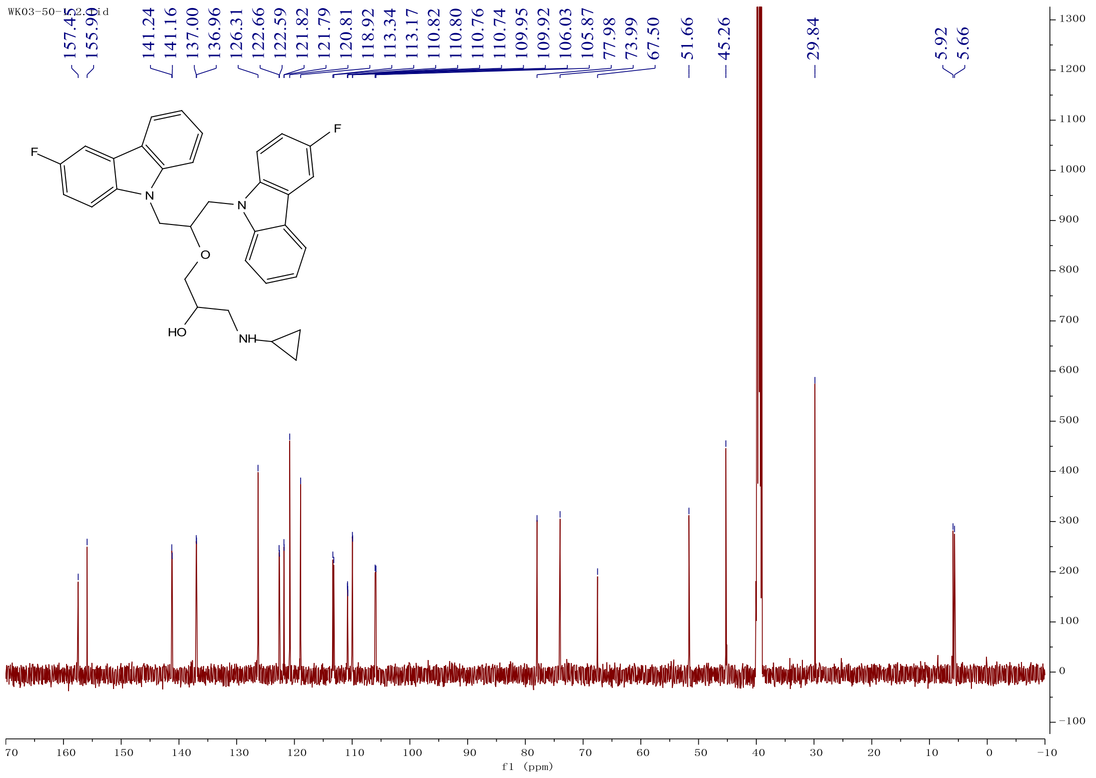

WK-24 ^{13}C NMR

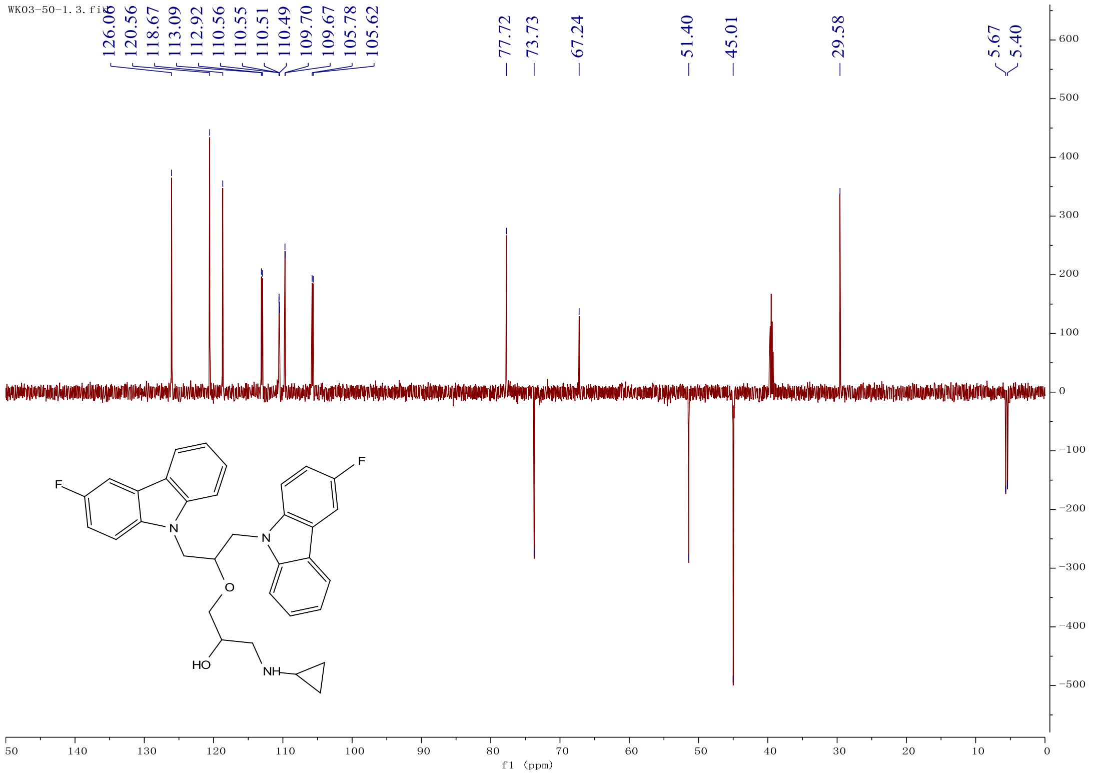

WK-24 Dept 135

***1-((1,3-bis(3-fluoro-9H-carbazol-9-yl)propan-2-yl)oxy)-3-(tert-butylamino)propan-2-ol* (WK-25).**

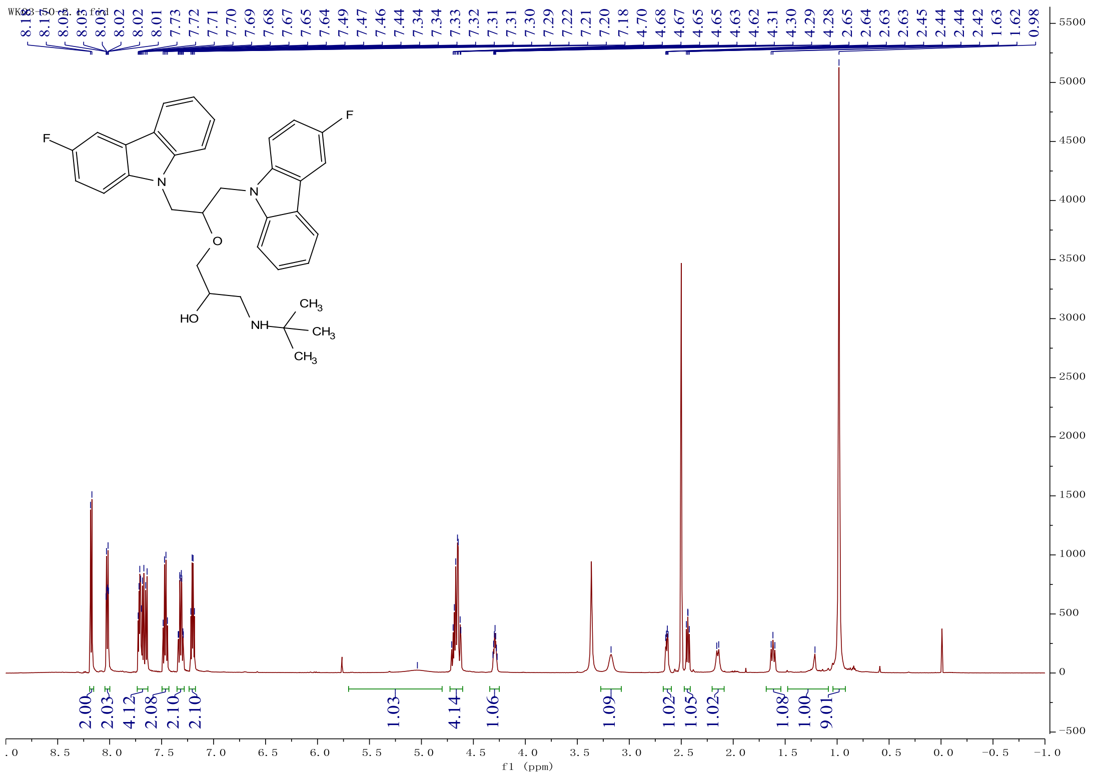

WK-25 ^{1}H NMR

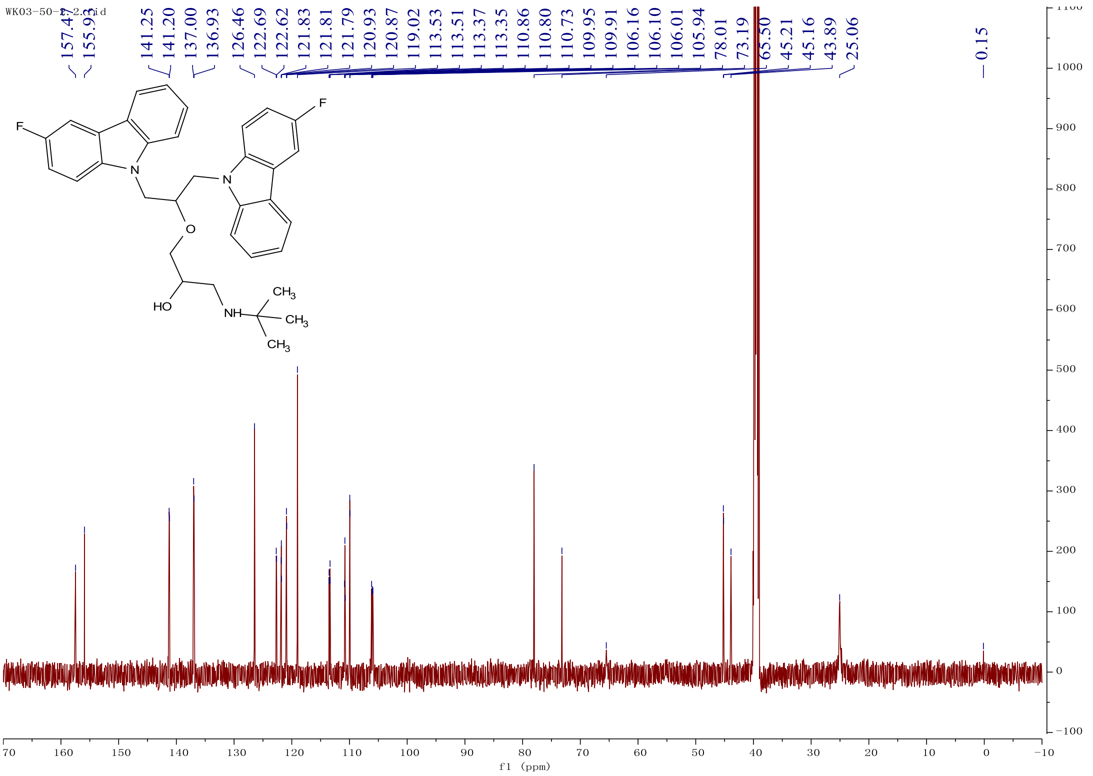

WK-25 ^{13}C NMR

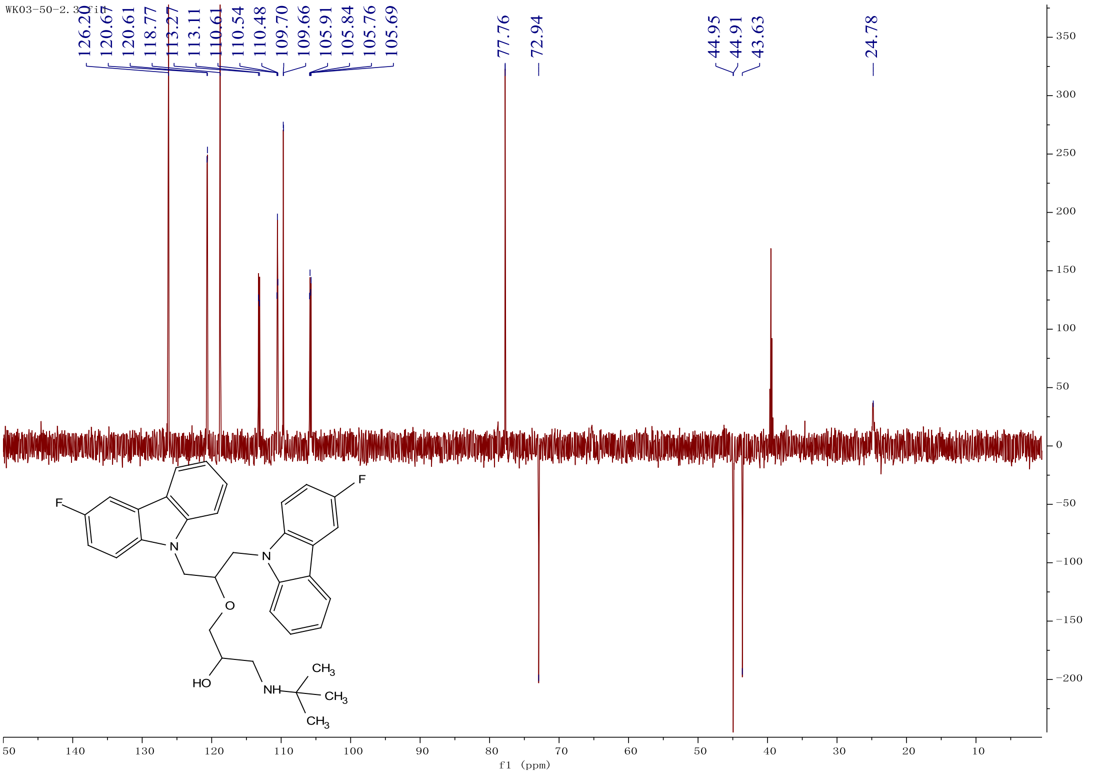

WK-25 Dept 135

***(2R)-tert-butyl 4-(3-((1-(3,6-difluoro-9H-carbazol-9-yl)-3-(3-fluoro-9H-carbazol-9-yl)propan-2-yl)oxy)-2-hydroxypropyl)-2-(hydroxymethyl)piperazine-1-carboxylate* (WK-26).**

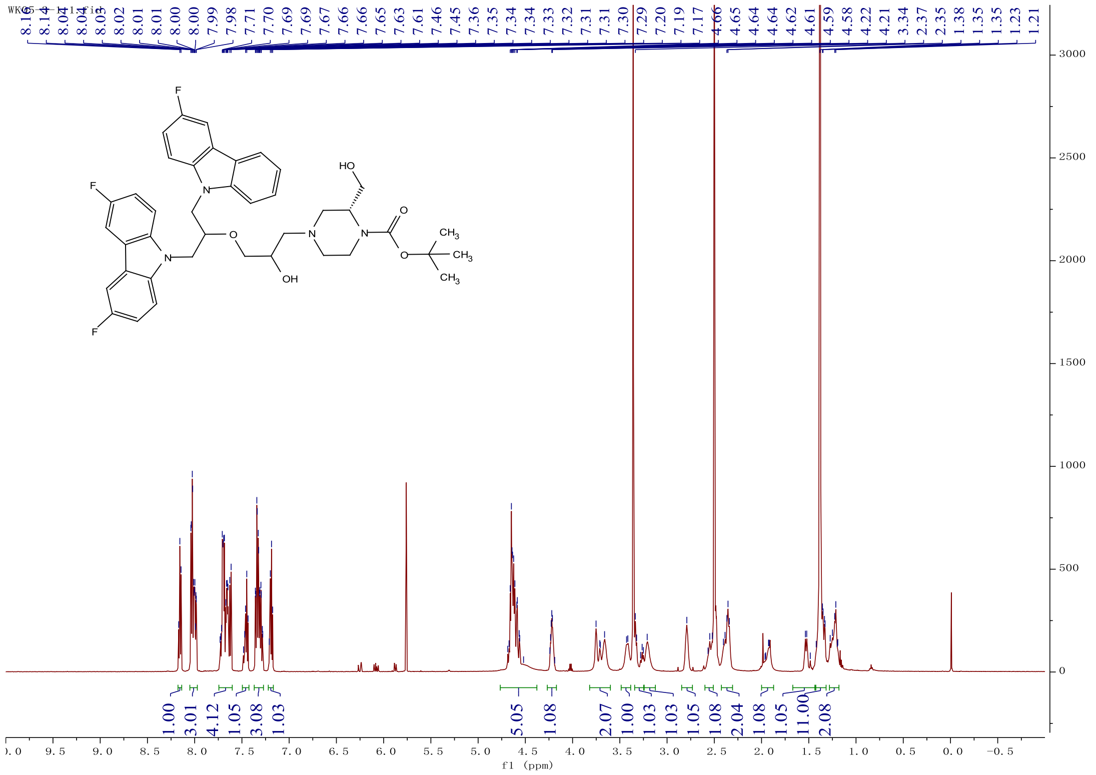

WK-26 ^{1}H NMR

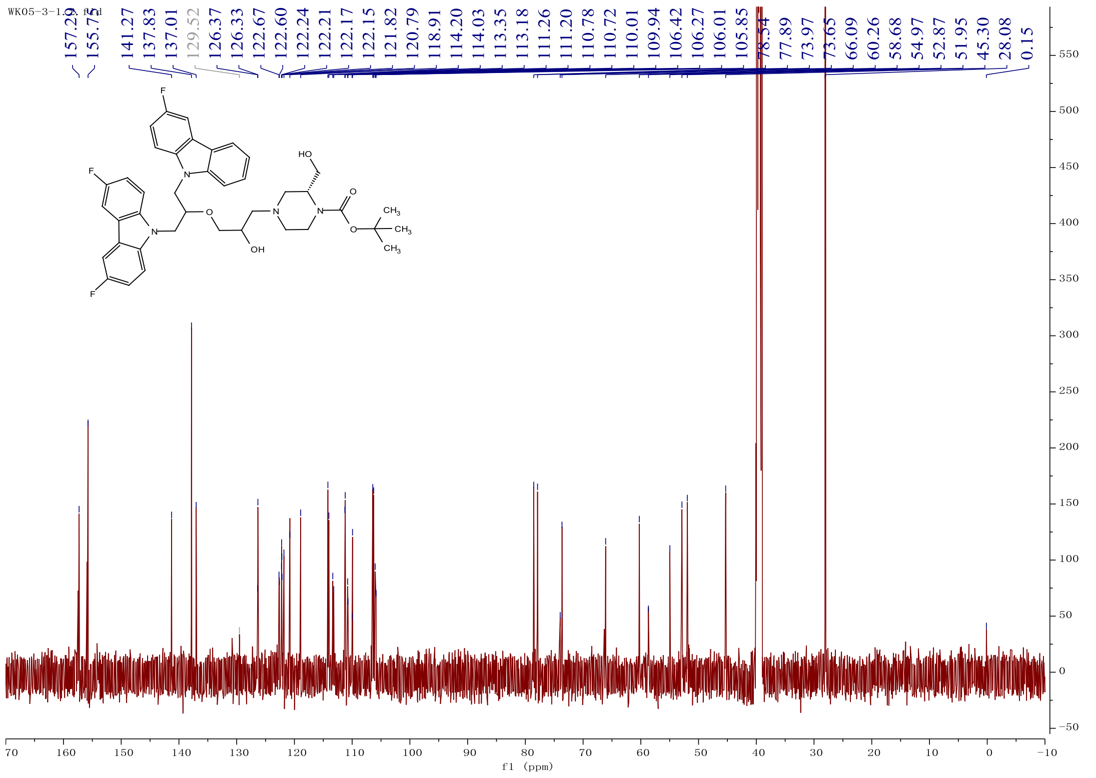

WK-26 ^{13}C NMR

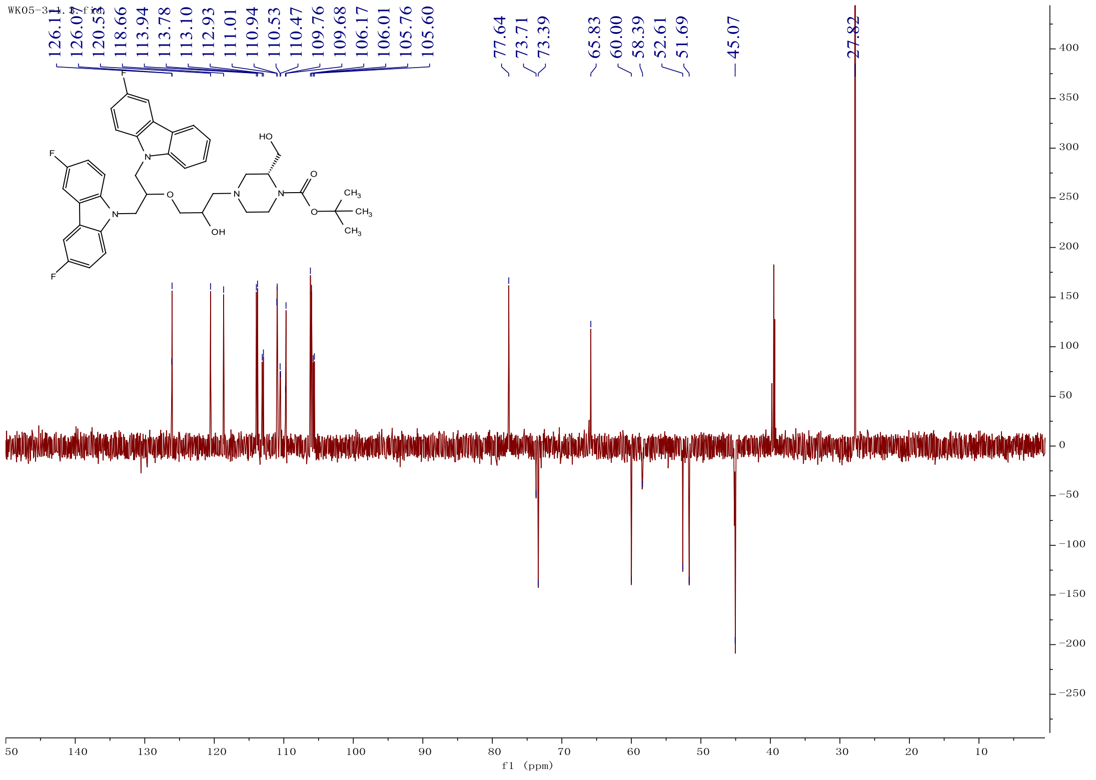

WK-26 Dept 135

***1-((1-(3,6-difluoro-9H-carbazol-9-yl)-3-(3-fluoro-9H-carbazol-9-yl)propan-2-yl)oxy)-3-((R)-3-(hydroxymethyl)piperazin-1-yl)propan-2-ol* (WK-27).**

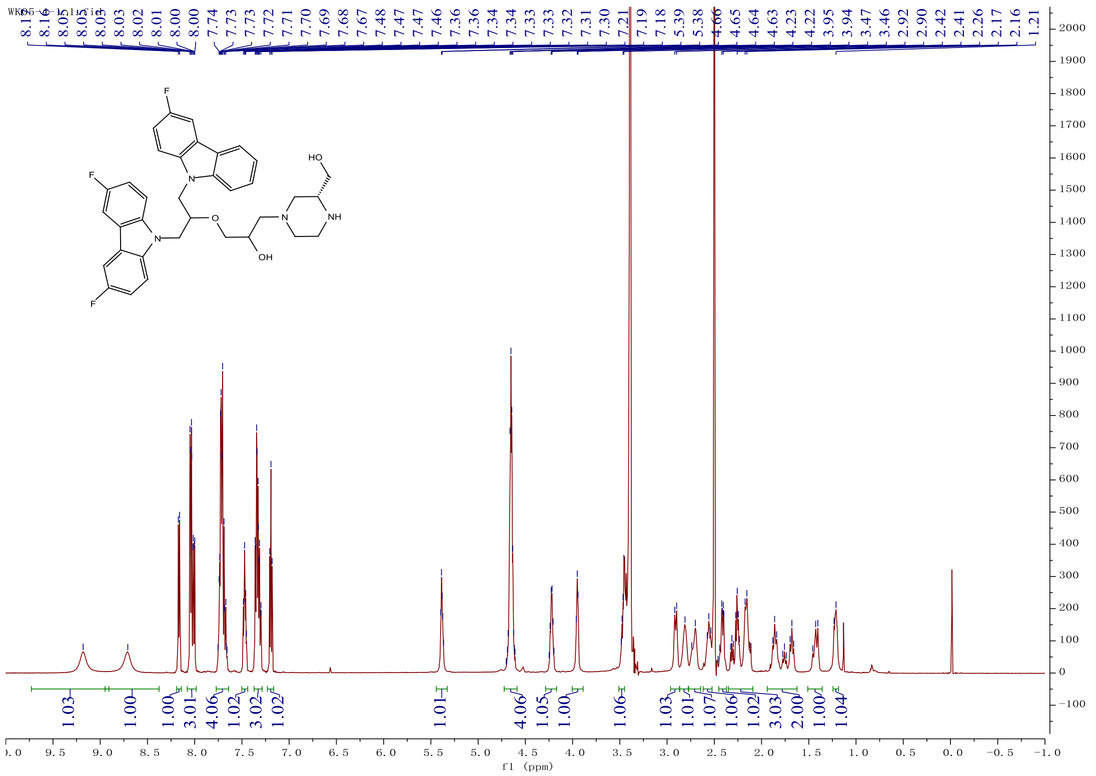

WK-27 ^{1}H NMR

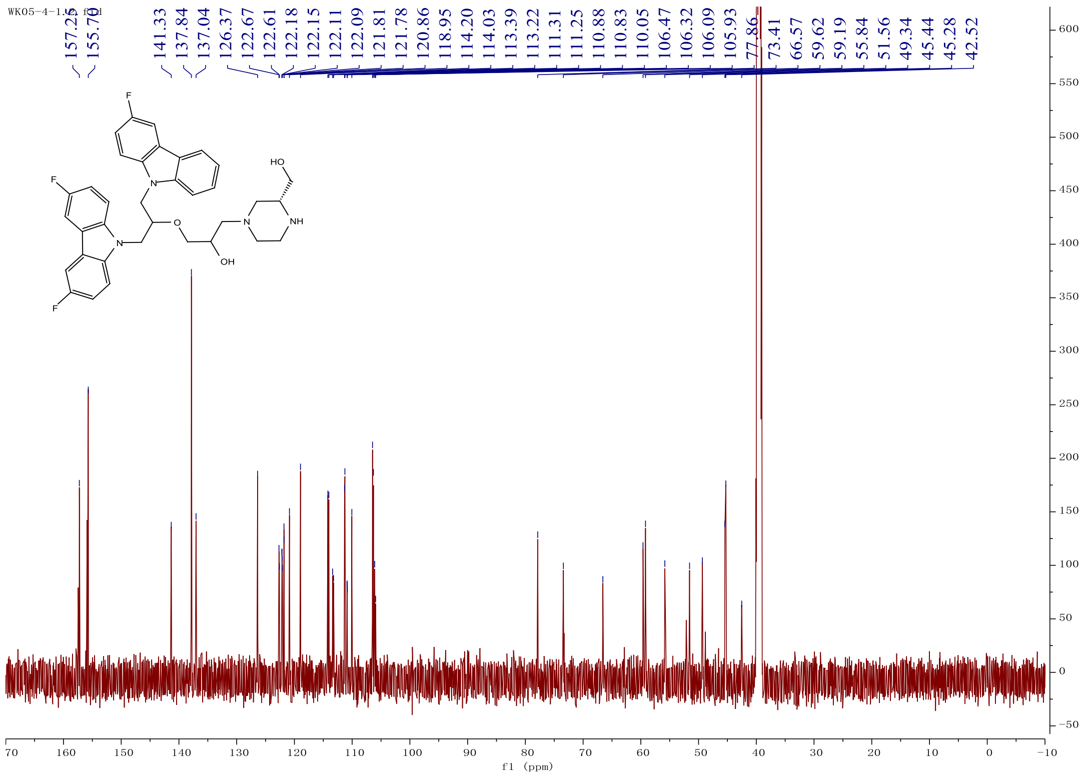

WK-27 ^{13}C NMR

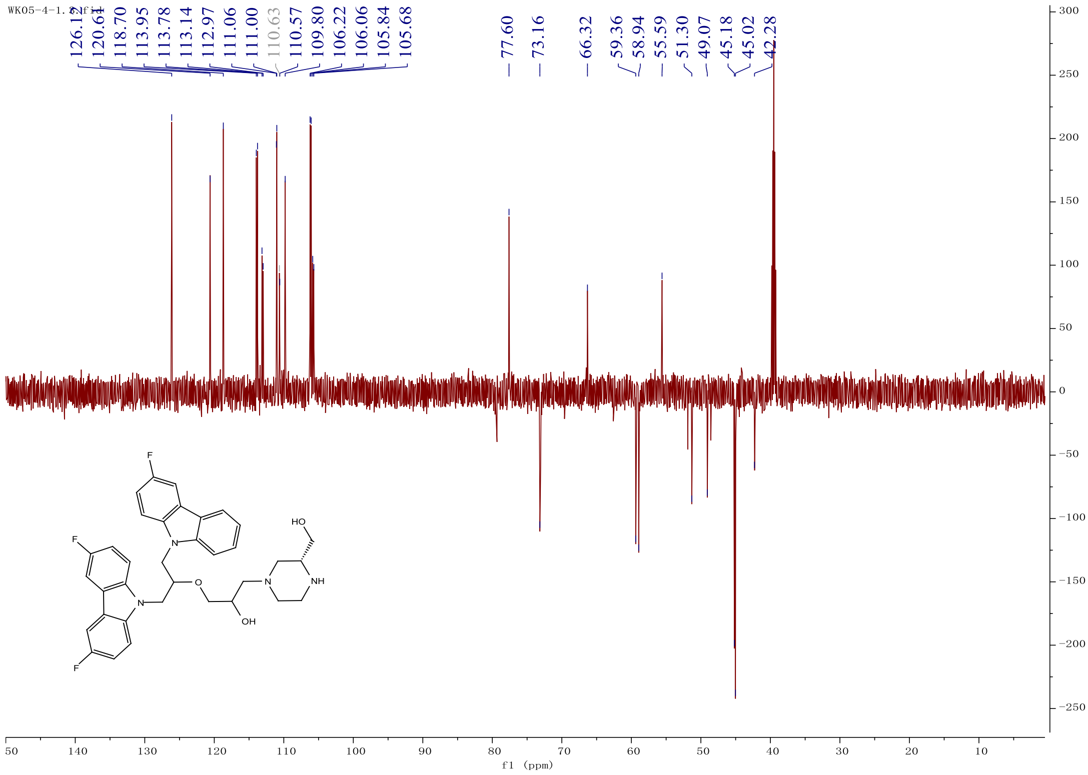

WK-27 Dept 135

***1-((2-(1H-indol-3-yl)ethyl)amino)-3-((1-(3,6-difluoro-9H-carbazol-9-yl)-3-(3-fluoro-9H-carbazol-9-yl)propan-2-yl)oxy)propan-2-ol* (WK-28).**

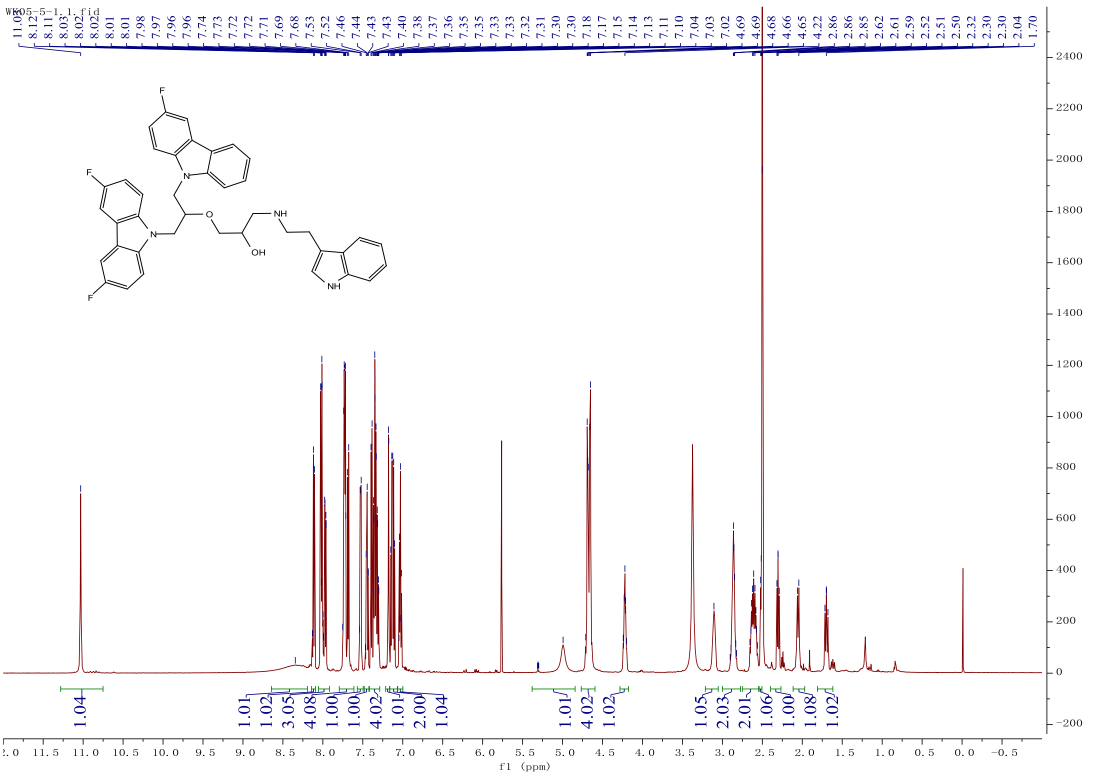

WK-28 ^{1}H NMR

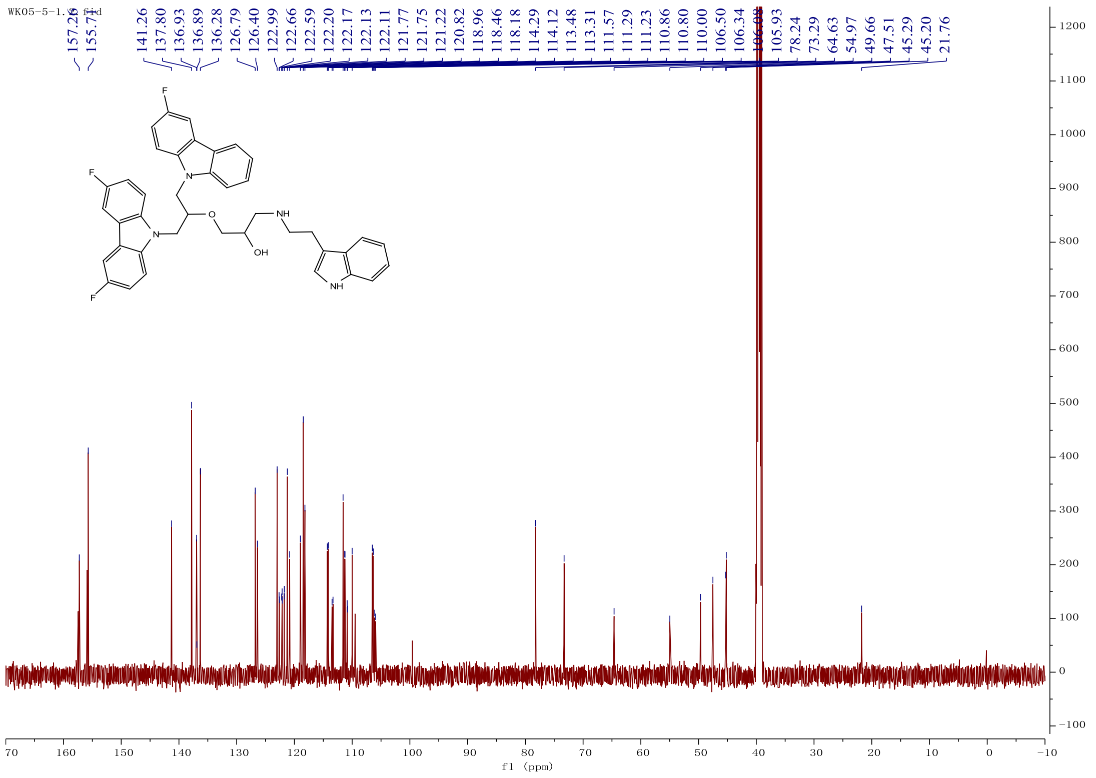

WK-28 ^{13}C NMR

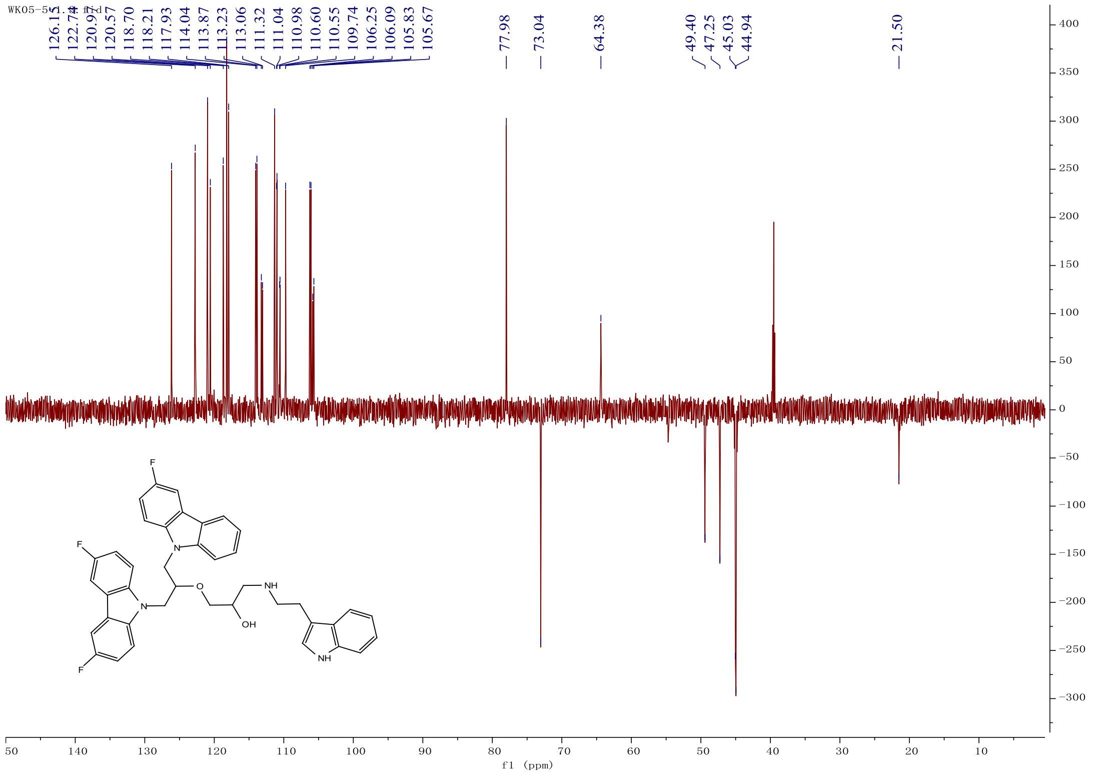

WK-28 Dept 135

***1-((3-butoxypropyl)amino)-3-((1-(3,6-difluoro-9H-carbazol-9-yl)-3-(3-fluoro-9H-carbazol-9-yl)propan-2-yl)oxy)propan-2-ol* (WK-29).**

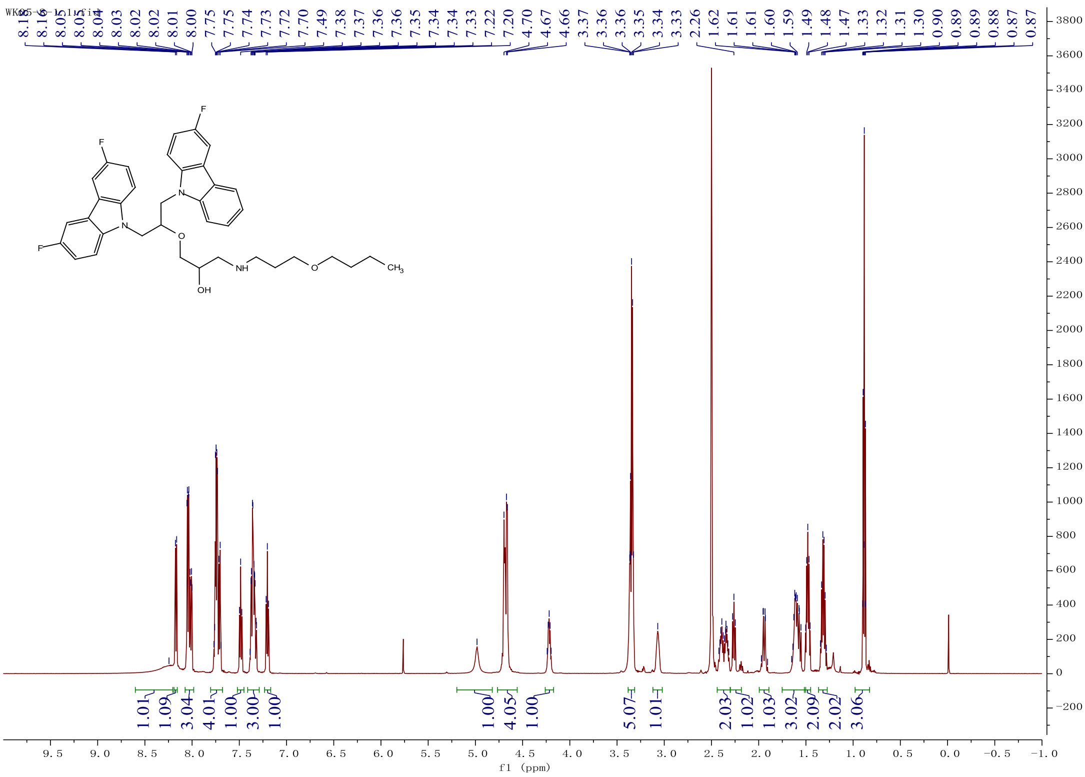

WK-29 ^{1}H NMR

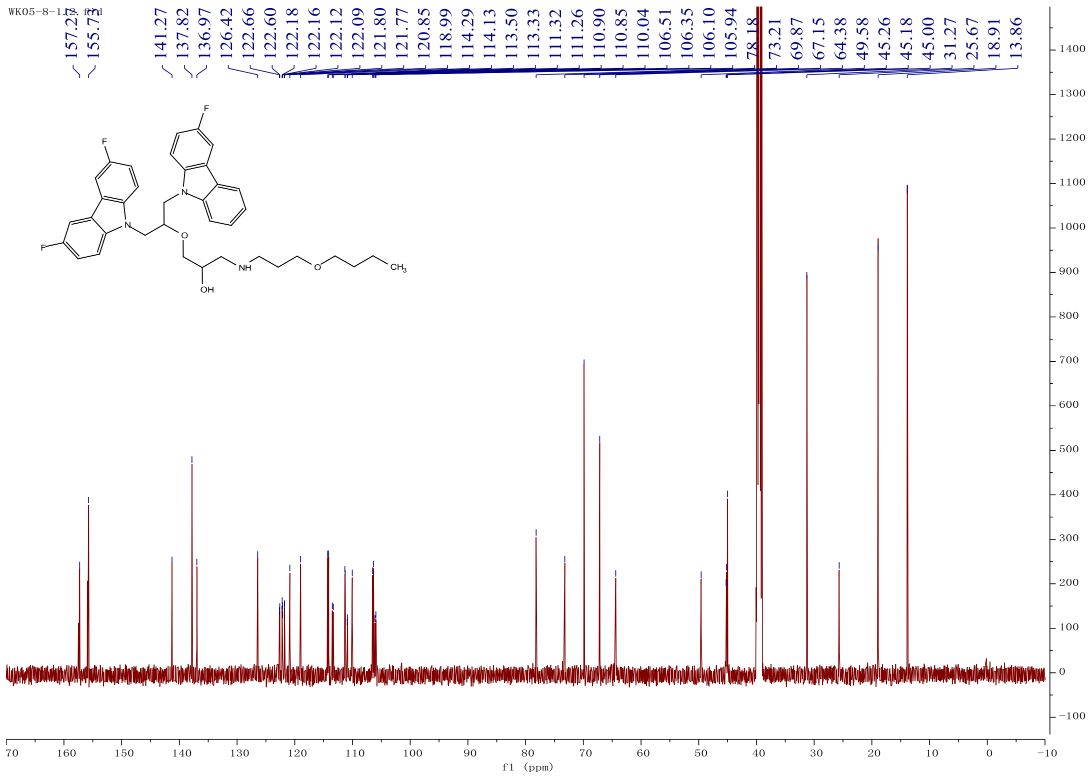

WK-29 ^{13}C NMR

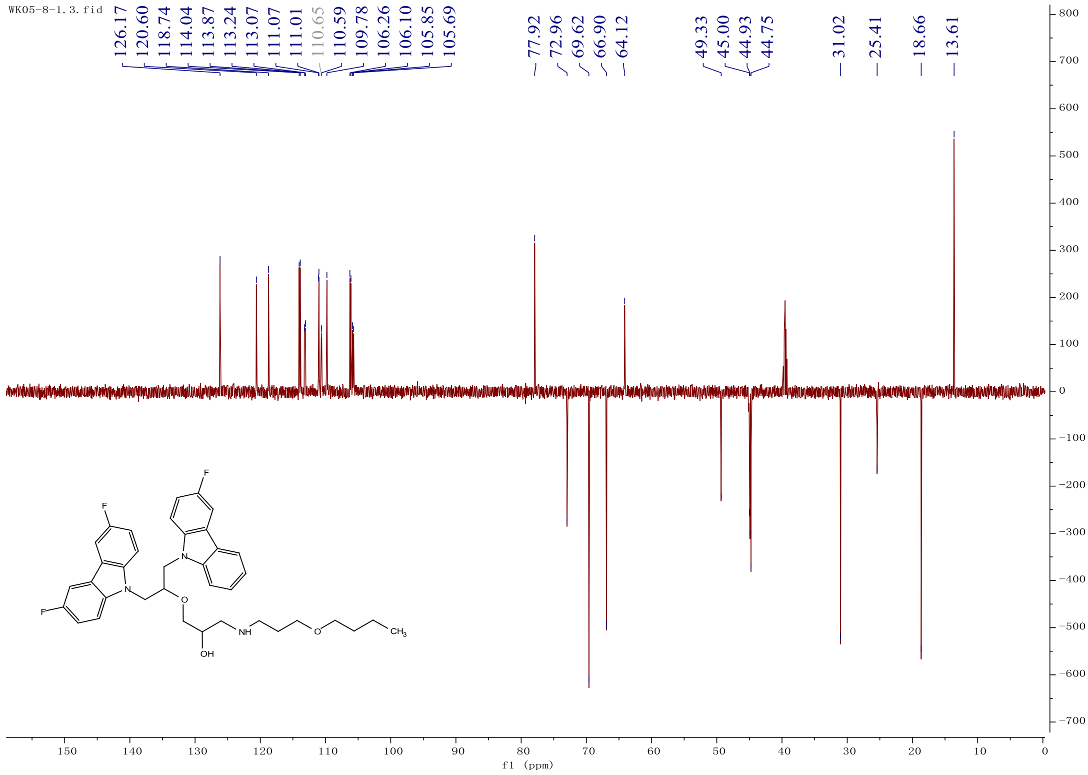

WK-29 Dept 135

1-((1-(3,6-difluoro-9H-carbazol-9-yl)-3-(3-fluoro-9H-carbazol-9-yl)propan-2-yl)oxy)-3-isopropoxypropan-2-ol **(WK-30).**

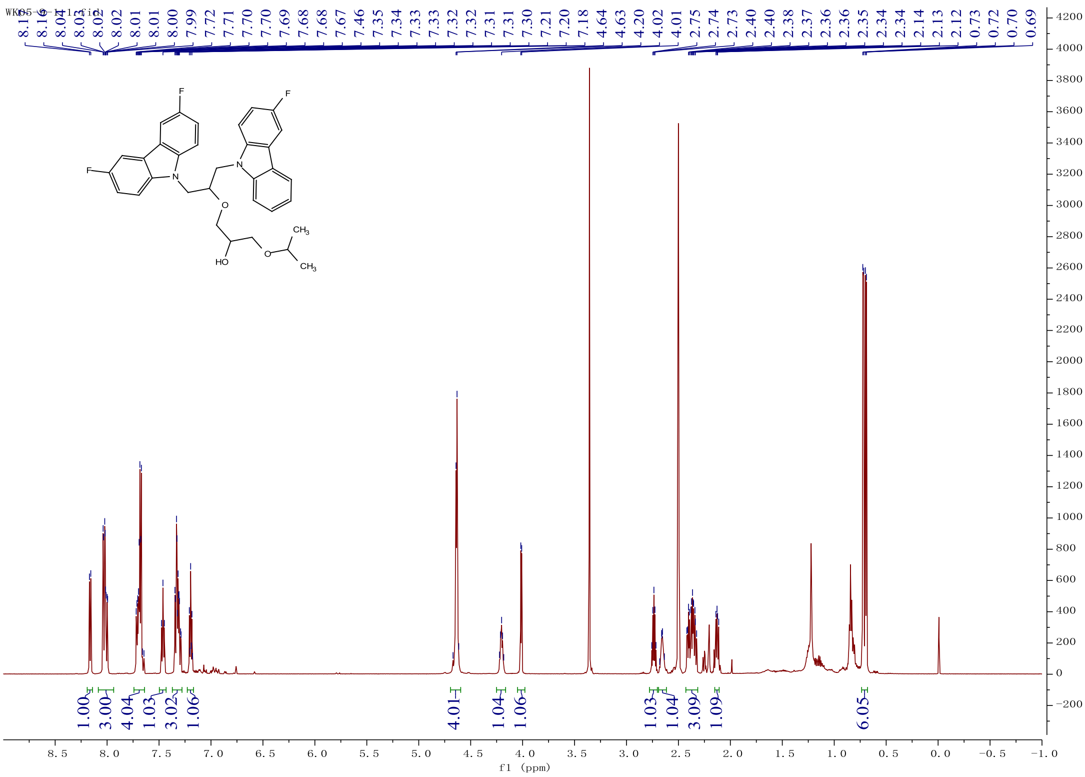

WK-30 ^{1}H NMR

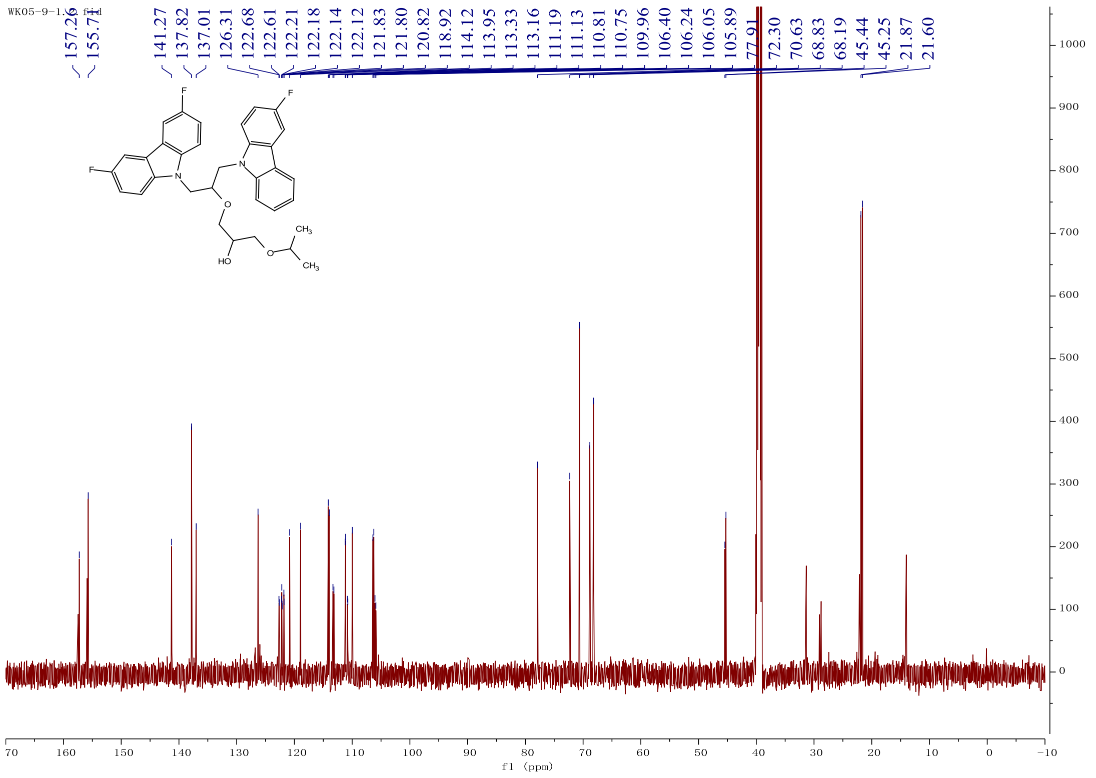

WK-30 ^{13}C NMR

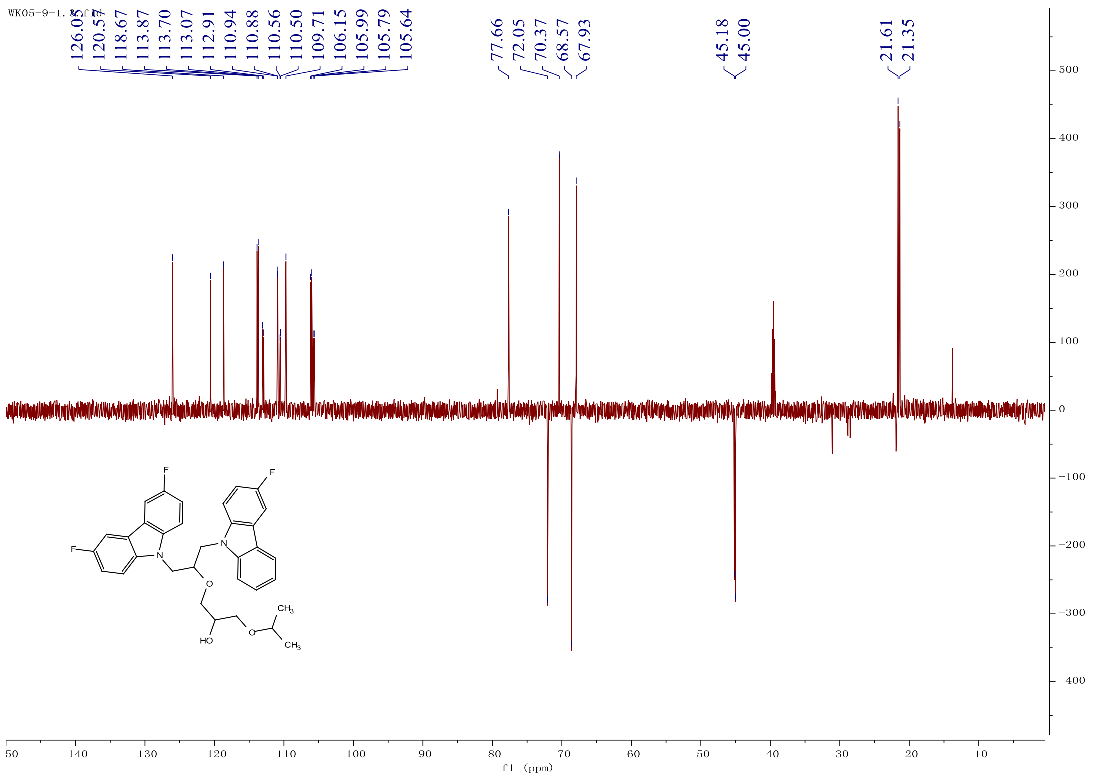

WK-30 Dept 135

Relative selectivity index

Relative selectivity index of DNMT1 is calculated by formula: DNMT1-selectivity index =1 - selected enzyme inhibition/DNMT1 inhibition. The closer that this value is to 1 or even more than 1, the better DNMT1-selectivity the of the compound is.

Table S1. Relative selectivity index of DNMT1

Cpd.	DNMT 3A/3L	DNMT 3B/3L	EZH2	LSD1	G9a
WK-1	0.49	1.15	0.93	0.18	0.84
WK-12	0.67	1.09	0.91	0.23	0.93
WK-13	0.68	1.28	1.01	0.33	0.91
WK-19	0.67	1.29	1.01	0.29	0.91
WK-22	0.04	0.03	0.96	0.02	0.9
WK-23	0.06	0.02	0.85	0.2	0.89
WK-25	0.94	1.44	0.89	0.02	0.99
WK-27	0.03	0.03	0.9	0.05	0.97

Docking validation

We docked SAH into DNMT1 structure (PDB 4WXX) according to the described method and validated the docking procedure by reproducing the SAH binding mode with a root-mean-square deviation (RMSD) of 0.965 Å.

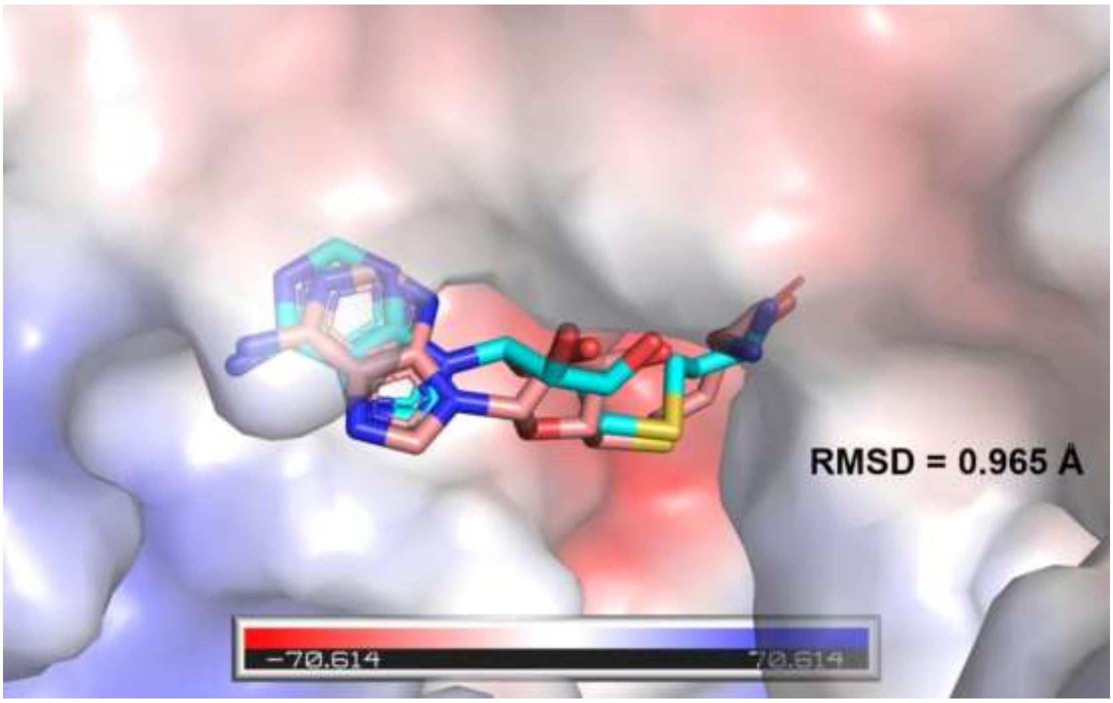

Figure S1. Validation of the molecular docking. SAH (salmon) was docked into the PDB 4WXX structure according to the described protocol and was found in the similar conformation as the SAH ligand (cyan) in the crystal structure. Oxygen atoms in SAH are shown in red, the sulfur in yellow and nitrogen in blue.
